# Human microglial transitions at the Aβ–tau inflection point associate with divergent pathways to dementia and resilience

**DOI:** 10.1038/s41591-026-04393-8

**Published:** 2026-06-04

**Authors:** Ashley Lu, Wei-Ting Chen, Maria Dalby, Diego Sainz Garcia, Marisa Vanheusden, Luuk E. de Vries, Veerle van Lieshout, Araks Martirosyan, Katleen Craessaerts, Sebastiaan Moonen, Magdalena Zielonka, Iordana Chrysidou, Anke Misbaer, Leen Wolfs, Benjamin Pavie, Dick Swaab, Dietmar Rudolf Thal, Inge Huitinga, Annemieke Rozemuller, Susan Karijn Rohde, Marc Hulsman, Henne Holstege, Rita Balice-Gordon, Niels Plath, Mark Fiers, Bart De Strooper

**Affiliations:** 1 Muna Therapeutics ApS, Copenhagen, Denmark; 2 Department of Neuroregeneration, Netherlands Institute for Neuroscience, Royal Netherlands Academy of Arts and Sciences, Amsterdam, The Netherlands; 3 VIB Center for Brain & Disease Research, VIB, Leuven, Belgium; 4 Department of Neurosciences and Leuven Brain Institute, KU Leuven, Leuven, Belgium; 5 VIB BioImaging Core Facility, VIB, Leuven, Belgium; 6 Department of Neuropsychiatric Disorders, Netherlands Institute for Neuroscience, Royal Netherlands Academy of Arts and Sciences, Amsterdam, The Netherlands; 7 Laboratory of Neuropathology, Department of Imaging and Pathology and Leuven Brain Institute, KU Leuven, Leuven, Belgium; 8 Department of Pathology, University Hospitals Leuven, Leuven, Belgium; 9 Netherlands Brain Bank, Netherlands Institute for Neuroscience, Amsterdam, The Netherlands; 10 Department of Pathology, Amsterdam University Medical Centre and Netherlands Brain Bank, Netherlands Institute for Neuroscience, Amsterdam, The Netherlands; 11 Department of Human Genetics, Genomics of Neurodegenerative Diseases and Aging, Vrije Universiteit Amsterdam, Amsterdam, The Netherlands; 12 Department of Pathology, Amsterdam UMC location VUmc, Amsterdam, The Netherlands; 13 Department of Human Genetics, KU Leuven, Leuven, Belgium; 14 UK Dementia Research Institute at UCL, University College London, London, UK

**Keywords:** Alzheimer's disease, Alzheimer's disease, Transcriptomics

## Abstract

Alzheimer’s disease (AD) is not an inevitable outcome of pathology but a dynamic process shaped by how brain cells respond to amyloid-β (Aβ) and tau. To disentangle these responses, we combined spatial transcriptomics and single-nucleus RNA sequencing of the superior frontal cortex from octogenarians living with or without dementia and from cognitively intact centenarians with comparable Aβ accumulation. We identified six distinct tissue domains representing a spatial pathological continuum of AD, with a key inflection point marked by a shift from Aβ-associated inflammatory changes to tau-associated cellular programs. This transition was accompanied by a change in microglial states, from early inflammatory to late antigen-presenting phenotypes, termed early and late plaque-induced gene (PIG) programs. Resilient individuals showed distinct pathological patterns: octogenarians without dementia lacked late PIGs, whereas centenarians showed late PIG activation that was uncoupled from tau accumulation. Together, these findings highlight divergent resilience-associated mechanisms in human aging and position microglial state transitions at the Aβ−tau interface as candidate points of resilience with potential therapeutic relevance.

## Abstract (web summary)

Microglia cellular changes underlie divergent resilience-associated mechanisms in human aging.

## Main

AD is a growing public health crisis^1^, now surpassing cancer in global prevalence. Despite recent advances in genetics, blood biomarkers and therapeutics^2–4^, the biological mechanisms that determine who develops dementia remain poorly understood. The field has long followed a linear ‘A-T-N’ staging model, in which Aβ deposition (A), precedes tau accumulation (T), neurodegeneration (N) and cognitive decline^5^. However, this cascade cannot explain the striking variability in clinical outcomes or the existence of individuals who remain cognitively intact despite advanced pathology.

Many centenarians, for example, exhibit abundant Aβ plaques yet maintain cognition, even in the presence of additional lesions such as α-synuclein and TDP-43 inclusions^6,7^. Similarly, a subset of octogenarians shows preserved cognition despite substantial Aβ burden and focal tau pathology. These observations challenge the classical amyloid cascade and suggest that adaptive cellular programs, particularly within the immune system, can transiently maintain neural homeostasis and delay cognitive decline^8,9^. Clinical symptoms may thus arise only when these compensatory mechanisms fail, crossing inflection points that shift the brain from adaptation to degeneration.

This nonlinear framework of AD progression^8,10,11^ has gained support from single-cell and spatial transcriptomic studies^12–23^. These studies show that microglia, astrocytes, oligodendrocytes and neurons undergo stage-specific transcriptional changes in response to Aβ, with microglial and immune pathways emerging as major genetic determinants of disease risk^24,25^. Early stages feature microglial activation^20,26–31^, oligodendrocyte remodeling^18,23,32^, interneuron loss^18,33,34^ and astrocyte hypoactivity^35^. These may buffer against damage but are followed by neuronal hyperactivity^18,36–38^, accelerated tau phosphorylation and widespread glial activation. Late-stage plaques are associated with neuritic phosphorylated tau (pTau)^39–42^ and excitatory neuron loss^18,34,43^. However, the molecular mechanisms linking Aβ-triggered immune responses to tau-mediated neurodegeneration remain unresolved.

The Seattle Alzheimer’s Disease (SEA-AD) atlas^34^ is a large, multidonor single-nucleus RNA sequencing (snRNA-seq) resource generated from human frontal and temporal cortex, covering the full spectrum of AD pathology and incorporating harmonized cell type annotations across hundreds of thousands of nuclei. The ROSMAP cohort^20^ combines longitudinal clinical data from older adults with postmortem molecular profiling, including extensive snRNA-seq and detailed neuropathological assessments. These datasets have been central to defining disease-associated cell states in AD, but neither includes spatial transcriptomic information, which is essential because AD brains contain a mosaic of local pathological states within the same tissue^20,34^. Spatial transcriptomics enables these transitions to be resolved in situ, directly linking cellular states to local Aβ and tau pathology. However, large autopsy datasets are often confounded by age-related variability and co-pathologies.

To overcome these limitations, we selected 24 well-characterized octogenarian brains (OCT cohort), matched for age, sex and *APOE* status and free of major non-AD lesions, and a complementary second cohort of 20 centenarian brains from the Dutch 100-plus Study^44–46^—included to examine resilience at extreme ages and across a range of cognitive outcomes rather than to extend the octogenarian disease trajectory. We applied Visium spatial transcriptomics and snRNA-seq of the superior frontal gyrus, complemented by orthogonal validation using Xenium in situ hybridization.

Together, these data support a key immune inflection point, marked by a transition from early PIG programs^23^ to a late-stage antigen-presenting microglial phenotype associated with tau pathology. Our study indicates that resilience emerges through distinct trajectories: octogenarians without dementia mounted only early PIG responses despite Aβ burden, whereas cognitively intact centenarians displayed late PIG activation yet resisted downstream tau accumulation. These findings highlight microglial modulation at the Aβ−tau interface transition as a critical determinant of resilience and a promising therapeutic target in human AD.

## Results

### Spatial and single-nucleus transcriptomics across octogenarian and centenarian brains to dissect resilience

**Fig. 1:**
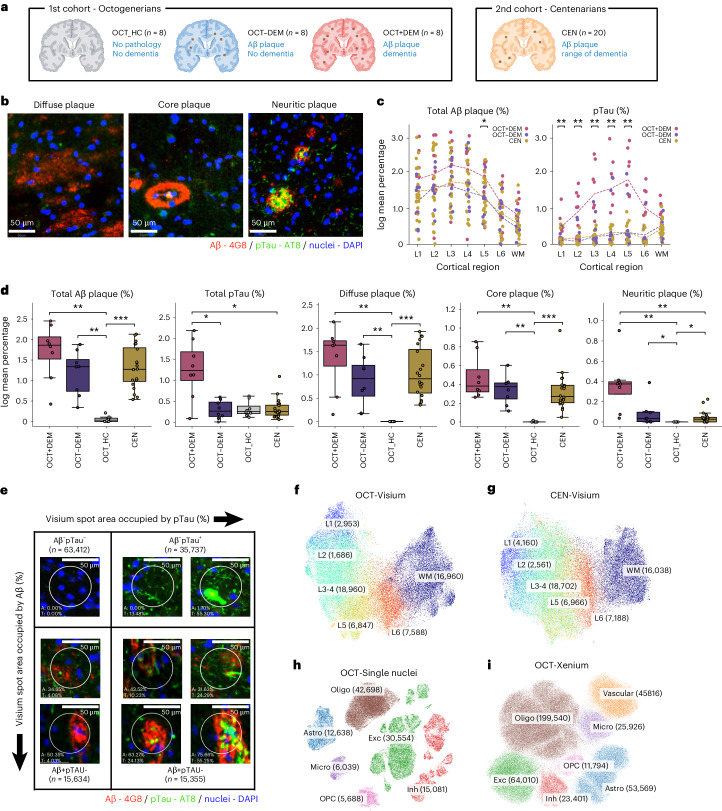
Pathological Visium spots in the brains of octogenarian and centenarian patients. **a**, Overview of the octogenarian (OCT) and centenarian (CEN) cohorts. The OCT cohort, designed to dissect cellular responses associated with AD pathology and cognitive decline, includes 24 individuals: eight healthy controls (OCT_HC), eight with AD pathology but no dementia (OCT − DEM) and eight with both pathology and living with dementia (OCT + DEM). The CEN cohort, included to explore mechanisms underlying cognitive resilience in advanced aging, comprises 20 centenarians with high Aβ burden and variable co-pathologies. **b**, Representative immunofluorescence images of diffuse—sharply delineated, extracellular Aβ aggregates within the cortical neuropil that did not contain a core of amyloid material^59^; cored—cortical amyloid plaques with a compact, central amyloid core and a halo of diffuse, extracellular Aβ aggregates^59^; and neuritic—diffuse or cored plaques that contain pTau^+^ dystrophic neurites^59^. In Extended Data Fig. 1b, a quantification over the different layers is shown. **c**, Layer-specific distribution of pathology across groups: OCT + DEM (pink), OCT − DEM (purple) and CEN (yellow). Aβ plaque and pTau pathology levels are shown as log-transformed mean percentages per cortical layer for each individual donor. Group differences within each layer were assessed using a permutation-based ANOVA, in which phenotype labels were randomly reassigned within layers to generate a null distribution (5,000 permutations). Empirical *P* values were Bonferroni corrected across layers. Asterisks indicate layers showing significant differences between phenotype groups (*P_adj_ < 0.05, **P_adj_ < 0.01, ***P_adj_ < 0.001). **d**, Quantification of Aβ and pTau profiles across cohorts. Aβ plaque levels (total and core, diffuse) are similar in OCT + DEM, OCT − DEM and CEN, whereas pTau burden and neuritic plaques are significantly elevated in OCT + DEM. Each dot shows the pathology levels as the mean log-transformed percentages of affected area over all Visium spots per participant. Box plots show median, IQR and whiskers extending to 1.5× IQR. Colors indicate the cohort. Statistical significance was determined using a two-sided Mann−Whitney *U*-test for pairwise comparisons between phenotypic groups, with Bonferroni correction: *P_adj_ < 0.05, **P_adj_ < 0.01, ***P_adj_ < 0.001. **e**, Visium spots reveal a spatial continuum from Aβ^−^pTau^−^ to Aβ^+^pTau^+^ pathology. Representative immunofluorescence images illustrate a progressive continuum of pathology, from spots lacking detectable Aβ and pTau (Aβ^−^pTau^−^) to those showing combined pathology (Aβ^+^pTau^+^). Each panel represents one pathology state, with the number of Visium spots per category shown at the top and bottom of the figure. White circles mark the actual Visium spot boundaries (55-µm diameter). Percentages (A: Aβ; T: pTau) indicate the proportion of spot area occupied by each pathology. Scale bar, 50 µm. **f**,**g**, UMAP of Visium spots. In total, 60,129 Visium spots from OCT (**f**) and 61,739 Visium spots from CEN (**g**) segregate by cortical layer and white matter, confirming spatially defined transcriptomic signatures. **h**,**i**, UMAP of snRNA-seq profiles from 112,698 snRNA-seq across 24 OCT cases (**h**), classified into six major cell types: oligodendrocytes (Oligo), astrocytes (Astro), microglia (Micro), oligodendrocyte precursor cells (OPC), excitatory neurons (Exc) and inhibitory neurons (Inh). Vascular cells were excluded due to the low number recovered. UMAP of Xenium pseudocell profiles (**i**). In total, 424,056 pseudocells from 11 OCT cases (five OCT + DEM, six OCT − DEM) were annotated using 329 genes across seven cell types (same as in the snRNA UMAP but including vascular cells). Panel **a** created in BioRender; Cherretté, E. https://biorender.com/opvgchp (2026). WM, white matter.

**Extended Data Fig. 1:**
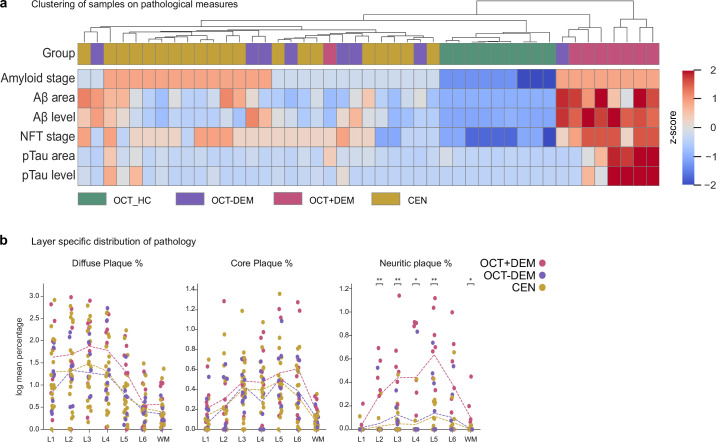
Neuropathological features and quantitative analysis across cohorts. (**a**) **Hierarchical Clustering of neuropathological features across all individuals**. Heatmap shows hierarchical clustering (Euclidean distance, average linkage) of all 44 donors from the OCT and CEN cohorts using combined neuropathology metrics. Annotations (top to bottom) indicate: (1) experimental group (OCT + DEM: pink, OCT-DEM: purple, OCT_HC: green, and CEN: brown. (2) Amyloid stage (ABC derived Thal Aβ phase). (3) Aβ burden in Visium sections (% area overlap with 4G8 signal). (4) Aβ burden in DAB-stained sections (4G8). (5) Neurofibrillary tangle stage (NFT, ABC-derived Braak stage). (6) pTau burden in Visium sections (% overlap with AT8 signal). (7) pTau burden in DAB stained sections (AT8). CEN and OCT-DEM cases cluster together reflecting comparable Aβ and pTau profiles, while OCT + DEM and OCT_HC form distinct branches. (**b**) **Layer specific distribution of diffuse, core and neuritic plaques**. Different plaque categories were manually annotated (see Methods). This plot shows the average percentage of immunostaining overlap of Visium spots with regions annotated as diffuse (left), core (center) and neuritic (right) plaques across the cortical layers and white matter. In particular neuritic plaques show a pronounced concentration in layers 3-5, but only in the OCT + DEM cohort, equivalent to the pTau load (Fig. 1c). Layer-wise comparisons between diagnostic groups were assessed using a permutation-based ANOVA for each cortical layer, with Bonferroni correction applied across layers. Adjusted p-values < 0.05 was considered statistically significant.

We analyzed two well-characterized human brain cohorts from the Netherlands Brain Bank (NBB) (Fig. 1a–d and Extended Data Fig. 1). The first cohort, termed ‘OCT’, comprised 24 octogenarian donors selected to minimize comorbid pathology beyond Aβ plaques and neurofibrillary tangles. It included eight individuals living with dementia (OCT + DEM), eight individuals with substantial Aβ pathology but preserved cognition (OCT − DEM) and eight cognitively healthy controls (OCT_HC), matched for age, sex and *APOE* genotype (Supplementary Table 1a). This design captured a continuum from normal cortical architecture to Aβ-associated and tau-associated inflammation and degeneration.

To examine resilience mechanisms in advanced aging, we analyzed a complementary cohort of 20 centenarian (CEN) brains from the Dutch 100-plus Study^6,45,47^. All centenarians exhibited high Aβ plaque load but variable pTau pathology and frequent co-pathologies, including cerebral amyloid angiopathy (20/20), TDP-43 inclusions (13/20), hippocampal sclerosis (7/20) and Lewy body pathology (4/20) (Supplementary Table 1b). Cognitive performance assessed near death (Mini-Mental State Examination (MMSE) range 8−29, mean: 22.4 ± 5.6) varied widely and was not explained by any single pathological feature^7^. All individuals had been cognitively intact at enrollment, supporting the presence of endogenous resilience mechanisms.

From both cohorts, we sampled the superior frontal gyrus using 8-mm biopsy punches encompassing all six cortical layers. Visium spatial transcriptomics was performed on 10-µm cryosections yielding transcriptomic profiles from 55-µm-diameter circular Visium spots. Pathology staining, performed on the same tissue section, was quantified within each spot as the percentage overlap between the Visium spot area and segmented regions based on the immunofluorescent signal for Aβ (4G8) and pTau (AT8) (Fig. 1e and Supplementary Tables 1 and 2). This continuous, spatially resolved measure replaced categorical plaque annotations (diffuse, cored and neuritic; Fig. 1b), allowing quantitative modeling of pathology–transcriptome relationships (Fig. 1e and Supplementary Tables 1 and 2). The Visium transcriptomic analysis was performed on the CEN cohort and the OCT cohort with the same workflow, generating 60,129 and 61,739 annotated Visium spots, respectively.

To complement spatial data (Fig. 1f,g) we generated snRNA-seq profiles from 112.698 nuclei isolated from adjacent 750-µm sections from the OCT cohort using the 10x Chromium platform (Fig. 1h, Supplementary Fig. 1 and Supplementary Data 1). In parallel, Xenium in situ hybridization was applied to 10-µm sections from 11 OCT brains, yielding 424,056 segmented pseudocells with single-molecule resolution and spatial coordinates (Fig. 1i).

### Tau pathology differentiates cognitive status among Aβ-plaque-positive octogenarians

Neuropathological assessment using the National Institute on Aging−Alzheimer’s Association (NIA−AA) ABC criteria (A: Thal Aβ phase; B: Braak neurofibrillary tangle stage; C: CERAD neuritic plaque score; 0 = none, 3 = severe) identified pTau pathology as the principal correlate of dementia in the octogenarian cohort. OCT + DEM brains exhibited significantly higher Braak scores (mean = 2.9 ± 0.35) than both OCT − DEM (2.1 ± 0.35, Mann−Whitney *P* = 0.04) and centenarians (2.15 ± 0.6, *P* = 0.005). Quantitative immunostaining confirmed this pattern, showing greater overlap between pTau signal and Visium spots in OCT + DEM than in other groups (Fig. 1d). By contrast, Aβ plaque burden did not differ significantly between OCT − DEM versus OCT + DEM (Fig. 1d), indicating that tau pathology, not amyloid load, best distinguishes individuals living with dementia from those who remain cognitively intact. Consistent with this, OCT + DEM brains displayed denser neuritic plaques (mean C-score = 2.75 ± 0.71) and pronounced tau accumulation in cortical layers II–V (Fig. 1c,d). Thus, pTau, but not Aβ, is the primary pathological correlate of dementia in the OCT cohort.

Hierarchical clustering of Visium pathology metrics and ABC staging, here used only for descriptive visualization, showed that centenarians grouped with OCT − DEM cases, whereas OCT + DEM and OCT_HC formed separate branches (Extended Data Fig. 1a). This illustrates that preserved cognition in centenarians occurs despite substantial Aβ deposition and markedly lower pTau burden.

### Six distinct tissue domains define AD progression in octogenarian brains

Given the high comorbidity burden in centenarians, we first focused on the octogenarian brains, which displayed the core pathological characteristics of AD— that is, Aβ plaques and pTau neurofibrillary tangles without major co-pathologies. We performed Visium spatial transcriptomics on 60,129 Visium spots from 24 OCT individuals across three phenotypes: cognitively healthy (OCT_HC; 19,788 spots), individuals living with dementia (OCT + DEM; 20,050 spots) and individuals without dementia but with pathology (OCT − DEM; 20,291 spots). Each 55-µm Visium spot captured a median of 1,831 genes (interquartile range (IQR): 1,168−2,352). Approximately 72% of Visium spots localized to gray matter with a layer distribution consistent with cortical anatomy. Because white matter content was highly variable across samples due to differences in dissection depth, quantitative analyses were restricted to gray matter. Within the gray matter, layers 3–5 showed the highest pTau accumulation and the strongest Aβ overlap across individuals, identifying them as the most informative region to study the Aβ–tau interface (Fig. 1c and Extended Data Fig. 1b).

From adjacent sections, we generated 112,698 single-nucleus transcriptomes (mean 3,399 genes per nucleus, IQR: 1,563−4,663), identifying all major brain cell types and 50 fine-grained cellular subtypes (Supplementary Fig. 1 and Supplementary Data 1).

**Fig. 2:**
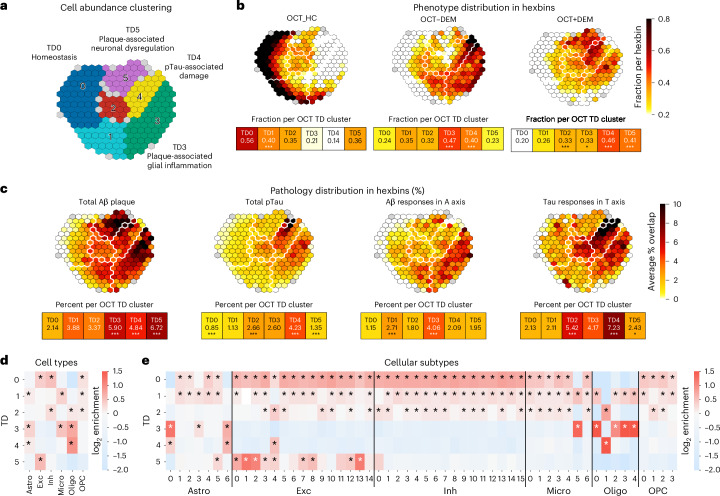
Six distinct TDs characterize the octogenarian AD brain. **a**, Clustering of Visium spots into TDs from cortical layers 3–5 in the OCT cohort based on deconvoluted cell type abundance. UMAP dimensionality reduction followed by Leiden clustering identifies six distinct TDs (OCT-TD0 to OCT-TD5). The UMAP is shown as a hexbin plot (with, on average, 162 Visium spots per hexagon, IQR: 84−239) for visualization. Hexbins with five or fewer Visium spots are grayed out. **b**, Relative representation of phenotypic groups across the six TDs. The fraction of Visium spots from OCT_HC, OCT − DEM and OCT + DEM per hexbin is visualized by color intensity. Below, heatmaps display the average contribution of each group per TD. Enrichment for each cluster was determined by comparing the distribution of TD values within that cluster to those of all other clusters using one-sided Mann−Whitney *U*-tests followed by Bonferroni correction for multiple comparisons. Significance levels are denoted as follows: *P_adj_ < 0.05, **P_adj_ < 0.01, ***P_adj_ < 0.001. **c**, Pathology burden across TDs. For each hexbin, the mean percentage of Visium spot area overlapping with pathology is shown. The first two panels display overlap with total Aβ (4G8) and pTau (AT8) signal, respectively. The right panels depict the two spatial axes used throughout the study: the A axis, defined as Aβ overlap in spots lacking pTau signal (pTau = 0%), and the T axis, defined as pTau overlap restricted to spots with detectable Aβ (Aβ > 0%). Heatmaps summarize mean pathology overlap per TD. Statistical testing was performed as in **b**. **d**,**e**, Relative enrichment of major cell types (**d**) and cellular subtypes (**e**) across the TDs. Cell type abundances were estimated using cell2location and used to quantify enrichment within each TD. For every TD–cell type pair, enrichment was calculated as log_2_(observed / expected), where expected values reflect the abundance if the cells would be randomly distributed over TDs. One-sided *P* values for overenrichment were derived from standardized residuals and corrected for multiple testing using the Bonferroni method. An asterisk indicates *P*_adj_ < 0.001. See Supplementary Fig. 2 for hexbin plots per cell type and subtype. Astro, astrocytes; Exc, excitatory neurons; Inh, inhibitory neurons; Micro, microglia; Oligo, oligodendrocytes; OPC, oligodendrocyte precursor cells.

**Extended Data Fig. 2:**
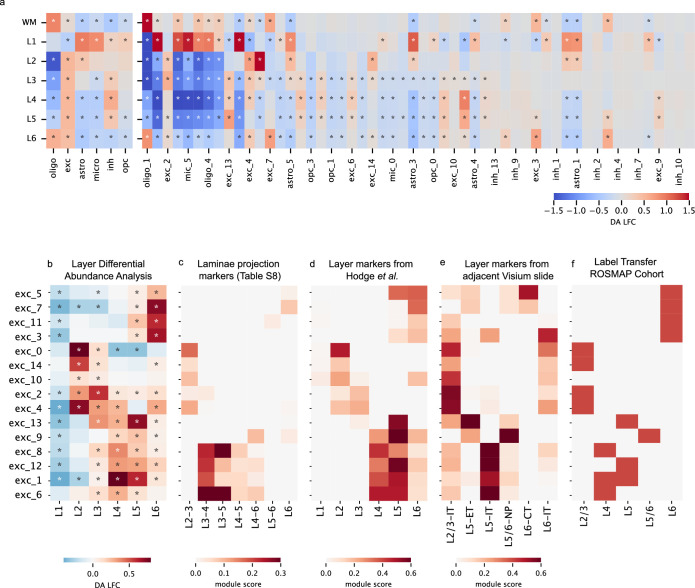
Cell deconvolution and in silico validation of cortical layer mapping. (**a**) **Cell type abundance across cortical layers inferred using** Cell2location. Abundance is shown as log-fold change of that layer relative to the mean across all layers. Color scale denotes enrichment (red) or depletion (blue). Significance of FDR < 0.05 is indicated by an asterisk. (**b**) Log-fold changes in excitatory neuron subtype abundance across cortical layers. Differential abundance was computed using Cell2location-inferred cell estimates. Red indicates enrichment, blue indicates depletion, significant differences are marked with an asterisk (FDR < 0.05). (**c-f**) In silico validation of layer-specific localization using independent marker sets. Heatmaps show average module scores per excitatory cluster using (**c**) laminar projection markers from the Xenium panel (Table S8), (**d**) layer-specific markers from ref. ^79^. (**e**) Visium-derived marker sets (Table S8). Across panels **c**-**e**, consistent patterns confirm layer specificity (for example Exc13 consistently shows enrichment for L5-ET markers across all data sets, validating its localization to cortical layer 5 as inferred in panel **b**). (**f**) Cross -validation with the **ROSMAP cohort**. scANVI label transfer shows correspondence between excitatory neuronal subtypes from the OCT cohort (y-axis) and layer-specific excitatory subtypes from the ROSMAP^20^ human brain cohort (x-axis). Standardized residuals from chi-square tests indicate significant overlaps (>2, shown in orange). Non-significant associations are shown in gray. (See also Table S5).

To deconvolve cell composition within Visium spots, we applied cell2location^48^ confirming layer-specific enrichment of excitatory neurons (Extended Data Fig. 2 and Supplementary Data 1). Harmony^49^ was then used to correct for layer effects, followed by unbiased Leiden clustering that identified six distinct clusters of Visium spots, termed tissue domains (TDs: OCT-TD0 to OCT-TD5; Fig. 2a), each representing a microenvironment with a characteristic cellular composition (Fig. 2b–e). Pathological relevance was inferred a posteriori by correlating TD composition with the OCT cohorts (Fig. 2b) and local Aβ and pTau burden (Fig. 2c) rather than used as input for clustering.

**Fig. 3:**
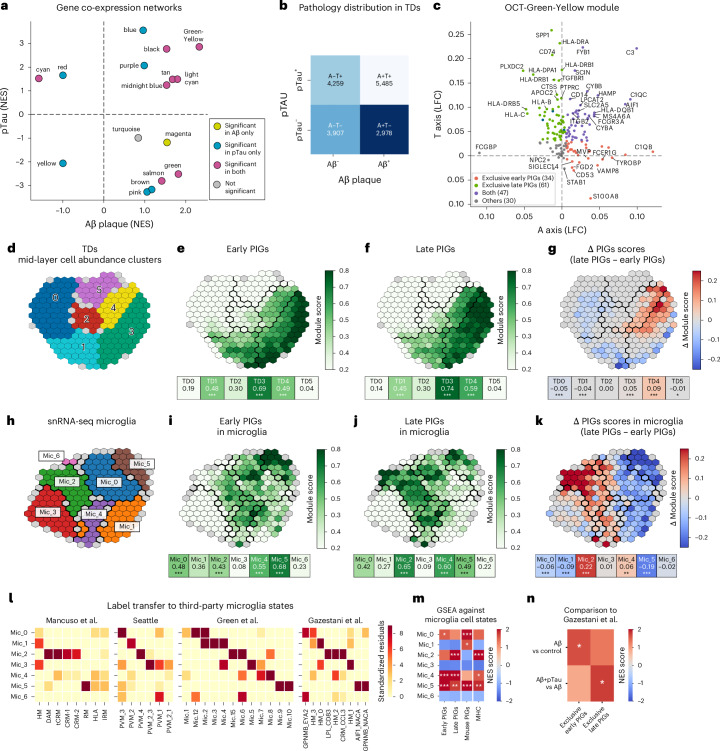
Early and late Aβ plaque-induced gene programs in octogenarian brains. **a**, Co-expression module response to Aβ and pTau pathology. WGCNA of 41,943 Visium spots spanning cortical layers 1−6 from 24 OCT brains identified 16 gene modules (Supplementary Data 2 and Extended Data Fig. 5). The scatter plot shows the NES for each module based on GSEA against Aβ plaque load (*x* axis) and pTau load (*y* axis). Modules enriched along both axes are shown in magenta, those enriched along the Aβ axis in yellow, those enriched along the pTau axis in cyan and non-significant modules in gray. **b**, Sample sizes used to define Aβ (A axis) and pTau (T axis) responses. Visium spot counts from cortical layers 3−5 only in OCT + DEM and OCT − DEM brains are stratified by the presence or absence of Aβ and pTau pathology. **c**, Gene-level differential expression of OCT-Green-Yellow module genes along the A axis and the T axis. Each point represents one gene from the OCT-Green-Yellow module positioned according to the log fold change of the differential expression in the A axis (*x* axis, differential expression against Aβ in absence of pTau in the A+T− and A−T− Visium spots) and the T axis (*y* axis, differential expression against pTau in presence of Aβ in the A+T+ and A+T− Visium spots), computed from Visium spots in layers 3–5. The colors indicate early PIGs (leading-edge genes enriched along the A axis, red), late PIGs (leading-edge genes enriched along the T axis, green), shared PIGs (genes contributing to both enrichments, purple) and remaining module genes (gray). **d**−**g**, Spatial distribution of early and late PIGs across OCT TDs. **d**, TDs identified from Visium spots in cortical layers 3–5 based on deconvoluted cell type abundance (identical to Fig. 2a, reproduced for easy reference). **e**,**f**, Hexbin plots show *z*-normalized module scores for early PIGs (**e**) and late PIGs (**f**) across layers 3–5. Heatmaps below each panel summarize mean module scores per TD. Statistical significance was assessed using Bonferroni-corrected one-sided Mann−Whitney *U*-tests (*P_adj_ < 0.05, **P_adj_ < 0.01, ***P_adj_ < 0.001) comparing the distribution of early PIG or late PIG scores within each TD to the distribution across all other TDs. **g**, Relative early PIG versus late PIG activity in the Visium spots of cortical layers 3−5. The hexbin map shows the difference between *z*-normalized late PIG and early PIG module score (ΔPIGs = late − early). Red indicates late PIG dominance; blue indicates early PIG dominance. The heatmap summarizes mean ΔPIG scores per TD. Significance was evaluated using Bonferroni-corrected two-sided Mann−Whitney *U*-tests. **h**−**k**, Spatial distribution of early and late PIGs across microglia in OCT TDs. **h**, UMAP of OCT microglia from the snRNA-seq experiment in OCT cohort showing subtypes Mic_0 to Mic_6. Mic_5 represents the ribosomal biogenesis activation state aligned with early PIG responses to Aβ; Mic_2 is an inflammatory, antigen-presenting state aligned with late PIG programs associated with pTau pathology. **i**,**j**, Early PIG (**i**) and late PIG (**j**) module scores across microglial subtypes. Hexbin plots and heatmaps show *z*-normalized module scores for Mic_0 to Mic_6. Significance was assessed using Bonferroni-corrected one-sided Mann–Whitney *U*-tests (*P_adj_ < 0.05, **P_adj_ < 0.01, ***P_adj_ < 0.001), comparing the distribution of module scores for each subtype to all others. **k**, Relative early PIG versus late PIG activity across microglial subtypes. Hexbin plot and heatmap display ΔPIG scores (late − early). Red indicates dominance of late PIG expression; blue indicates dominance of early PIG expression. Significance was determined using Bonferroni-corrected two-sided Mann−Whitney *U*-tests (*P_adj_ < 0.05, **P_adj_ < 0.01, ***P_adj_ < 0.001) comparing ΔPIG distribution of each subtype to all others. **l**, Cross-cohort comparison of microglial states. scANVI label transfer mapped OCT microglial subtypes to reference datasets from human xenografts^30^, SEA-AD^34^, ROSMAP^20^ and Gazestani et al.^18^. Heatmap shows standardized residuals from *χ*^2^ tests; values >2 indicate significant overrepresentation of a reference state within an OCT-defined subtype. **m**, GSEA of early PIGs, late PIGs and MHC-related gene sets across OCT microglial states. Heatmap shows NESs derived from cell-type-specific differential expression. Significance is indicated as *P_adj_ < 0.05, **P_adj_ < 0.01 and ***P_adj_ < 0.001. **n**, GSEA of early and late PIG signatures in microglia from Aβ^+^ and Aβ^+^tau^+^ cortical biopsies^18^. Heatmap shows NES values based on biopsy-level differential expression (significance as in **m**).

**Extended Data Fig. 3:**
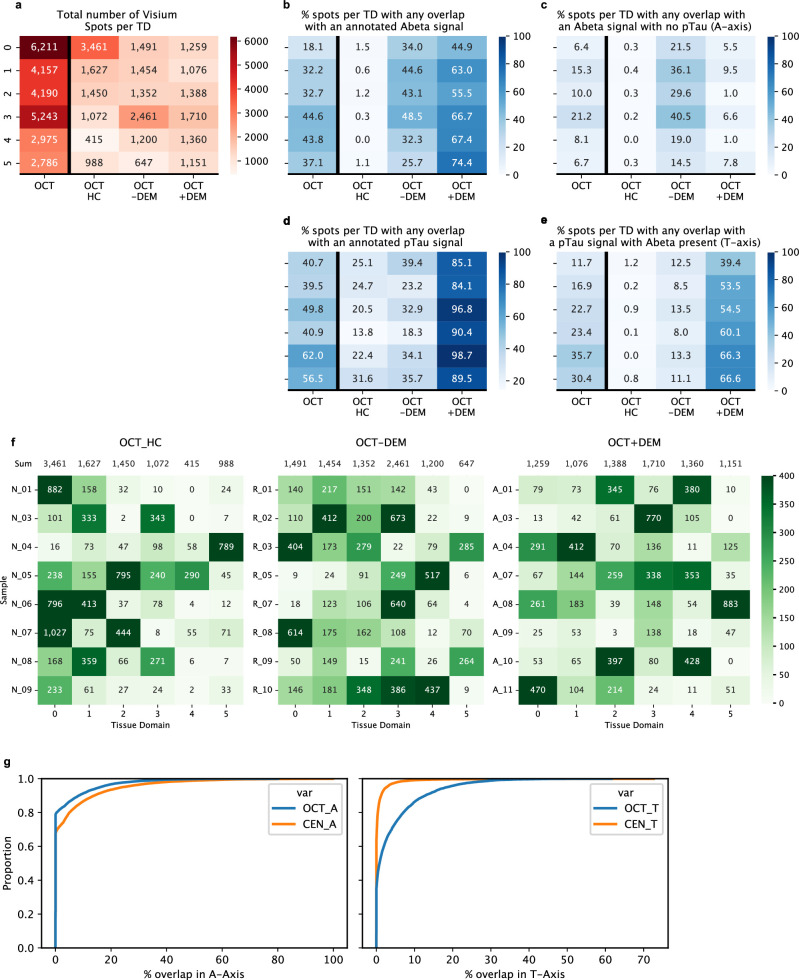
Prevalence of Aβ and pTau pathology across Tissue Domains and phenotypic groups. (**a**) Total Visium spot counts per Tissue Domain (TD) and phenotypic group (OCT_HC, OCT–DEM, OCT + DEM). (**b**) Proportion of Visium spots with any overlap with an annotated Aβ signal (4G8) across TDs and phenotypic groups. (**c**) as (**b**), but for Abeta annotated spots with no pTau (A-axis). (**d**) Proportion of Visium spots with overlap to annotated pTau (AT8). Together, these heatmaps illustrate the spatial heterogeneity of Aβ and pTau burden across microenvironmental states and diagnostic groups. (**e**) as (**d**), but for pTau overlapping spots with Abeta present (T-axis). (**f**) Total Visium spot counts per Tissue Domain (TD) for each individual sample. (**g**) Empirical cumulative distribution functions (ECDFs) of Visium spot overlap with Aβ-only pathology (A-axis) and Aβ+pTau pathology (T-axis) in OCT and CEN cohorts. Curves show the full distributions underlying Fig. 5i. Sample sizes: OCT_A n = 13,843; CEN_A n = 18,740; OCT_T n = 8,507; CEN_T n = 9,604.

TD0 and TD1 represent homeostatic environments with minimal pathology, comprising 56% of spots from healthy controls (OCT_HC) and containing a broad diversity of neuronal and glial subtypes. TD2 corresponds to a transitional environment where the earliest cellular responses to pathology appear; it is evenly represented across the OCT cohort (Extended Data Fig. 3) and enriched in the Exc_4 excitatory neuronal subtype and the Oligo_1 oligodendrocyte subtype (Fig. 2e and Supplementary Fig. 2). TD3 and TD5 were both strongly associated with Aβ plaques but differed in their cellular composition. TD3 was enriched in reactive glia and oligodendrocytes (Fig. 2d,e and Supplementary Data 1), consistent with an early gliotic and myelination-related response surrounding plaques. TD5, by contrast, was dominated by excitatory neurons and astrocytes, showing transcriptional enrichment for oxidative phosphorylation and mitochondrial metabolism pathways, indicating elevated energy metabolism rather than overt stress signaling. TD4 represented a distinct, pTau-dominated environment (often co-occurring with Aβ), enriched in DAM-like microglia, reactive astrocytes, Oligo_1 oligodendrocytes and Exc_4 neurons, partly overlapping with vulnerable neuronal populations described in the SEA-AD atlas^34^ (Supplementary Table 5). Among the microglial states identified in our snRNA-seq dataset (Supplementary Fig. 1i), Mic_1 and Mic_3 represent homeostatic states, Mic_5 reflects a ribosomal biogenesis activation state and Mic_2 corresponds to a disease-associated inflammatory state; full characterization of these states is presented in the microglial transition section below (Fig. 3l, Supplementary Fig. 2e and Supplementary Table 7). All TDs, ranging from low to high pathology, were observed across each of the three cohorts (Extended Data Fig. 3).

Together, these TDs outline a spatially ordered continuum from homeostasis (TD0−TD1) through early adaptive responses (TD2−TD3) to advanced pathology (TD4−TD5) (Fig. 2a). The sequence indicates a gradual transformation of the cortical microenvironment: from normal architecture to Aβ-associated gliosis and metabolic activation and, ultimately, to pTau-dominated degeneration. This TD progression reveals a spatially localized inflection point between TD3 and TD4 where glial programs shift from Aβ-driven to pTau-associated states. These transitions are consistent with a pathological interface between amyloid and tau, providing a spatial framework to interrogate mechanisms that influence whether local immune activation leads to containment or progression of disease.

### Neuroinflammatory signatures defined by co-expression modules

**Extended Data Fig. 5:**
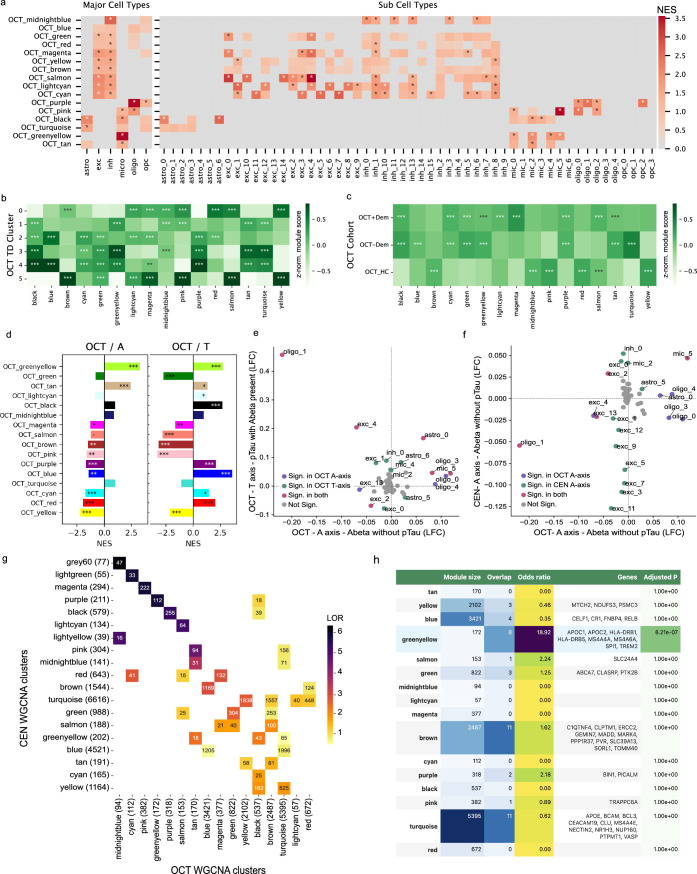
Gene network architecture and pathology-associated cell state changes in octogenarian brains. (**a**) **Cell type enrichment of OCT WGCNA modules**. The heatmap shows enrichment of WGCNA module genes (y-axis) in ranked gene lists derived from differential expression analysis of major cell types and their subtypes. For major cell types, genes were ranked by log fold changes (logFC) from differential expression of that cell type versus all others. For subtypes, rankings were based on logFC of each subtype versus all other subtypes within the same cell type. Only positively enriched modules (NES > 0) are shown, negative NES values are shown in grey. Statistically significant enrichments (FDR < 0.05) are marked with an asterisk (*). Methods provide full details of the enrichment analysis. (**b**) **Enrichment of WGCNA modules across Tissue Domains**. Heatmap shows mean z-normalized module score across the six OCT TD. Significance was assessed using a Bonferroni corrected one-sided Man-Whitney U tests comparing scores in each Tissue Domain versus all others. * (p < 0.05), ** (p < 0.01), and *** (p < 0.001). (**c**) **Enrichment of WGCNA modules by clinical phenotype**. Relative enrichment of each WGCNA module (x-axis) across phenotypic groups (y-axis): cognitively healthy controls (HC), pathology without dementia (–DEM), and with dementia (+DEM). Analysis as in panel (**b**). (**d**) **Enrichment of WGCNA modules along Aβ- and Tau associated axes**. GSEA of OCT WGCNA modules using differential expression ranks along the A-axis (Aβ^+^/pTau^−^) and T-axis (Aβ^+^/pTau^+^). NES values are shown per module; significance indicated as ***P < 0.001, **P < 0.01, *P < 0.05. (**e**) **Shifts in cellular abundance along the A- and T-axes**. We performed Differential Abundance Analysis (DAA, see Methods) for all sub cell-types against the A-axis (x-axis) and T axis (y-axis). Color indicates significance (FDR < 0.05): red: significant on both axes; purple: A-axis only; green: T-axis only. (**f**) **Cross-cohort comparison of cell state enrichment along A-axes**. Same analysis as (**e**) comparing NES values for OCT A-axis (x-axis) and CEN A-axis (y-axis) highlighting conserved and divergent responses to Aβ pathology across cohorts. Colors indicate significance in both axes (red), OCT-A only (purple) and CEN_A only (green). (**g**) **Gene set overlaps between WGCNA modules from the Octogenarian and Centenarian cohorts**. Heatmap shows the log-odds ratio from Fisher’s Exact Test for overlap between modules. Only significantly overlapping pairs (Bonferroni-adjusted P < 0.05) are colored. Numbers indicate overlapping genes. (**h**) **Overlap between OCT WGCNA modules and AD GWAS risk loci**. Table reports log-odds ratios and adjusted P-values from Fisher’s Exact Test assessing overlap between each WGCNA module and 59 AD GWAS risk genes (MAGMA^51^ analysis using summary statistics^24^. Overlapping genes are listed per module.

Weighted gene co-expression network analysis (WGCNA)^50^ on 43,169 gray matter Visium spots identified 16 modules (Supplementary Tables 3 and 4 and Extended Data Fig. 5). Among these, the OCT-Green-Yellow module (172 genes) emerged as the dominant pathology-responsive program, showing the strongest and most consistent upregulation with increasing Aβ and pTau burden (Fig. 3a). Genome-wide association studies (GWASs)^24^ and mechanistic analyses^25^ have established microglia and immune pathways as major genetic determinants of AD. The OCT-Green-Yellow module was highly enriched for microglia markers (normalized enrichment score (NES) = 4.1; Extended Data Fig. 5a) and immune functions including leukocyte-mediated immunity (GO:0002443, adjusted *P* value (*P*_adj_) = 7.9 × 10^−29^), cytokine production (GO:0001819, *P*_adj_ =3.3 × 10^−20^), myeloid cell activation (GO:0002275, *P*_adj_ =7.1 × 10^−14^) and antigen presentation (GO:0019882, *P*_adj_ = 1.2 × 10^−21^) (Supplementary Table 4). Notably, the module contained numerous *TREM2*-related signaling genes (Supplementary Table 3) and showed strong overlap with the PIGs identified in mouse models (mouse PIGs; log odds ratio = 4.06, *P* = 2.2 × 10^−16^)^23^, a microglial and astroglial program induced by proximity to Aβ plaques, highlighting a conserved innate immune response to Aβ across species.

Integration with AD GWAS summary statistics^24^, aggregated using MAGMA^51^, revealed that OCT-Green-Yellow was the only module significantly enriched for AD risk genes (Fisher’s exact test, *P*_adj_ = 6 × 10^−7^), including *SPI1*, *APOC1*, *HLA-DRB1*, *MS4A6A*, *MS4A4A*, *HLA-DRB5*, *TREM2* and *APOC2* (Extended Data Fig. 5h). Spatially, OCT-Green-Yellow expression peaked in the Aβ-rich TD3 and extended into the pTau-associated TD4, positioning this module precisely at the Aβ−tau inflection point. Together, the spatial and genetic evidence indicates that OCT-Green-Yellow captures the core microglial program engaged as tissue microenvironments transition from Aβ deposition toward pTau accumulation.

### Dissecting early and late plaque-induced gene programs in AD

The cellular phase hypothesis of AD proposes that Aβ accumulation triggers a coordinated series of cellular responses that ultimately give rise to pTau pathology and neurodegeneration^8,52^. The OCT-Green-Yellow module lies at the center of this transition: it responds to both Aβ and pTau (Fig. 3a); overlaps strongly with mouse PIGs; is enriched for AD GWAS risk genes (Extended Data Fig. 5h); peaks in TD3−TD4 (Extended Data Fig. 5b); and is predominantly expressed in microglia (Extended Data Fig. 5a).

This prompted us to test whether the module reflects a single continuum or separable early and late immune programs marking successive stages of Aβ-associated and tau-associated activation. We focused again on cortical layers 3−5, which showed consistently the strongest convergence of Aβ plaques and pTau pathology across individuals (Fig. 1c). We stratified (Fig. 3b) 16,629 Visium spots from OCT + DEM (*n* = 7,963) and OCT − DEM (*n* = 8,666) into four pathologically defined groups (Fig. 3b): A+T+ (*n* = 5,485); A+T− (*n* = 2,978); A−T+ (*n* = 4,259); and A−T− (*n* = 3,907). Spots from different groups occur in different cohorts and individuals. This enabled two orthogonal comparisons (illustrated in Fig. 1e): the A axis captures Aβ-associated changes without pTau (A+T− versus A−T− (*n* = 6,885)), and the T axis captures changes on an Aβ background (A+T+ versus A+T− (*n* = 8,463)).

GSEA revealed strong enrichment of OCT-Green-Yellow along both axes (A axis: NES = 3.32, T axis: NES = 2.71, both false discovery rate (FDR) *P* ≈ 0.0; Fig. 3c and Extended Data Fig. 5d). To map the module structure, we plotted all module genes according to their signed differential expression statistics along the A axis and T axis contrasts and defined early, late and shared PIGs using the GSEA leading-edge subsets (Fig. 3c and Supplementary Tables 3 and 4): 81 early PIGs induced by Aβ alone, 108 late PIGs emerging with both Aβ and pTau present and 47 shared along both axes.

Early PIGs were enriched for complement (*C1QA*, *C1QB*, *C1QC*, *C3*) and TREM2−TYROBP signaling pathways (*SYK*, *FYB1*, *HCK*; *BLNK*; Fc receptors; SIGLEC receptors) with Gene Ontology terms linked to ERK signaling (GO:0070372), myeloid differentiation (GO:0002573), low-density lipoprotein (LDL) responses (GO:0071404) and chemotaxis (GO:0050920) (Supplementary Table 4).

The late PIGs were dominated by antigen presentation and immune activation genes (*HLA-DRA*, *HLA-DPA1*, *HLA-DPB1*, *HLA-DMA*, *HLA-DMB*, *HLA-DOA*, *HLA-C* and *CD74*) enriched for major histocompatibility complex class II (MHC-II) assembly (GO:0002399), macrophage activation (GO:0042116) and leukocyte adhesion (GO:1903039) (Supplementary Table 4).

Spatial mapping showed that early and late PIGs were highest in TD1, TD3 and TD4 (Fig. 3e–g), with a marked shift from early PIG to late PIG dominance between TD3 and TD4 (Fig. 3g). This sharp transition defines a microglial inflection point at the Aβ−tau interface: the early PIGs program is consistent with a protective or adaptive microglial response, whereas the late PIGs program is consistent with signal escalation toward antigen-presenting microglial states associated with tau-mediated neurodegeneration.

### Microglial state transitions parallel early and late PIG programs

To determine which microglial phenotypes implement the early PIG and late PIG responses, we integrated our snRNA-seq dataset with four human microglia reference atlases^20,30,34^ using scANVI^53^. This harmonized annotation identified seven microglial states (Mic_0 to Mic_6; Fig. 3h and Supplementary Fig. 1i), enabling direct mapping of early PIG and late PIG signatures onto discrete cellular phenotypes.

The early PIG program is predominantly expressed in Mic_5, a ribosomal biogenesis activation state, corresponding to the ribosomal microglia in Mancuso et al.^30^ and the ribosome/redox clusters reported by Green et al.^20^ (Fig. 3i,m). Early PIG enrichment in Mic_5 (Fig. 3i,m) was driven by complement and *TREM2* pathway genes such as *C1QB*, *APOC1*, *TYROBP* and *S100A8*. Spatially, Mic_5 was most frequent in TD1, TD3 and TD5, tissue environments enriched for Aβ plaques but with minimal pTau, consistent with early PIG biology (Fig. 2e).

Late PIG programs mapped most strongly to Mic_2 (Fig. 3j,m), an inflammatory, antigen-presenting phenotype that aligns with disease-associated microglia (DAM) and cytokine-responsive microglia (CRM), described by Mancuso et al.^30^, and with the PVM_4 state in the SEA-AD atlas^34^. Late PIG enrichment in Mic_2 included antigen presentation and inflammatory genes (*SPP1*, *CCL3*, *CH25H*, and *RGS1*) with Gene Ontology terms highlighting lipid metabolism (GO:0071222, *P*_adj_ = 2.0 × 10^−7^) and cytokine signaling (GO:1990868, *P*_adj_ = 2.7 × 10^−6^) (Fig. 3m and Supplementary Table 7). Although Mic_2 was most abundant in homeostatic tissue domains overall, it is the dominant phenotype in pTau-rich TD4, the environment that defines the Aβ–tau inflection point (Fig. 2e and Supplementary Fig. 2e).

Mic_1 and Mic_3 represented a homeostatic state corresponding to the homeostatic cluster in Mancuso et al.^30^, PVM_2 and PVM_2_3 in SEA-AD^34^ and surveilling microglia (Mic.2−5) in Green et al.^20^, providing the baseline from which early PIG activation emerges.

Together, these observations show that early PIG and late PIG modules correspond to distinct microglial activation states, forming a staged response from early Aβ-driven activation (Mic_5) to later antigen-presenting states associated with pTau. We next validated these programs using independent datasets and orthogonal spatial and histological methods.

### Orthogonal validation of early PIG and late PIG programs across microglial states and pathology

To validate our findings, we employed several independent approaches. First, we compared our early PIG and late PIG signatures with microglial states reported by Gazestani et al.^18^, who profiled cortical biopsies classified as no-pathology, Aβ-only or Aβ + pTau. Consistent with our Visium-derived signatures, the early PIG program was enriched in Aβ-only tissue, whereas the late PIG program was enriched in Aβ + pTau tissue (Fig. 3n). Moreover, Gazestani et al.’s LPL_CD83 microglial state that associated strongly with their late Aβ + pTau samples mapped closely to our late PIG-associated Mic_2 state (Fig. 3l,n).

**Fig. 4:**
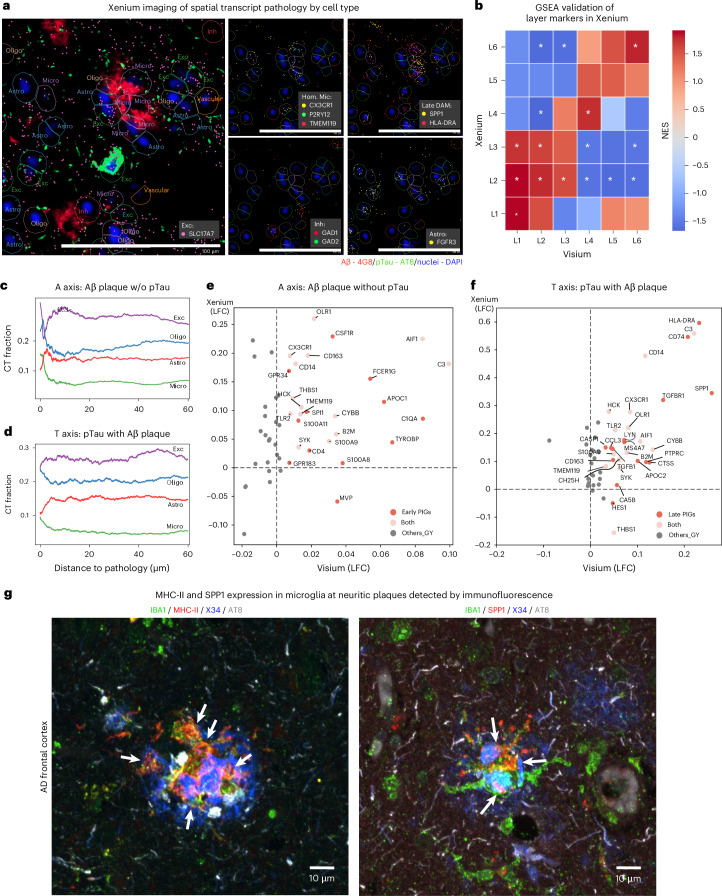
Xenium and immunofluorescence validation of early PIG and late PIG programs. **a**, Multiplexed Xenium imaging of spatial transcript−pathology relationships. All panels show the same cortical region from AD donor A_01 (Supplementary Table 1a) with high pTau pathology. The large panel (left) shows multiplexed Xenium imaging with cell type annotations overlaid with Aβ (4G8, red), pTau (AT8, green) and nuclei (DAPI, blue). *SLC17A7* marks the dense presence of excitatory neurons in this region, whose complex morphology is only partially captured by DAPI-based segmentation. The four smaller panels show the same region with pathology channels removed, enabling visualization of cell-type-specific transcripts: homeostatic microglia (Hom. Mic.; *CX3CR1*, P2RY12, *TMEM119*), late DAM (*SPP1*, *HLA-DRA*), inhibitory neurons (*GAD1*, *GAD2*) and astrocytes (*FGFR3*). Cell boundaries are color coded: microglia (purple), astrocytes (blue), oligodendrocytes (copper), excitatory neurons (green), inhibitory neurons (red) and vascular cells (orange). Scale bar, 100 µm. **b**, Validation of cortical layer-specific markers in Xenium identified excitatory neurons. Layer-specific marker genes identified from Visium spatial transcriptomics (Extended Data Fig. 2e and Supplementary Table 8) used as gene sets in a pre-ranked GSEA on layer-specific differential expression ranks performed on excitatory neurons as identified by the Xenium experiment. Heatmap confirms layer structure by showing NES enrichment across cortical layers (red, enrichment; blue, depletion). Asterisks indicate FDR < 0.05. **c**, Cell abundance changes relative to Aβ plaques (A axis). Xenium-derived proportions (as CT, cell type fractions) of major cell types are plotted as a function of distance (µm) from Aβ plaques computed using a rolling window of 5,000 cells (0−50 µm). Percent changes over 50 µm are shown in the legend. **d**, Cell abundance changes relative to pTau pathology (T axis). Cell type density profiles a function of distance from pTau pathology, analyzed as in **c**. **e**, Validation of early PIGs along the A axis. Scatter plot comparing log fold changes (LFCs) for OCT-Green-Yellow module genes in Visium (*x* axis) and Xenium (*y* axis). Xenium LFCs are computed from proximity to Aβ plaques (25-µm halo). Early PIG genes (A axis only) are shown in bold red; genes enriched on both axes are shown in light red; non-enriched genes are shown in gray. **f**, Validation of late PIGs along the T axis. Scatter plot comparing LFCs of OCT-Green-Yellow genes in Visium (*x* axis) and Xenium (*y* axis) as a function of proximity to pTau pathology. Gene coloring follows **e**. **g**, Immunofluorescence validation of late PIG markers at neuritic plaques. Representative images from immunostaining of frontal cortex from symptomatic patients with AD (AD_03 for MHC-II, red, left panel; AD_01 for SPP1, red, right panel) show co-labeling with microglial marker IBA1 (green), amyloid dye X34 (blue) and pTau marker AT8 (gray) (*n* = 3 slides per patient). Arrows denote IBA1^+^ microglia expressing MHC-II or SPP1 at neuritic plaques. Individual fluorescence channels are shown in Extended Data Fig. 6. Information on the donors used for staining is provided in Supplementary Table 1c. Astro, astrocytes; Exc, excitatory neurons; GY, Green-Yellow; Inh, inhibitory neurons; Micro, microglia; Oligo, oligodendrocytes; OPC, oligodendrocyte precursor cells.

Second, we applied the 10x Xenium platform, which provides hybridization-based spatial transcript localization at single-cell resolution. Xenium offers an orthogonal layer of validation by quantifying spatial patterns with a dedicated gene panel independent of Visium chemistry. We designed a custom 329-gene panel, including 50 OCT-Green-Yellow genes and canonical cell type markers (Supplementary Table 9). Xenium profiling was performed on 11 sections from five OCT + DEM and six OCT − DEM brains, with Aβ, pTau and NeuN immunostaining on the same slides (Fig. 4a). Pathology analysis followed the same pipeline as for Visium.

Using expanded DAPI-based segmentation (5-µm margin), we obtained 424,056 high-quality pseudocells. Although Xenium lacks the depth to resolve the full glial diversity captured by sn-RNAseq (Supplementary Fig. 1), the major cell types were clearly classified (Fig. 4a), and cortical layer markers confirmed data quality (Fig. 4b).

Transcriptional changes were then mapped along the A axis (Aβ^+^, pTau >25 µm away) and the T axis (pTau^+^, Aβ <25 µm away), where 25 µm approximates one Visium spot radius. Cell type distributions along these axes were analyzed by ordering all Xenium pseudocells according to their distance from the nearest Aβ or pTau signal and then calculating the local proportion of each cell type in a rolling window of 5,000 cells, which smooths local variability while preserving spatial trends (Fig. 4c,d). Microglia were enriched near pathology along both axes, consistent with our observation that microglia accumulate in pathological TDs (Fig. 2d) and with previous reports of progressive microglial recruitment to plaques^22^. Astroglia formed a core/shell pattern around pTau deposits with microglia in the core and oligodendrocytes at the periphery, which diverges somewhat from a previous report^54^. Excitatory neuron loss was most pronounced along the T axis, consistent with tau-associated neurotoxicity.

Expression of early PIGs (A axis) and late PIGs (T axis) correlated well between Visium and Xenium datasets (Fig. 4e,f) with particularly robust agreement for late PIG expression, reinforcing the link among pTau pathology, Mic_2 states and neurodegeneration.

**Extended Data Fig. 6:**
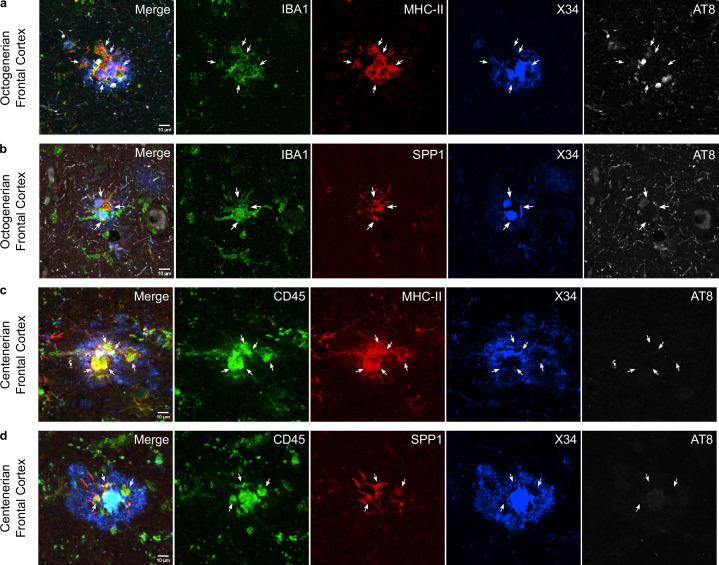
Immunofluorescence staining of MHC-II and SPP1 in microglia at neuritic plaques. Single-channel immunofluorescence images showing representative stainings for MHC-II (panels **a** and **c**) and SPP1 (panels **b** and **d**), acquired with a 20X objective on a Nikon A1R laser-scanning confocal system mounted on a Nikon Eclipse Ti inverted microscope. Images were obtained from frontal cortex sections of two AD donors (panels **a** and **b**, AD_03 for MHC-II, AD_01 for SPP1) and one centenarian donor (C_08). Donor details are provided in Table S1. In AD tissue, microglia were labeled with IBA1 (green) together with MHC-II (**a**, red) or SPP1 (**b**, red), amyloid with X34 (blue), and pTau with AT8 (grey); the corresponding multichannel images were also shown in main text Fig. 4g. Panels **c** and **d** display the results for the centenarian donor, CD45 (green) was used instead of IBA1 (co-labeling shown in Fig. 5j) together with MHC-II (**c**, red) or SPP1 (**d**, red), amyloid with X34 (blue), and pTau with AT8 (grey). Arrows highlight IBA1^+^ or CD45^+^ microglia expressing MHC-II or SPP1.

As a final validation, we performed limited immunofluorescence staining on two independent formalin-fixed paraffin-embedded (FFPE) AD samples (Supplementary Table 1c) using antibodies against MHC-II and SPP1. Both markers were strongly expressed in microglia adjacent to pTau^+^ plaques (Fig. 4g and Extended Data Fig. 6), indicating the spatial localization of late PIG states at sites of tau pathology.

### Neuroinflammation and resilience mechanisms in centenarians

The CEN cohort represents a highly intriguing collection of individuals who age exceptionally well and display remarkable resilience to dementia. To evaluate whether their spatial transcriptomic landscapes differ from those of the OCT cohort, we generated 61,739 Visium spots from 20 individuals processed identically to the OCT cohort (Fig. 1g). Clustering based on combined Aβ-associated and pTau-associated measures showed that CEN brains grouped more closely with OCT − DEM brains than with OCT + DEM brains (Extended Data Fig. 1a), consistent with preserved cognition despite substantial Aβ accumulation.

**Fig. 5:**
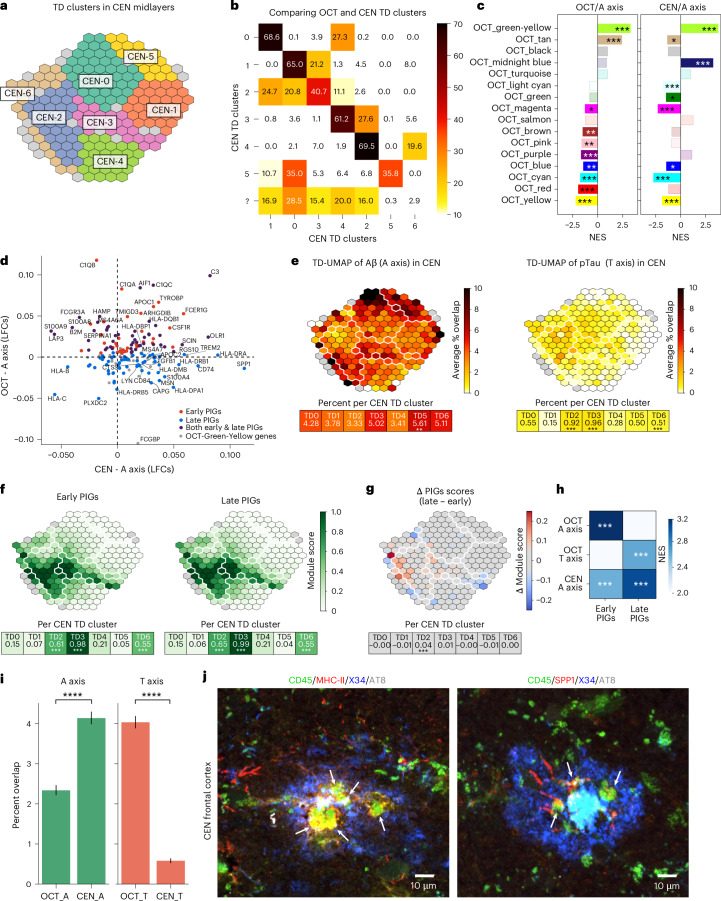
Cross-cohort validation of AD-related neuroinflammation in the CEN cohort. **a**, TDs in the CEN cohort. Visium spots from cortical layers 3–5 were clustered using cell2location-inferred cell type abundances followed by Leiden clustering, yielding seven CEN TDs (CEN-TD0 to CEN-TD6). The UMAP is visualized as a hexbin plot (average of 122 Visium spots per bin); bins with five or fewer Visium spots are grayed out. **b**, Cross-cohort mapping of TDs. Heatmap shows the proportion of CEN TDs (*x* axis) and OCT TDs (*y* axis) using an MLP classifier. Each cell shows the percentage of CEN Visium spots mapped to a given OCT TD; color intensity corresponds to mapping strength. **c**, Enrichment of OCT gene co-expression modules along the CEN A axis. GSEA of OCT WGCNA modules against differential expression ranks along the CEN A axis (Aβ^+^pTau−). NES values are shown. *P_adj_ < 0.05, **P_adj_ < 0.01, ***P_adj_ < 0.001. **d**, Conservation of early PIG and late PIG expression along the A axis. Scatter plot comparing log fold changes (LFCs) for OCT-Green-Yellow module genes along the A axis in OCT (*y* axis) versus CEN (*x* axis). Early PIGs are shown in red; late PIGs are shown in blue; shared early and late PIG genes are shown in purple; and the remaining, non-enriched, OCT-Green-Yellow genes are shown in gray. **e**, Spatial distribution of Aβ and pTau pathology in CEN. Hexbin plots show mean Visium spot overlaps with Aβ (left) and pTau (right). Heatmaps summarize mean overlap per TD. Statistical testing between TD follows Fig. 2b using Bonferroni-corrected one-sided Mann−Whitney *U*-tests: *P_adj_ < 0.05, **P_adj_ < 0.01, ***P_adj_ < 0.001. **f**, Early PIG and late PIG module scores respond similarly across CEN TDs. Visualized are *z*-normalized module scores for early PIGs (left) and late PIGs (right) across CEN cortical layers 3−5. Below, heatmaps summarize average module scores per CEN TD cluster (as opposed to OCT TDs in previous plots). Significance was assessed using Bonferroni-corrected one-sided Mann−Whitney *U*-tests (*P_adj_ < 0.05, **P_adj_ < 0.01, ***P_adj_ < 0.001). **g**, Relative expression of late PIGs versus early PIGs in CEN. Hexbin plot and heatmap show ΔPIG scores (late − early) per Visium spot (red, late PIG dominance; blue, early PIG dominance), confirming that early and late PIGs co-distribute in the CEN TDs. Significance was assessed using Bonferroni-corrected two-sided Mann−Whitney *U*-tests. **h**, GSEA of early PIGs and late PIGs across cohorts and pathology axes. Heatmap of NES values for non-overlapping early PIG and late PIG gene sets tested against differential expression ranks from OCT (A and T axes) and CEN (A axis). ***FDR < 0.001. **i**, Global pathology burden in OCT versus CEN. Bar plots show mean Visium spot overlap with Aβ-only pathology (A axis) and Aβ + pTau pathology (T axis) in both cohorts; error bars indicate 95% confidence intervals. Sample sizes: OCT_A *n* = 13,843; CEN_A *n* = 18,740; OCT_T *n* = 8,507; CEN_T *n* = 9,604. Significance was assessed using two-sided Mann−Whitney *U*-tests; ****P < 0.0001. Full data distributions are shown as ECDFs in Extended Data Fig. 3g. **j**, Immunofluorescence validation of late PIG markers in centenarians. Representative images are shown from immunostaining for MHC-II (left, red) and SPP1 (right, red), together with CD45 (green), amyloid dye X34 (blue) and AT8 (gray) in frontal cortex (layers 3−5) from centenarian donor C_08 (*n* = 3) (Supplementary Table 1a). Arrows mark CD45^+^ microglia expressing MHC-II or SPP1. Individual channels are shown in Extended Data Fig. 6. ECDF, empirical cumulative distribution function.

To compare spatial microenvironments, we focused on 26,400 CEN Visium spots from cortical layers 3−5 and estimated cell type proportions using cell2location with OCT snRNA-seq as reference. Unsupervised clustering revealed seven CEN TDs, again defined as Visium spot clusters (Fig. 5a). A multilayer perceptron (MLP) classifier trained on OCT Visium spots (93% accuracy) assigned 80% of CEN Visium spots to OCT-derived TDs (Fig. 5b), indicating broad conservation of microenvironmental states. The remaining 20% likely represent centenarian-specific niches absent from the younger OCT brains. Projection of Aβ and pTau pathology onto the CEN TD uniform manifold approximation and projection (UMAP) showed that Aβ and very low levels of pTau were distributed across all CEN TDs (Fig. 5e). As expected, Aβ burden (A axis) was higher in CEN than in OCT, whereas pTau (T axis) was minimal (Fig. 5i and Extended Data Fig. 3g).

WGCNA on 39,358 gray matter spots identified 20 co-expression modules (Supplementary Data 2), most of which were strongly preserved across cohorts (Extended Data Fig. 5g). The neuroinflammatory OCT-Green-Yellow module was robustly conserved (named CEN-Purple, log odds ratio = 5.76, *P* = 2.2 × 10^−16^), confirming persistent microglial activation despite limited pTau pathology. Given the low abundance of pTau in centenarians, analyses were restricted to the A axis (amyloid-associated pathology) and did not include the T axis. Along the A axis, CEN-Purple was the most strongly enriched module (NES = 3.74, *P*_adj_ ≈ 0; Fig. 5c), mirroring the OCT-Green-Yellow module in the octogenarians.

Two modules differed markedly between cohorts. OCT-Tan, upregulated along the A axis in OCT, was downregulated in CEN and enriched for wound healing (GO:0042060, *P*_adj_ = 7.8 × 10^−8^) and angiogenesis (GO:0045765, *P*_adj_ = 8 × 10^−7^) (Supplementary Table 4). Conversely, OCT-Midnight Blue was strongly enriched in CEN and associated with neuropeptide signaling (GO:0007218, *P*_adj_ = 3 × 10^−6^) and interneuron migration (GO:1904936, *P*_adj_ = 5 × 10^−6^).

### Late PIGs are induced by Aβ in centenarians despite minimal tau burden

Direct comparison of the OCT and CEN axes (Fig. 5d) revealed that many early PIGs remained significantly upregulated in CEN, including complement (*C1QC* and *C3*) and *TREM2* pathway (*TYROBP*, *SYK*, *HCK*, *FCER1G*, *FCGR1A*) components. Unexpectedly, late PIGs, associated with pTau pathology in OCT, were apparently induced by Aβ alone in CEN. These included antigen presentation genes such as *HLA-DRA*, *HLA-DRB1*, *HLA-DPB1*, *HLA-DMB*, *HLA-DPA1*, *CD74*, *CTSS* and *IFI30*.

Notably, *SPP1* (osteopontin), which increased along the T axis in OCT, was instead induced along the A axis in CEN (Fig. 5d), suggesting an altered regulatory context. GSEA restricted to uniquely assigned early PIGs and late PIGs confirmed that late PIGs were enriched in CEN in response to Aβ alone, despite the near absence of detectable pTau pathology (Fig. 5h).

Immunofluorescence staining in a CEN sample (C_08 donor information in Supplementary Table 1a) further validated expression of SPP1 and MHC-II in microglia adjacent to pTau^−^ plaques (Fig. 5j), showing that late PIG programs can be engaged independently of tau.

In summary, CEN brains display a continuum of TDs from low inflammatory states (TD0 and TD1) to more activated environments (CEN-TD2 to CEN-TD6) that broadly mirror the OCT trajectory. Both early PIG and late PIG programs are activated in centenarians despite minimal pTau pathology. Thus, centenarian resilience reflects not reduced microglial activation per se but an inflammatory trajectory that remains uncoupled from downstream tau pathology.

## Discussion

This study dissects the cellular and molecular trajectories of AD across two informative human cohorts: octogenarians (OCT) and centenarians (CEN). All centenarians were cognitively intact at enrollment, although several later developed mild decline, providing a rare window into resilience mechanisms at the extreme end of the human lifespan. By integrating spatial transcriptomics, snRNA-seq and in situ hybridization, we identified two spatial/pathological inflection points: the initial microglial response to Aβ plaques and the subsequent transition to tau-associated neurodegeneration. These inflection points capture a pseudotemporal sequence inferred from spatially heterogeneity within the same tissue.

Aβ deposition was widespread in both cohorts, whereas extensive pTau pathology was restricted to OCT individuals living with dementia, particularly in layers 3−5. Hierarchical clustering of pathology metrics (Extended Data Fig. 1a) was for descriptive purposes and showed that centenarians grouped more closely with OCT − DEM than with OCT + DEM or healthy controls, underscoring that resilience is not marked by a muted immune response but, rather, by how inflammatory trajectories evolve. OCT-derived WGCNA modules provided a shared framework for cross-cohort comparison, although centenarians may also deploy resilience-specific programs not fully captured by OCT modules. Larger cohorts will be required to define these pathways systematically. In this context, the OCT-Tan (wound healing and angiogenesis) and OCT-Midnight Blue (neuropeptide signaling and interneuron migration) modules may offer initial clues.

We identified six TDs across a spatial pathological continuum, with TD3 and TD4 marking the critical transition from Aβ-associated inflammation to pTau-associated degeneration. This shift coincided with a microglial change from early complement and *TREM2*-enriched activation states (early PIGs) to later antigen-presenting phenotypes (late PIGs). Two resilience strategies emerged. Octogenarians without dementia mounted an early PIG response but did not transition to the antigen-presenting late PIG state. In centenarians, late PIG programs were engaged despite minimal tau pathology. This uncoupling demonstrates that late PIG activation is not intrinsically pathogenic but that its downstream consequences depend on the surrounding cellular and pathological context. Spatial transcriptomics and immunofluorescence staining (Fig. 5j) confirmed that HLA-DR^+^ and SPP1^+^ microglia populate Aβ-rich but pTau-poor regions, supporting this dissociation. Whether this altered trajectory reflects genetic, immune or aging-related factors in centenarians remains to be determined.

Although late PIGs show an antigen-presenting profile, they overlapped only partially with previously described HLA-enriched states, illustrating the modular and context-dependent nature of human microglial activation. Early PIG activity localized to Aβ plaques, whereas late PIGs marked regions with early tau pathology, consistent with a staged progression from innate activation to antigen presentation.

*SPP1* (osteopontin) illustrates this context dependence. In octogenarians, osteopontin promotes macrophage activation and was induced at advanced plaque stages, consistent with roles in synaptic remodeling and chronic inflammation^55^. In centenarians, by contrast, *SPP1* induction occurred earlier along the A axis (Fig. 5d), before extensive pTau accumulation. This pattern suggests that *SPP1* is not inherently detrimental but exerts different effects depending on timing and pathological context. Notably, SPP1 upregulation has also been observed after anti-Aβ immunotherapy^56^, where it enhances Aβ plaque removal without synaptic loss.

More broadly, our findings support a model in which persistent Aβ exposure reshapes microglial and astrocytic states and enables tau pathology. Microglia emerge as key nodes governing whether immune activation remains contained or progresses toward an antigen-presenting state associated with neurodegeneration. These observations align with reports that genetically protected centenarians exhibit microglia more responsive to early neuropathological changes^57,58^.

In conclusion, resilience in AD appears defined not by the absence of pathology but, rather, by the capacity to modulate its cellular consequences. Cognitive resilience may arise by restraining the transition from early to late PIG states or by uncoupling late microglial activation from tau aggregation. Therapeutic strategies may aim to maintain early microglial functions supporting Aβ clearance and synaptic maintenance while preventing chronic antigen-presenting activation associated with tau pathology. The late PIG state is not inherently pathogenic but becomes harmful under specific pathological configurations, positioning the Aβ−tau inflection point as a promising locus for therapeutic modulation. SPP1 represents one possible regulatory node in this process^56^, and approaches that modulate microglial state transitions, including anti-amyloid immunotherapies or agents targeting *TREM2* or *CSF1R*, may benefit from engagement before the inflammatory inflection point.

### Limitations

Several limitations should be acknowledged. First, this study is cross-sectional and correlative, and spatial heterogeneity provides only a pseudotemporal approximation; causality cannot be inferred. Second, the CEN cohort is complementary rather than directly comparable to OCT, and differences in age, genetics and survival may influence microglial states. Third, although six TDs were robust across donors, batch effects and interindividual variability cannot be fully excluded despite Harmony correction and shared WGCNA modules. Fourth, rare co-pathologies such as TDP43 or vascular contributions were not systematically quantified—as donor-level annotations limit statistical power—and may influence regional transcriptional states. Fifth, although late PIG activation shows an antigen-presenting signature, we did not detect lymphocyte infiltration, and functional interactions with adaptive immunity remain unresolved. Finally, larger cohorts and longitudinal sampling will be required to generalize resilience-associated pathways.

## Methods

### Postmortem human brain tissues

We selected a total of 56 individuals from the NBB (Netherlands Institute for Neuroscience) or from the Dutch 100-plus Study^45^, all with an RNA integrity number (RIN) above 6.8. All donors provided a written informed consent for brain autopsy and research use of material and clinical data following the guidelines from the ethics committee of the VU University Medical Center and the KU Leuven ethics committee (study number S63259). Autopsy procedures were approved by the medical ethics committee of the VU University Medical Center. The study included cognitive assessments (Clinical Dementia Rating (CDR), Reisberg scale or MMSE), neuropathological evaluations, *APOE* genotyping and bulk tissue transcriptomic profiling^9^. Detailed clinicopathological information is provided in Supplementary Table 1.

We selected eight controls (OCT_HC) with no/minimal AD pathology and 16 individuals with mild-to-severe Aβ and tangle deposition (OCT + DEM and OCT − DEM) (Supplementary Table 1). OCT_HC and OCT − DEM individuals were cognitively normal, whereas OCT + DEM had cognitive impairment. Donors with non-AD psychiatric or neurological disease were excluded. A second cohort (CEN) from the 100-plus Study comprised centenarians with mild-to-severe Aβ but minimal tangle pathology and a cognitive spectrum from normal to impaired, as assessed by MMSE. Cohorts were balanced for sex and age (Supplementary Table 1). Frozen superior frontal gyrus cylinders (8-mm diameter, covering all six cortical layers and white matter) were collected via biopsy punch (Ted Pella, 15111-80), shipped on dry ice to the De Strooper laboratory in KU Leuven and stored at −70 °C. Cohorts were balanced for sex; however, analyses were not sex stratified due to limited subgroup sizes, as the aim was to identify shared pathology-associated cellular trajectories.

### Pathological features and scoring

Aβ and pTau pathology were assessed using NBB-provided neuropathological scores (Thal amyloid phase, Braak stages for Lewy bodies and neurofibrillary tangles, CERAD neuritic plaque scores and ABC scores)^60^ (Extended Data Fig. 1a and Supplementary Table 1). Cored plaques were defined as cortical amyloid plaques with a compact central core and diffuse Aβ halo, and diffuse plaques were sharply delineated extracellular Aβ aggregates without an amyloid core^59^. All other plaque types with ill-defined borders or linked to specific anatomical areas such as subpial or white matter Aβ aggregates^59^ were considered as unclassified plaques in the context of this study. Aβ plaque density was significantly higher in OCT + DEM, OCT − DEM and CEN compared to OCT_HC (Fig. 1d), with no significant differences among the three pathology groups, indicating similar frontal lobe plaque loads. By contrast, OCT + DEM cases had significantly more neuritic plaques than OCT − DEM, OCT_HC and CEN (Extended Data Fig. 1a). Because Thal and Braak scores reflect brain-wide pathological distribution rather than individual Visium spot loads, Aβ and pTau abundance and morphology were determined per Visium spot via immunohistochemistry on the same tissue sections used for spatial transcriptomics (Fig. 1e).

### Tau morphology across experimental groups

pTau pathology was assessed using an AT8 antibody against pTau (Ser202/Thr205; Pierce, 1/100)^61,62^ and revealed large qualitative differences across experimental groups. In OCT + DEM cases, pTau regions of interest (ROIs) exhibited neuronal threads, neurofibrillary tangles and dystrophic neurites strongly associated with Aβ plaques. Conversely, pTau in OCT_HC brains was diffusely distributed and predominantly localized to the soma. OCT − DEM and CEN brains showed pTau morphologies resembling those in OCT_HC, suggesting less advanced pathology. Age-related tau astrogliopathy (ARTAG), often observed in the white matter of cognitively healthy seniors, was also detected. Differentially expressed genes associated with pTau and neuritic plaques showed significant correlations within the same experimental group (for example, *r* = 0.5 for OCT + DEM) but differed substantially between groups (for example, *r* = 0.1 between OCT + DEM and OCT − DEM). This suggests that variations in pTau types contribute to differences in gene expression profiles across experimental groups.

### Tissue collection

For Visium Spatial Gene Expression, frozen brain blocks were cryosectioned at 10 µm (Leica, CM3050S cryostat; chamber −16 °C to −18 °C, specimen holder −12 °C to −14 °C). One section per donor was mounted onto a Visium Spatial Gene Expression Slide (10x Genomics, PN-1000185). To minimize batch effects, sections from different donors were distributed across slides such that each slide combined at least one OCT + DEM, one OCT − DEM and one OCT_HC or two CEN + DEM and two CEN − DEM. Additionally, 7–10 adjacent sequential sections (10 µm) were collected onto SuperFrost Plus glass slides (Thermo Fisher Scientific). All slides were stored at −70 °C. For snRNA-seq, 15 sections (50 µm each) were collected per donor from the same frozen blocks and stored at −70 °C. The tissue distance between Visium and snRNA-seq sections ranged from 0.1 mm to 1 mm across donors.

### Nuclei isolation and single-nuclei RNA library preparation

Single nuclei were extracted from frozen tissue sections. In brief, OCT was removed by incubating sections in 1 ml of cold salt-Tris solution (146 mM NaCl, 10 mM Tris (pH 7.5), 1 mM CaCl_2_, 21 mM MgCl_2_, 1 mM 2-mercaptoethanol, 1× cOmplete protease inhibitor, 0.2 U µl^−1^ RNasin Plus) for 1 minute on ice, followed by centrifugation (500*g*, 3 minutes, 4 °C). The pellet was resuspended in 500 µl of ice-cold homogenization buffer (salt-Tris base supplemented with 25 mM KCl, 0.03% Tween 20, 0.01% BSA, 250 mM sucrose, 0.5 U µl^−1^ RNasin Plus) and homogenized in a KIMBLE Dounce grinder (Sigma-Aldrich) with 10 strokes of pestle A and 5–10 strokes of pestle B. The homogenate was filtered through a 70-µm strainer (Greiner Bio-One), incubated 5 minutes on ice and pelleted (500*g*, 5 minutes, 4 °C). The pellet was resuspended in 2.65 ml of homogenization buffer without Tween 20. All buffers and equipment were prechilled.

Nuclei were isolated via OptiPrep density gradient^63^. Homogenate was mixed with equal volume gradient medium (50% OptiPrep, 1 mM CaCl_2_, 5 mM MgCl_2_, 10 mM Tris (pH 7.5), 75 mM sucrose, 1 mM 2-mercaptoethanol, 0.5× cOmplete protease inhibitor, 0.5 U µl^−1^ RNasin Plus), layered onto 4 ml of 29% gradient medium and centrifuged (10,160*g*, 30 minutes, 4 °C; Beckman Coulter, SW41 Ti rotor). The nuclei pellet was resuspended in PBS with 1% BSA and 1 U µl^−1^ RNasin Plus and filtered through a 40-µm Flowmi strainer (Sigma-Aldrich). Final volume was 100 µl per sample.

Nuclei count and viability were assessed using AO/PI staining on a LUNA-FL counter (Westburg). We targeted 5,000–6,000 nuclei per sample, pooled per three samples and loaded onto a Chromium Next GEM Single-Cell 3′ Chip (10x Genomics). Library preparation followed the v.3.1 protocol (CG000204, Rev D). After quality control on an Agilent Bioanalyzer 2100, libraries were sequenced by BGI Tech Solutions using DNBSEQ paired-end sequencing.

### DAB staining

Adjacent cryosections (10 µm) were used to quantify local Aβ plaque and pTau load. Sections were post-fixed in 4% PFA/PBS for 10 minutes. For Aβ staining, antigen retrieval was performed by boiling in citrate buffer (0.01 M (pH 6.0), 700 W microwave, 10 minutes) followed by 70% formic acid (10 minutes). Sections were blocked with 5% fetal calf serum (30 minutes, room temperature). Primary antibodies (Thermo Fisher Scientific, anti-pTau AT8, 1:2,000; Signet, anti-amyloid 4G8, 1:10,000) were incubated overnight at 4 °C. Secondary antibodies (Dako, horse anti-mouse HRP, 1:400, or Dako, EnVision Detection System, K8023) were applied for 1 hour at room temperature, followed by ABC (Vector Laboratories) and DAB development (Dako). Sections were scanned at ×20 on an Axio Scan.Z1 (Zeiss). Two gray matter ROIs were selected per section. For quantification, a threshold of 3× background optical density was applied. The corrected integrated optical density (cIOD) was calculated as the optical density of positive area multiplied by its surface area, divided by total ROI area.

### Immunofluorescence, image acquisition and Visium Spatial Gene Expression

To detect Aβ, pTau and neuronal nuclei on the tissue sections of the OCT cohort used for Visium, we modified the immunofluorescence staining protocol provided by 10x Genomics (CG000312, revision version A). In brief, after methanol fixation at −20 °C for 30-minute and 5-minute incubation with blocking buffer (3 × SSC supplemented with 2% w/v BSA, 0.1% Triton X, 1 U ml^−1^ RNase inhibitor, TruStain FcX (1:35) and 20 mM ribonucleoside vanadyl complex) at room temperature, tissue sections were incubated consecutively with four different antibody solutions, prepared in blocking buffer. Tissues were first incubated with 10 µg ml^−1^ anti-Aβ 17-24 (4G8) antibody (BioLegend, 800701) and then with 10 µg ml^−1^ donkey anti-mouse IgG (H + L) Alexa Fluor 555 antibody (Thermo Fisher Scientific, A-31570). The third incubation step was a mixture of 30 µg ml^−1^ Alexa Fluor 647-conjugated anti-RBFOX3/NeuN antibody (1B7; Novus Biologicals, NBP1-92693AF647) and 3.33 µg ml^−1^ biotin-conjugated anti-pTau antibody (AT8) (Thermo Fisher Scientific, MN1020B), followed by a final incubation with a mixture of 3.4 µg ml^−1^ DAPI and 10 µg ml^−1^ Alexa Fluor 488-conjugated streptavidin. Each incubation lasted for 30 minutes at room temperature. After each incubation, five sequential wash steps with wash buffer (3 × SSC supplemented with 2% w/v BSA, 0.1% Triton X, 1 U ml^−1^ RNase inhibitor and ribonucleoside vanadyl complex) were applied as suggested by the company. To quench lipofuscin autofluorescence, the sections were treated for 30 seconds with 1× TrueBlack solution (Biotium, 23007), diluted in 70% ethanol, after which the slide was rinsed by dipping 15 times in 3 × SSC buffer. Finally, 200 µl of 80% glycerol with RiboLock RNase inhibitor 2 U µl^−1^ (Thermo Fisher Scientific, EO0382) was added. A coverslip was applied before imaging. To reduce the staining procedure time for the CEN cohort, we formed a pre-complex by incubating 3.33 µg ml^−1^ biotin-conjugated anti-pTau antibody (AT8) (Thermo Fisher Scientific, MN1020B) and 6.67 µg ml^−1^ Alexa Fluor 488-conjugated streptavidin for 30 minutes at room temperature prior to adding it to the tissue sections, in combination with 30 µg ml^−1^ Alexa Fluor 647-conjugated anti-RBFOX3/NeuN antibody (1B7) (Novus Biologicals, NBP1-92693AF647) and 3.4 µg ml^−1^ DAPI.

Next, 16-bit fluorescent images of DAPI, AT8, 4G8 and NeuN were acquired on an Axio Scan.Z1 slide scanner (Hamamatsu Orca Flash 4.0 V3 camera, Plan APO ×20/0.8 numerical aperture objective, ZEN Blue v.3.1). Channels were excited at 353 nm, 493 nm, 553 nm and 653 nm and collected with BP335-470-nm, BP500-550-nm, BP570-640-nm and BP665-715-nm emission filters, respectively. Images covered the entire Visium fiducial frame (8 × 8 mm) for downstream registration. Coverslips were removed by immersing slides at 45° into 3 × SSC buffer, followed by an additional wash. The entire procedure from methanol fixation through coverslip removal was kept under 5 hours. RiboLock RNase inhibitor (2 U µl^−1^; Thermo Fisher Scientific, EO0382) and/or ribonucleoside vanadyl complex (20 mM; New England Biolabs, S1402S) were included throughout to minimize RNA degradation.

After coverslip removal, tissues were immediately processed using the Visium Spatial Gene Expression Kit (10x Genomics, PN-1000186) and the Library Construction Kit (10x Genomics, PN-1000190), following the demonstrated protocol (CG000239, Rev D for OCT or Rev C for CEN). Tissues were permeabilized for 18 minutes (OCT) or 5 minutes (CEN) to release poly-adenylated mRNA onto Visium spots, followed by spatially barcoded reverse transcription. Second-strand synthesis was performed on-slide, after which cDNA was transferred to tubes for polymerase chain reaction (PCR) amplification and quality control on an Agilent Bioanalyzer 2100 High Sensitivity chip. Libraries were constructed through enzymatic fragmentation, size selection, end repair, A-tailing, adaptor ligation and sample index PCR (annealing at 67 °C for OCT/Rev D or 54 °C for CEN/Rev C, with recommended cycle numbers). Final libraries were quality controlled on a Bioanalyzer High Sensitivity chip and sequenced via paired-end DNBSEQ technology (BGI Tech Solutions).

### Xenium in situ gene expression, immunofluorescence and image acquisition

Xenium in situ gene expression was performed at LISCO (Leuven, Belgium) on five OCT + DEM and six OCT − DEM samples. Tissue sections on Xenium slides were fixed and permeabilized using Xenium Sample Prep reagents (10x Genomics, PN-1000460) following the demonstrated protocol (CG000581). In situ gene expression was performed using a standalone Xenium Custom Gene Expression Panel following protocols for probe hybridization, ligation and amplification (CG000582) and Xenium Analyzer operation (CG000584). In brief, fixed and permeabilized sections were incubated overnight with the custom probe panel, followed by post-hybridization washes, ligation to generate circular DNA probes and rolling circle amplification. An autofluorescence quencher and DAPI nuclear stain were applied before loading Xenium cassettes into the analyzer for imaging and decoding (consumables PN-1000487).

After the Xenium run, sections were stored in 750 µl of 1× PBS at 4 °C (maximum 1 week) before downstream immunofluorescence for Aβ, pTau and NeuN. The autofluorescence quencher was removed with 10 mM sodium hydrosulfite (10 minutes, room temperature) and 3× water rinses. Sections were blocked (1× PBS, 5% normal donkey serum, 0.5% Triton X; 90 minutes, room temperature) and sequentially incubated with (1) 5 µg ml^−1^ anti-Aβ 4G8 (BioLegend, overnight, 4 °C); (2) 5 µg ml^−1^ donkey anti-mouse Alexa Fluor 555 (90 minutes, room temperature); (3) 15 µg ml^−1^ Alexa Fluor 647-conjugated anti-NeuN mixed with 2 µg ml^−1^ biotin-conjugated AT8 (2 hours, room temperature); and (4) 1 µg ml^−1^ DAPI mixed with 10 µg ml^−1^ Alexa Fluor 488 streptavidin (1 hour, room temperature). Sections were washed three times for 10 minutes with wash buffer (1× PBS, 0.5% Triton X) between incubations. Lipofuscin autofluorescence was quenched with 1× TrueBlack in 70% ethanol (30 seconds), rinsed in 3 × SSC and mounted with 20% SlowFade Diamond (Thermo Fisher Scientific, S36967) in 80% glycerol.

Then, 12-bit fluorescent images were acquired on a Nikon Ni-E Eclipse (Digital Sight 10 camera, Plan APO ×20/0.8 numerical aperture, NIS-Elements v.3.1). DAPI, AT8, 4G8 and NeuN were excited at 400 nm, 470 nm, 555 nm and 635 nm (CoolLED, pE800) and collected with BP335-470-nm, BP500-550-nm, BP570-640-nm and BP665-715-nm emission filters, respectively.

### Image analysis

For Visium, 16-bit fluorescence images were aligned with the sequenced Visium array using the fiducial frame. The 555-channel contrast was adjusted to visualize fiducial markers and tissue boundaries; images were rotated to correct orientation; and the channel was exported as an 8-bit grayscale single-page TIFF at full resolution. These TIFFs were uploaded to Loupe Browser 5.0 (10x Genomics) for manual fiducial alignment, after which Visium spot coordinates were automatically calculated based on the aligned frame and slide ID. Tissue boundaries were indicated, and an aligned JSON file containing tissue-overlapping spot IDs and pixel coordinates was generated and passed to Space Ranger to compute per-spot transcriptomic profiles.

For Xenium, fluorescent pathology images (Aβ, pTau, NeuN and DAPI) were aligned to the Xenium DAPI image in Fiji^64^ using the ‘register virtual stack slices’ plugin (affine model). The higher-resolution Xenium DAPI was downscaled to match the pixel size of the pathology images before serving as alignment reference.

Pathology annotations were performed in QuPath (v.0.2.3)^65^ on aligned fluorescent images. A tissue mask was manually annotated to remove artifacts and subdivided into white matter and cortical layers based on NeuN and DAPI morphology. Vessels and pia were annotated separately to account for non-specific antibody staining. Aβ plaques and pTau pathology were annotated using pixel intensity thresholds and size filters. Plaques were further classified as core plaques, neuritic plaques or diffuse plaques based on neuropathological and morphological criteria: neuritic plaques were characterized by dystrophic neurites surrounding plaques, core plaques by circled-dot morphology (Fig. 1b) and diffuse plaques by lower Aβ intensity and heterogeneity. Diffuse plaques were distinguished from neuritic plaques/core plaques using the 15th percentile of ranked pixel intensity standard deviations. Annotations overlapping vessels and pia were removed.

For Visium, spot boundaries were computed, and fluorescent channel intensity measures (mean, median and standard deviation for Aβ, pTau, NeuN and DAPI) were recorded per spot. The resulting metadata matrix detailing ROI overlap per spot was used for downstream transcriptomic analysis.

### Visium spot selection and analysis

Unbiased clustering of Visium spots by transcriptomic profiles revealed patterns consistent with cortical layer identity, individual variability and pathological heterogeneity (Fig. 1f). To relate local transcriptomic variation to protein aggregation, we quantified the proportion of each spot overlapping with Aβ and pTau pathology. As pTau accumulation was predominantly observed in cortical layers 3–5, particularly in OCT + DEM cases (Fig. 1c), subsequent analyses focused on these layers.

### Centenarian cohort analysis

The centenarian cohort was not subdivided by dementia status due to insufficient separation in clinical data. CEN brains showed pTau and plaque morphologies with no significant differences in Aβ plaque density compared to OCT ± DEM cases.

### Human FFPE brain samples

FFPE brain samples from three human autopsy cases were analyzed (clinical and pathological details in Supplementary Table 1). Individuals were selected based on absence of encephalitis/meningitis and presence of symptomatic AD, defined by clinical dementia and intermediate-to-high AD neuropathological changes. Autopsies were performed at UZ Leuven (Belgium) in compliance with national regulations, with written informed consent and ethical approval from the UZ Leuven Ethics Committee. The right hemisphere was dissected for gross neuropathological examination and stored at −80 °C. The left hemisphere was fixed in 4% formaldehyde for 2–4 weeks and then dissected and macroscopically evaluated. Frontal cortex samples were collected for further analyses. CDR scores^66^ were retrospectively assigned from medical records.

Left hemisphere tissue blocks were paraffin embedded and sectioned at 5 µm onto Flex IHC adhesive slides (Dako), dried at 55 °C and stained with hematoxylin and eosin for diagnostic evaluation. AD pathology was assessed via immunohistochemistry using established criteria: Aβ plaque deposition (Aβ phase)^67^, tau neurofibrillary tangle distribution (Braak neurofibrillary tangle stage)^68^ and neuritic plaque density (CERAD score)^69^. The NIA−AA score^60^ was determined from these three indices.

### Immunofluorescence for HLA and SPP1

Immunofluorescence was performed on frontal cortex FFPE AD and frozen centenarian brain sections. FFPE sections were deparaffinized and subjected to heat-induced epitope retrieval (pH 6.1). Frozen sections were heated briefly (1 minute, 37 °C) and fixed in 4% PFA (20 minutes). Amyloid plaques were stained with X34 (20 minutes, room temperature). Frozen sections were blocked and permeabilized (5% BSA, 0.2% Triton X-100 in PBS). Primary antibodies were applied overnight at room temperature, followed by fluorophore-conjugated secondary antibodies or streptavidin. IBA1 served as microglial marker for FFPE tissue and CD45 for frozen tissue. Autofluorescence was quenched with TrueBlack (30 seconds; Biotium). Slides were mounted in Glycergel (Agilent Technologies). Antibody details are listed in Supplementary Table 10. Images were acquired on a Nikon A1R confocal system coupled to a Nikon Eclipse Ti inverted microscope using NIS-Elements. Image processing and figure assembly were performed in ImageJ (National Institutes of Health) and Inkscape (https://inkscape.org/).

### snRNA-seq processing

Raw FASTQ files were processed using Cell Ranger v.6.0.1 (10x Genomics) with default settings, aligning to the human GRCh38 genome (prebuilt reference 2020-A) for filtering, barcode counting and unique molecular identifier (UMI) quantification.

Because snRNA libraries contained pooled individuals, feature−barcode matrices were demultiplexed using Souporcell (v.2.0)^70^ based on genetic profiles from the Illumina Global Screening Array (GSA). Cell Ranger BAM files were used to calculate allele frequency tables per library. Souporcell identified variants, computed allele frequencies and clustered cells by individual. Clusters were matched to reference genotype profiles via Pearson correlation, with assignments made at greater than 90% similarity. Sample IDs were added as metadata to the feature−barcode matrix.

Count matrices were imported into Scanpy (v.1.8.2)^71^. Cells with fewer than 200 genes, genes in fewer than five cells and cells with more than 20% mitochondrial reads were filtered. Counts were normalized (total count normalization, scale factor 10,000) and log transformed.

Doublets were removed using Scrublet^72^ (threshold 0.39). Remaining cells were clustered using scVI (scvi-tools v.1.1.6)^73^. Highly variable genes (HVGs) were selected accounting for sequencing batch (Scanpy highly_variable_genes: min_mean = 0.0125, max_mean = 3, min_disp = 0.5, batch_key = batch). This yielded 112,698 single nuclei with a mean of 3,399 genes per nucleus (IQR: 1,563–4,663) for the OCT cohort. An overview is provided in Supplementary Data 1.

### Visium spatial transcriptomics processing

Raw Visium FASTQ files were processed using Space Ranger 1.2 (10x Genomics) with the GRCh38 reference (2020-A). The filtered count matrix was combined with spot positions and scaling factors for integration with Visium image analysis data. Inclusion criteria were as follows: ≥200 counts per gene, ≥10 genes per spot and ≥500 reads per spot. Staining artifacts, low-quality tissue regions, vessels and samples with poor RNA quality were removed. The curated dataset comprised 60,129 spots from 24 individuals (mean 1,831 genes, IQR: 1,168–2,352). The same approach yielded 61,739 spots from 20 centenarian individuals.

After removing spots with imaging artifacts or outside the tissue mask, the top 2,000 HVGs were selected. Samples were integrated using scVI (v.1.1.6)^73^, batch correcting for RIN (continuous covariate) and gyrus frontalis superior (GFS) location and Visium slide (categorical covariates). The scVI latent representation was used to construct a *k*-nearest neighbor graph for UMAP^74^ visualization (min_dist = 0.2) and Leiden clustering (resolution = 0.2).

### Cell deconvolution

To map cell types and states from snRNA-seq onto Visium data, we used cell2location (v.0.1)^48^. The snRNA-seq reference was trained using the RegressionModel (batch_size = 2,500, train_size = 1, lr = 0.002, max_epochs = 200), with sequencing lane ID as batch covariate, GFS as categorical covariate and RIN_NBB as continuous covariate. Reference cell state signatures were estimated by simulating 1,000 posterior samples with the same batch size. For spatial deconvolution, cell2location was run with N_cells_per_location = 8, detection_alpha = 200, max_epochs = 30,000, batch_size = None and train_size = 1, using Visium slide ID as batch covariate alongside GFS and RIN_NBB. Posterior samples were drawn using the same configuration as the reference model. In downstream analyses, the 5% quantile of the posterior distribution (q05_cell_abundance) was used as a conservative estimate of cell type abundance per Visium spot.

### TD clustering and visualization

Clustering was performed on the cell2location cell type abundance matrix. Abundances were normalized to *z*-scores based on total cell abundance per spot. Mid-layer (layers 3–5) Visium spots from 24 OCT individuals were used. To mitigate layer-specific variation while preserving pathology-associated biology, Harmony integration^49^ was applied on layer (Scanpy harmony_integrate, 20 principal components, max_iter_harmony = 20). The same procedure was applied to 20 CEN individuals. Leiden clustering was performed on cell type abundance profiles using a *k*-nearest neighbor graph (20 neighbors) in Harmony-integrated principal component analysis space (random seed 42, resolution 0.4), yielding six TDs for OCT (OCT-TD0 to OCT-TD5) and seven TDs for CEN (CEN-TD0 to CEN-TD6). Results were visualized using UMAP.

To address overplotting, hexbin plots (Matplotlib^75^) were used, with hexbins containing fewer than five spots grayed out. Three coloring schemes were applied: (1) percentage score (fire colormap) for categorical variables, showing the proportion of spots associated with a given category; (2) average score for the aggregated mean of continuous variables per hexbin; and (3) mean difference score (blue/red colormap) reflecting the difference in *z*-normalized means of continuous variables or gene set module scores between OCT + DEM and OCT − DEM spots per hexbin.

### Differential gene expression analysis

Differential gene expression analysis was performed on Visium data at different pathology and phenotype levels using edgeR^76^. Pathology load was calculated as the log1p-transformed percentage of each spot covered by the specific pathology ROI. Spots from layers 3–5 (100% overlap) were extracted. A quasi-likelihood negative binomial generalized log-linear model was applied for genewise statistical tests with robust prior quasi-likelihood dispersion estimation.

### Differential cell abundance analysis

Differential abundance analysis was performed using edgeR on the same layer 3–5 Visium spots used for differential gene expression analysis, leveraging negative binomial generalized linear model methods to model the overdispersed cell abundance matrix. Cell2location-predicted abundances were normalized by total cells per sample. The percentage overlap of pathology per spot, derived from image analysis, was included as continuous covariates in the design formula and tested using glmQLFTest for each phenotype separately. The analysis yields log_2_ fold changes and FDR-corrected *P* values (Benjamini−Hochberg), enabling assessment of cell abundance changes in response to plaque and pTau pathology across phenotypes.

### TD prediction in the CEN cohort

We trained an MLP from scikit-learn (http://jmlr.org/papers/v12/pedregosa11a.html) trained on data from the OCT cohort to predict the assigned OCT TD based on the underlying, normalized, cell type abundance matrix. We subsequently applied this predictor to the CEN cell2location abundance matrix to predict in what OCT TD each CEN Visium spot would fall.

### Gene co-expression analysis of Visium Spatial Gene Expression

Signed co-expression networks were constructed using WGCNA (R, v.1.72.5)^77^ on Visium spatial transcriptomic data from gray matter regions of the OCT cohort (17,271 genes, 41,943 spots) and the CEN cohort (18,090 genes, 39,358 spots) independently. Raw count matrices were normalized and variance stabilized using SCTransform (Seurat 4.0). A soft power of 14 was selected via pickSoftThreshold. Modules were identified using cutreeDynamic (deepSplit = 2) with a minimum of 30 genes per module, yielding 16 modules for the OCT cohort and 20 for the CEN cohort.

### Xenium data processing

Xenium in situ sequencing was performed on 11 samples (six OCT − DEM and five OCT + DEM) using a panel of 329 genes, including cell type/subtype markers selected via scMAGS (v.1.5)^78^ and genes of interest identified from Visium analysis. scMAGS selects markers highly expressed in a specific cell type with low expression in others, making them well suited for spatial transcriptomics. Raw Xenium data were preprocessed using Xenium Ranger (v.1.7). Cell segmentation was performed on DAPI nuclear staining across *z*-stack planes, generating non-overlapping two-dimensional nuclear masks projected onto the *x*−*y* plane. Masks were expanded by 5 µm or until intersecting another cell boundary, providing approximate cell segmentation. Transcripts within boundaries were assigned to cells, producing a per-cell count matrix.

For cell type identification, quality filters were applied: nuclear area 9.5–130 µm^2^, transcript counts 13–900, cell area 11.92–444 µm^2^ and ≥0.8 transcripts per µm^2^. Doublets were removed using Scrublet (v.0.2.3, threshold 0.82), retaining 424,056 cells. Clustering was performed using Scanpy (v.1.9.1) and scVI (v.0.19.0). HVGs were identified based on mean expression (min_mean = 0.125, max_mean = 30) and dispersion (min_disp = 0.5) across cassette batches, retaining genes variable in more than two batches. An scVI model was trained with cassette name as batch covariate, GFS as categorical covariate and RIN_NBB as continuous covariate. The scVI latent space was used for *k*-nearest neighbor graph construction, UMAP visualization (min_dist = 0.5) and Leiden clustering (resolutions 0.4, 0.6 and 1.0). Identified populations included oligodendrocyte precursor cells (11,794), vascular cells (45,816), astrocytes (53,569), excitatory neurons (64,010), inhibitory neurons (23,401), microglia (25,926) and oligodendrocytes (199,540).

### Xenium differential expression analysis

For differential expression analysis relative to pathology in the Xenium dataset, we quantified each cell’s spatial proximity to pathological features. Using pathology masks defined during image analysis, the distance from each cell to the nearest edge of plaque or pTau pathology was calculated as the average Euclidean distance and then log1p-transformed and included as continuous covariates in gene-level differential expression analysis using edgeR. The A axis differential expression was calculated by selecting cells >25 µm from pTau pathology and testing against Aβ distance. For the T axis, cells <25 µm from Aβ were selected, and differential expression was performed against pTau distance.

### scANVI label transfer

To further annotate our single-cell data, we performed label transfer from the SEA-AD study^34^ using scANVI (scvi-tools v.1.1.6)^53^. For each cell type separately, both datasets were preprocessed and embedded with scVI using raw counts and donor ID as batch key. The scVI model was trained for up to 200 epochs and then used to initialize scANVI with the SEA-AD ‘Supertype’ as label key. scANVI was trained for an additional 200 epochs to predict subtype labels for the octogenarian cells. To assess overlap between scANVI-predicted subtypes and Leiden clusters, we performed a *χ*^2^ test of independence on a cross-tabulation of cluster and label assignments. Standardized residuals were calculated by normalizing observed versus expected values and dividing by the square root of expected values.

### MAGMA analysis of GWAS results

We employed MAGMA^51^ v.1.10 to aggregate the GWAS summary data from Kunkle et al.^24^ to individual genes, selecting genes with an aggregate *P* value better than 5 × 10^−8^.

### Statistical analysis

All statistical analyses were performed in Python (SciPy v.1.13.1 and statsmodels v.0.14.5).

#### Pairwise group comparisons

To compare the average pathology overlapping with tissue domains per sample between phenotypic groups (Fig. 1d), pairwise, two-sided Mann−Whitney *U*-tests were used.

#### Enrichment within TDs

Enrichment of a group or statistic within each TD cluster (Figs. 2b,c, 3e,f,i,j and 5e,f and Supplementary Fig. 2) was assessed comparing the distribution of values per hexbin inside a given TD against the pooled distribution from all other TDs using a one-sided Mann−Whitney *U*-test (alternative = greater, testing for enrichment in a TD).

#### Delta hexbin plots

Figures 1d, 3g,k and 5g use the same comparison but apply a two-sided Mann−Whitney *U*-test.

#### Pathology differences across cortical layers

To examine pathology distribution across cortical layers (Fig. 1c), Aβ and pTau levels were expressed as log-transformed mean percentages per layer and donor. Under the null hypothesis of no group effect, phenotype labels were permuted within each layer while preserving donor-level distributions (5,000 permutations). For each permutation, a between-group test statistic was recomputed and compared with the observed statistic to obtain empirical *P* values, Bonferroni corrected across layers (*α* = 0.05).

#### Cell type enrichment within TD clusters

Cellular enrichment within TDs (Fig. 2d,e) was assessed using cell2location abundance estimates. Expected values under independence were calculated from group means (per TD and per cell type). Enrichment was quantified as log_2_(observed / expected). One-sided *P* values for overenrichment were derived from standardized residuals and Bonferroni corrected for multiple testing.

#### Multiple testing correction and significance thresholds

For all multiple pairwise or multicluster comparisons, Bonferroni correction was applied to control the familywise error rate at 5%. Unless otherwise noted, tests were two-sided with corrected *P* < 0.05 considered significant.

#### Glial Gene Ontology enrichment

Gene Ontology Biological Process enrichment was performed on glial subtypes using GSEA. Ranked gene lists were constructed from pseudobulk differential expression between subtypes and analyzed with gseGO in clusterProfiler (R, v.4.6.0). Gene sets of 10–300 genes were included, and Gene Ontology terms with Bonferroni-adjusted *P* < 0.05 were considered significant.

To compare enrichment across subtypes, NESs and adjusted *P* values were merged into a matrix. Dot plots were generated with ggplot2, encoding NES by color and significance by point size. A subset of biologically informative, subtype-distinguishing Gene Ontology terms was selected for final visualizations.

### Reporting summary

Further information on research design is available in the Nature Portfolio Reporting Summary linked to this article.

## Online content

Any methods, additional references, Nature Portfolio reporting summaries, source data, extended data, supplementary information, acknowledgements, peer review information; details of author contributions and competing interests; and statements of data and code availability are available at 10.1038/s41591-026-04393-8.

## Supplementary information

Supplementary Information Supplementary Data 1 and 2 and Supplementary Figs. 1 and 2

Reporting Summary

Peer Review File

Supplementary Table 1 Cohort and donor information

Supplementary Table 2 Histology quantification by region and by phenotype

Supplementary Table 3 PIGs and WGCNA gene sets

Supplementary Table 4 Gene Ontology enrichment of gene co-expression networks

Supplementary Table 5 Single-nuclei label transfer

Supplementary Table 6 Microglial subtype differential expression analysis

Supplementary Table 7 Early PIG and late PIG and glial cell state Gene Ontology enrichment

Supplementary Table 8 Layer-specific differential expression analysis

Supplementary Table 9 Xenium gene panel

Supplementary Table 10 Antibody information

## Figures and Tables

**Extended Data Fig. 4:**
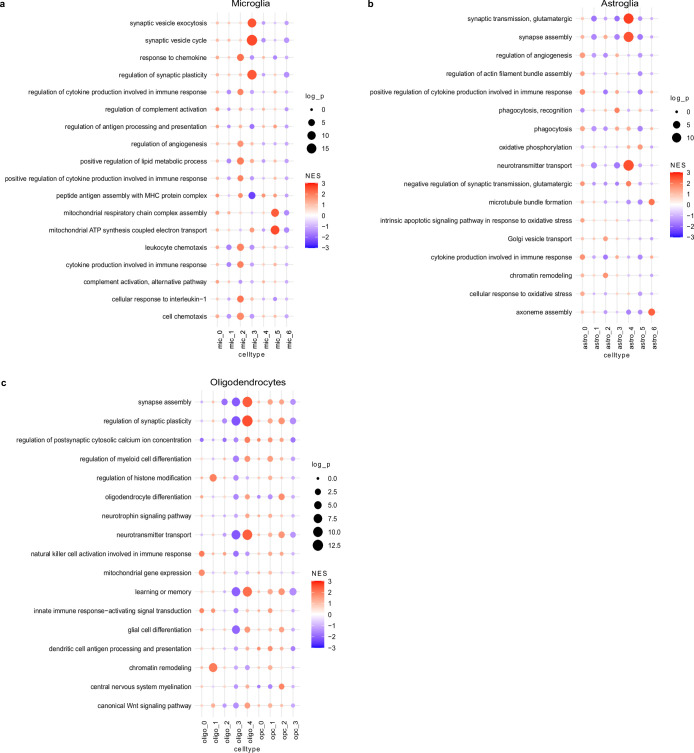
Glial subpopulation GO enrichment analysis. (**a**) GO enrichment analysis of microglia states using GSEA against differential expression of each microglia state versus all others (see Methods for details). The same plots are show for astrocytes (**b**) and OPC’s and oligodendrocytes in (**c**).

## Data Availability

For reasons of ethics and privacy, raw sequencing reads of all single-cell experiments (scRNA-seq, Visium and Xenium) have been deposited in the European Genome-phenome Archive (EGA) under study number EGAS50000001692 and with data accession numbers EGAD50000002431 (Visium data) and EGAD50000002432 (snRNA-seq) (to access the data itself under restricted access). Requests for accessing raw sequencing reads must be submitted to the EGA and will be reviewed by the VIB Data Access Committee to determine whether the proposed reuse of the data is not incompatible with the goal for which the data were originally generated. Decisions on access will be taken within a reasonable timeframe after having received the necessary information. Any data shared will be released via a data transfer agreement that will include the necessary conditions to guarantee protection of personal data (according to European General Data Protection Regulation law).
