# Supplementary material for: Human microglial transitions at the Aβ–tau inflection point associate with divergent pathways to dementia and resilience
Source: Nat Med. 2026 Jun 4;32(6):2047–59. doi: 10.1038/s41591-026-04393-8 (PMC13278961; doi:10.1038/s41591-026-04393-8)
Supplement: Supplementary file 1 — Supplementary Data 1 and 2 and Supplementary Figs. 1 and 2 [file 41591_2026_4393_MOESM1_ESM.pdf]

nature medicine

Supplementary information

https://doi.org/10.1038/s41591-026-04393-8

Human microglial transitions at the Aβ–tau inflection point associate with divergent pathways to dementia and resilience

In the format provided by the authors and unedited

Supplemental Figure S1a-h: Neuronal cellular subpopulations defined by single nuclei transcriptomics

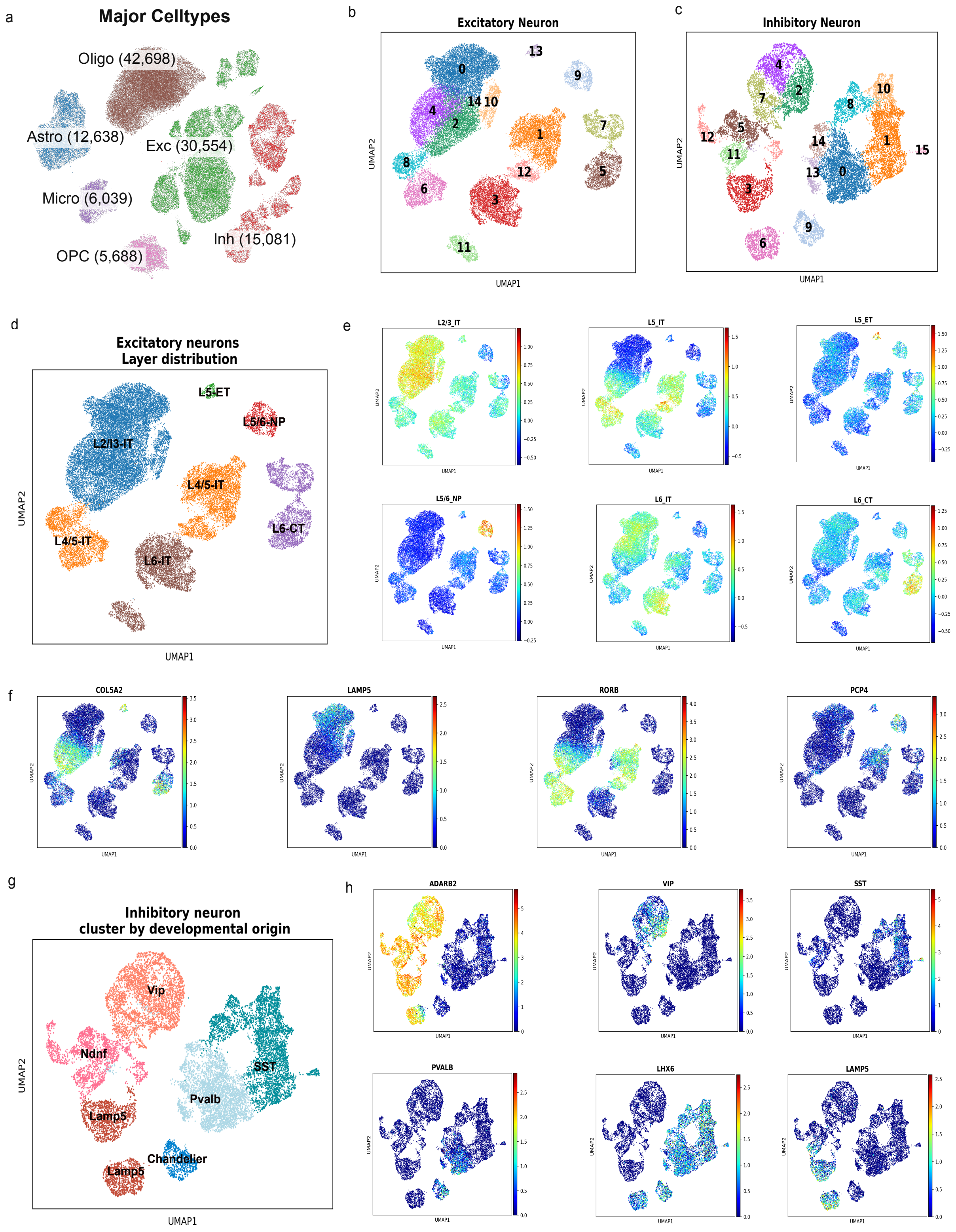

Supplemental Figure S1i-p: Glia cell subpopulations defined by single nuclei transcriptomics.

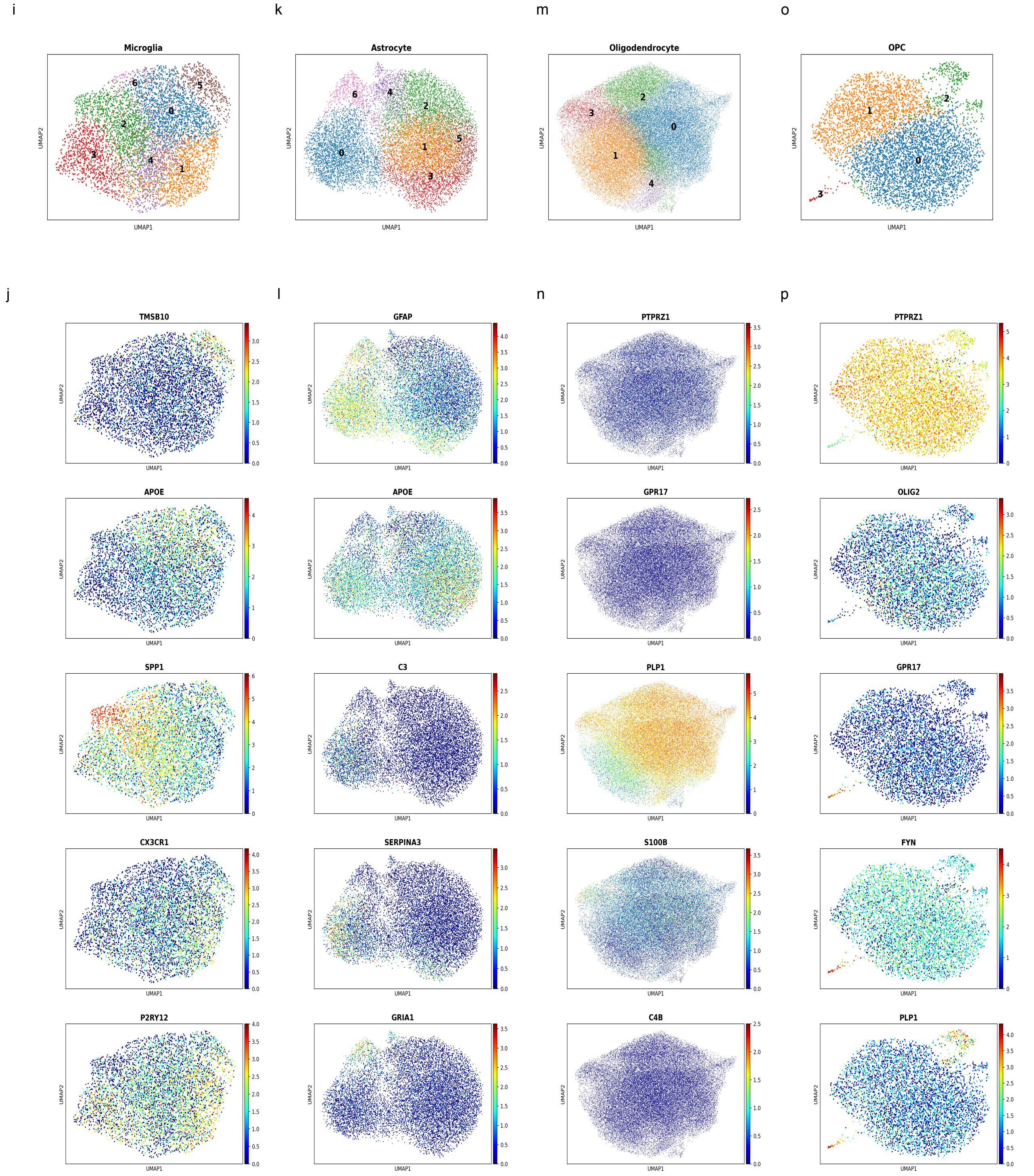

Supplemental Figure S1: Single-nucleus transcriptomics UMAPs

All marker genes were chosen based on literature, summarized in Suppl. Data A.
(a-c) UMAP plots of single-nuclei transcriptomes from the octogenarian cohort. (a) All 112,698 nuclei. (b) subset of 30,554 excitatory neurons. (c) subset of 15,081 inhibitory neurons.
(d, e) Layer-specific characterization of excitatory neurons. (d) Summary of excitatory clusters organized by cortical layer specificity (Table S8). Identified types include: four L2/3 intratelencephalic (IT) clusters, four L4/L5 IT clusters, two L6 IT clusters, one L5 extratelencephalic (ET) cluster, one L5/6 near-projecting (NP) cluster, and two L6 corticothalamic (CT) clusters. (e) UMAP of excitatory neurons (from b) colored by module scores for layer-specific gene sets (Table S8).
(f) UMAP of excitatory neurons, colored by expression levels of layer specific markers, LAMP5, RORB, COL5A2 and PCP4[1].
(g, h) Characterization of inhibitory neurons. (g) UMAP of inhibitory neurons (from c), categorized according to the subclasses defined by Tasic *et al.*[2], including four Sst-, three Pvalb-, three Lamp5-, and three Vip-positive populations. (h) UMAP of inhibitory neuron colored by expression of subtype markers[2].
(i & j) Microglia. (i) UMAP of 6,039 microglia showing unsupervised clustering. (j) UMAP of microglia colored by expression of canonical microglial marker genes[3].
(k & l) Astrocytes. (k) UMAP of 12,638 astrocytes showing transcriptomic diversity. (l) UMAP colored by expression of known astrocytic marker genes[4].
(m & n) Oligodendrocytes
(m) UMAP of 42,698 oligodendrocytes, showing clustering of mature oligodendrocyte states.
(n) UMAP colored by expression of oligodendrocyte marker genes[5].
(o & p) Oligodendrocyte precursor cell (OPC).
(o) UMAP of 5,688 OPC. (p) UMAP colored by expression of known OPC marker genes[5].

Supplemental Figure S2: Hexbin visualization of cell type abundance overlaid on tissue domain (TD) UMAP.

(a) Major cell-type abundances projected onto the TD UMAP.

(b–g) cellular subtypes projected onto the TD UMAP: astrocytes (b), excitatory neurons (c), inhibitory neurons (d), microglia (e), oligodendrocytes (f), and OPCs (g). Hexbin plots display the average Cell2location-inferred abundance within each UMAP bin. Heatmaps below each panel summarize mean abundance per TD cluster.

Enrichment was assessed by comparing the distribution of abundance values within each TD to the distribution across those all other TDs using one-sided Mann–Whitney U tests, followed by Bonferroni correction. Significance: * p-adj < 0.05, **p-adj < 0.01, *** p-adj < 0.001.

Supplemental Figure S2a: - Tissue Domain Hexbin UMAP representation of top level cell types

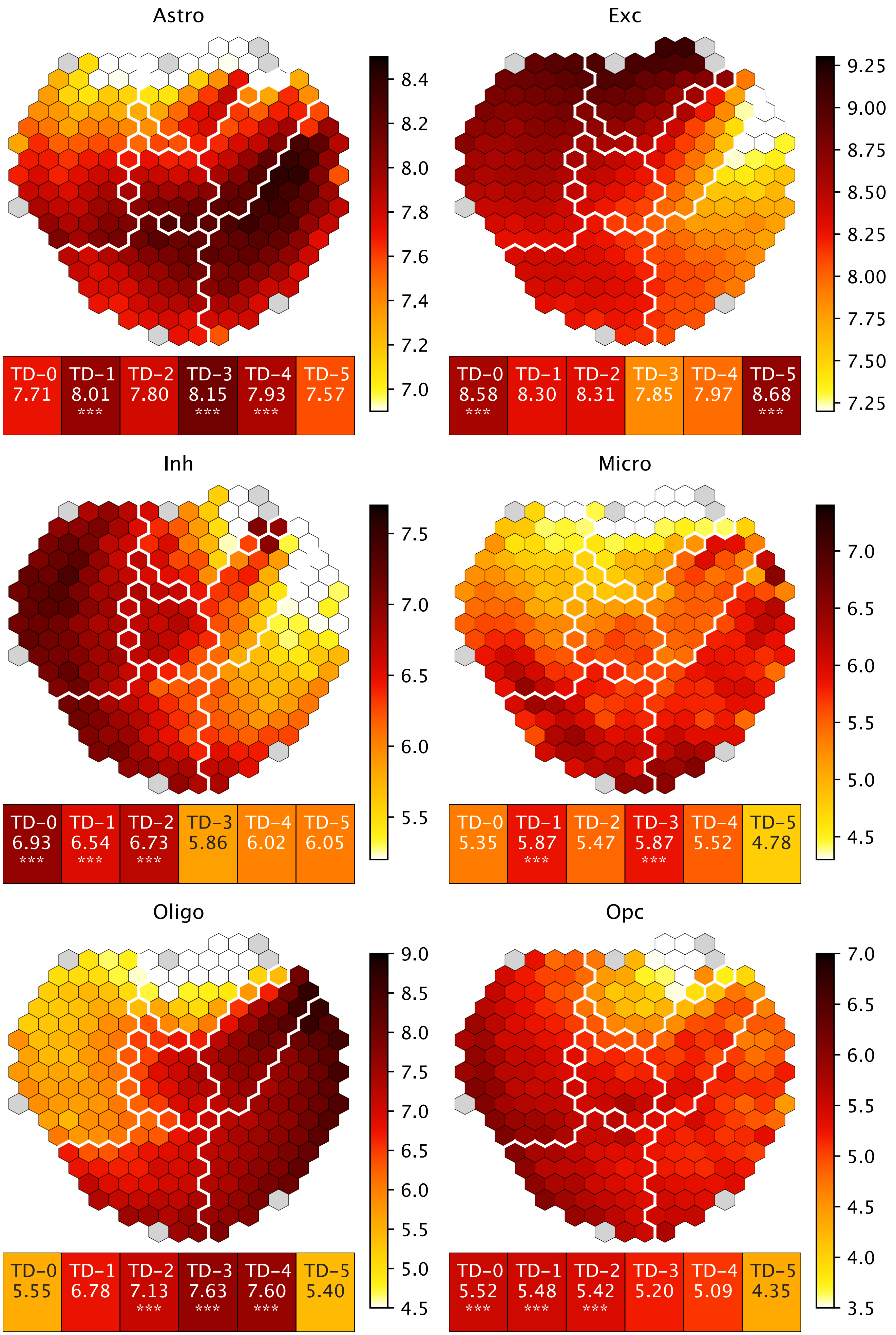

Supplemental Figure S2b: - Tissue Domain Hexbin UMAP representation of astrocytes

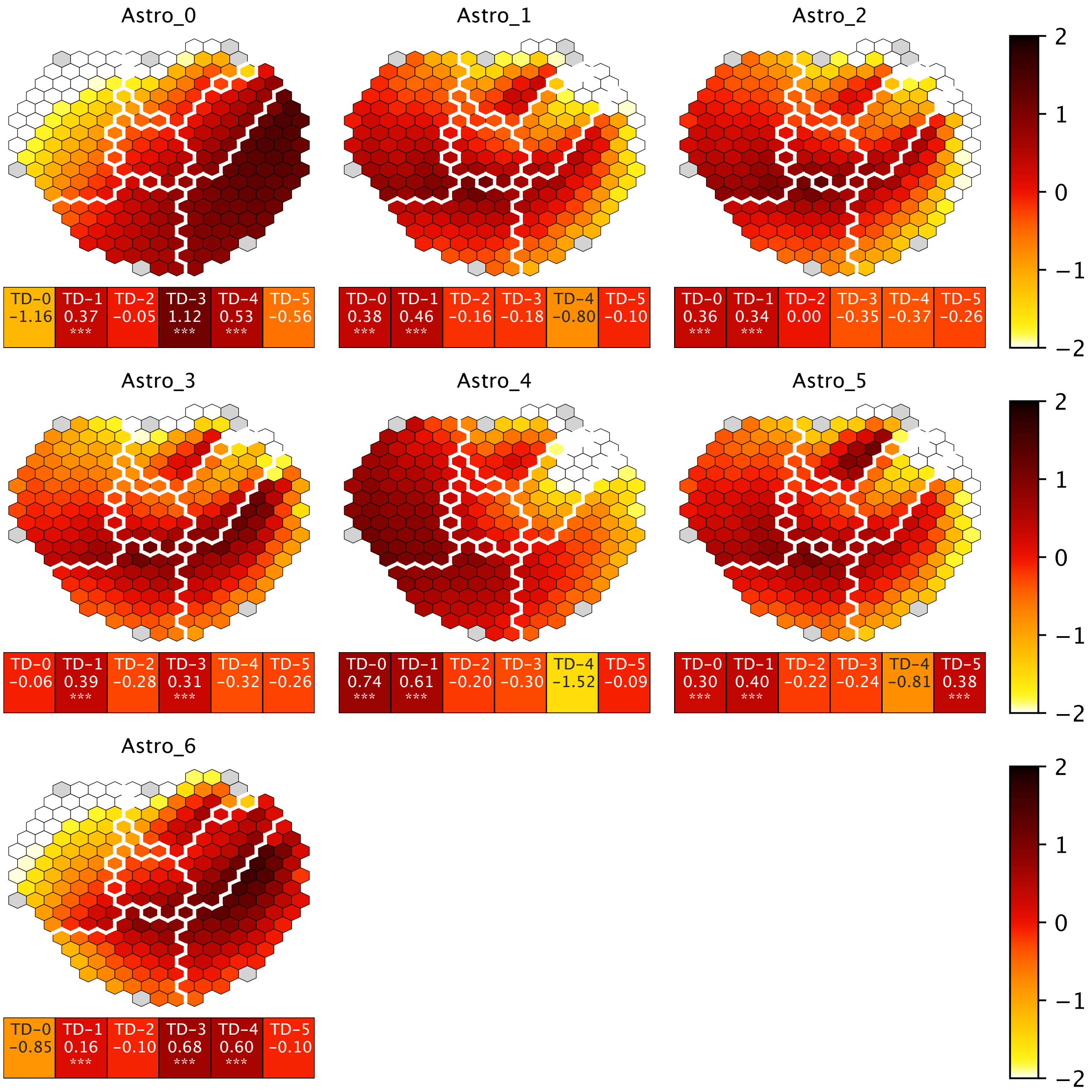

Supplemental Figure S2c: - Tissue Domain Hexbin UMAP representation of excitatory neurons

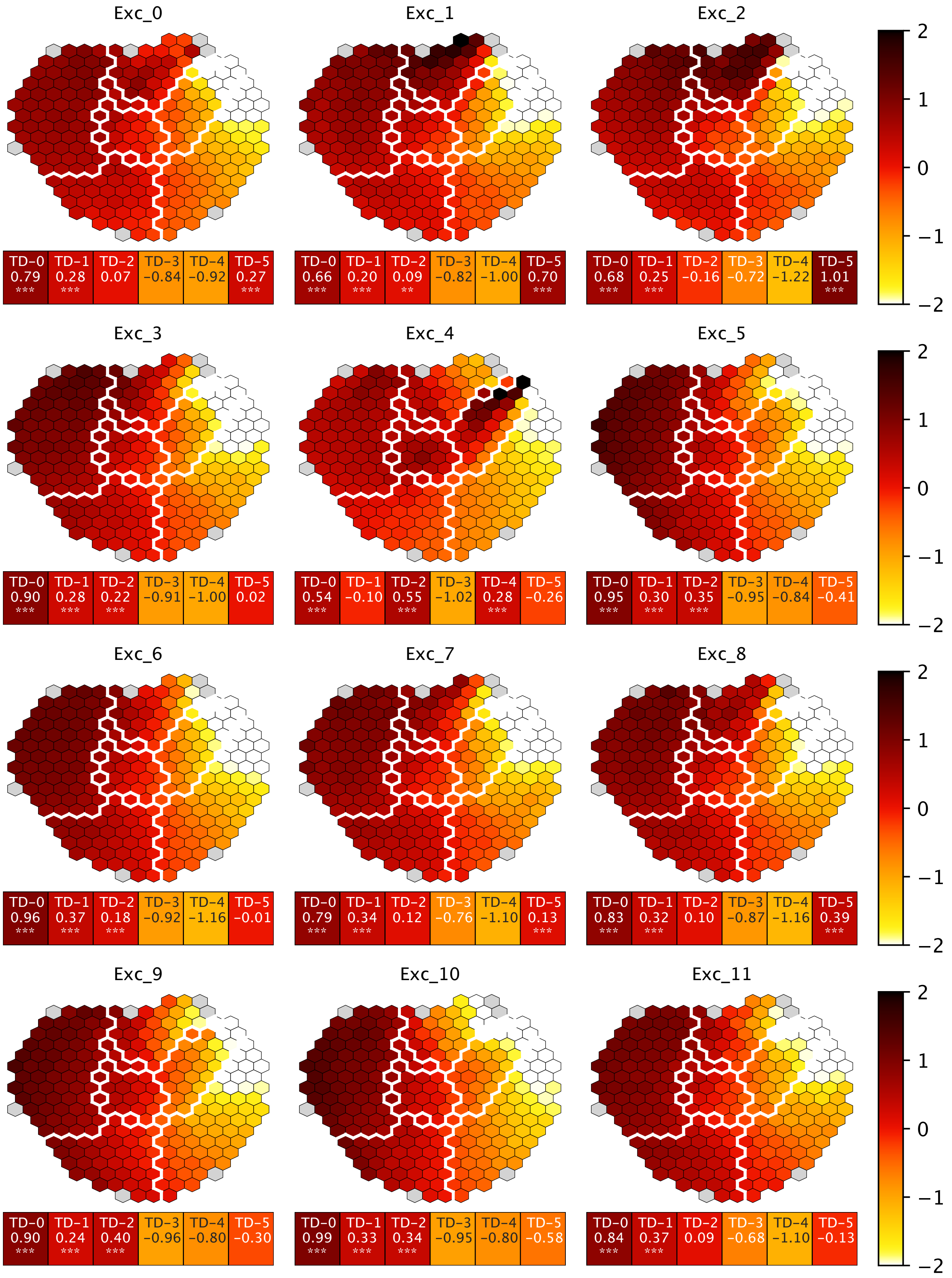

Supplemental Figure S2c bis: - Tissue Domain Hexbin UMAP representation of excitatory neurons

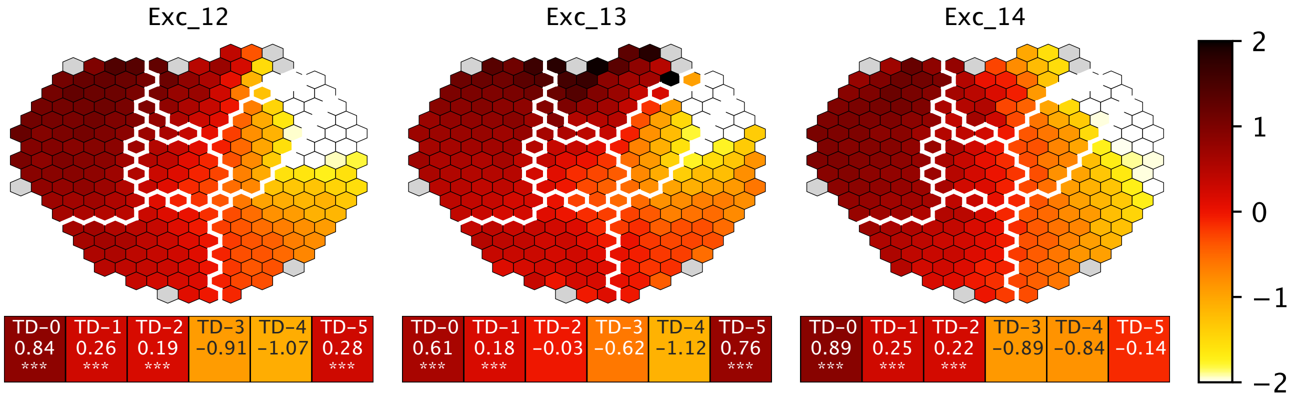

Supplemental Figure S2d: - Tissue Domain Hexbin UMAP representation of inhibitory neurons

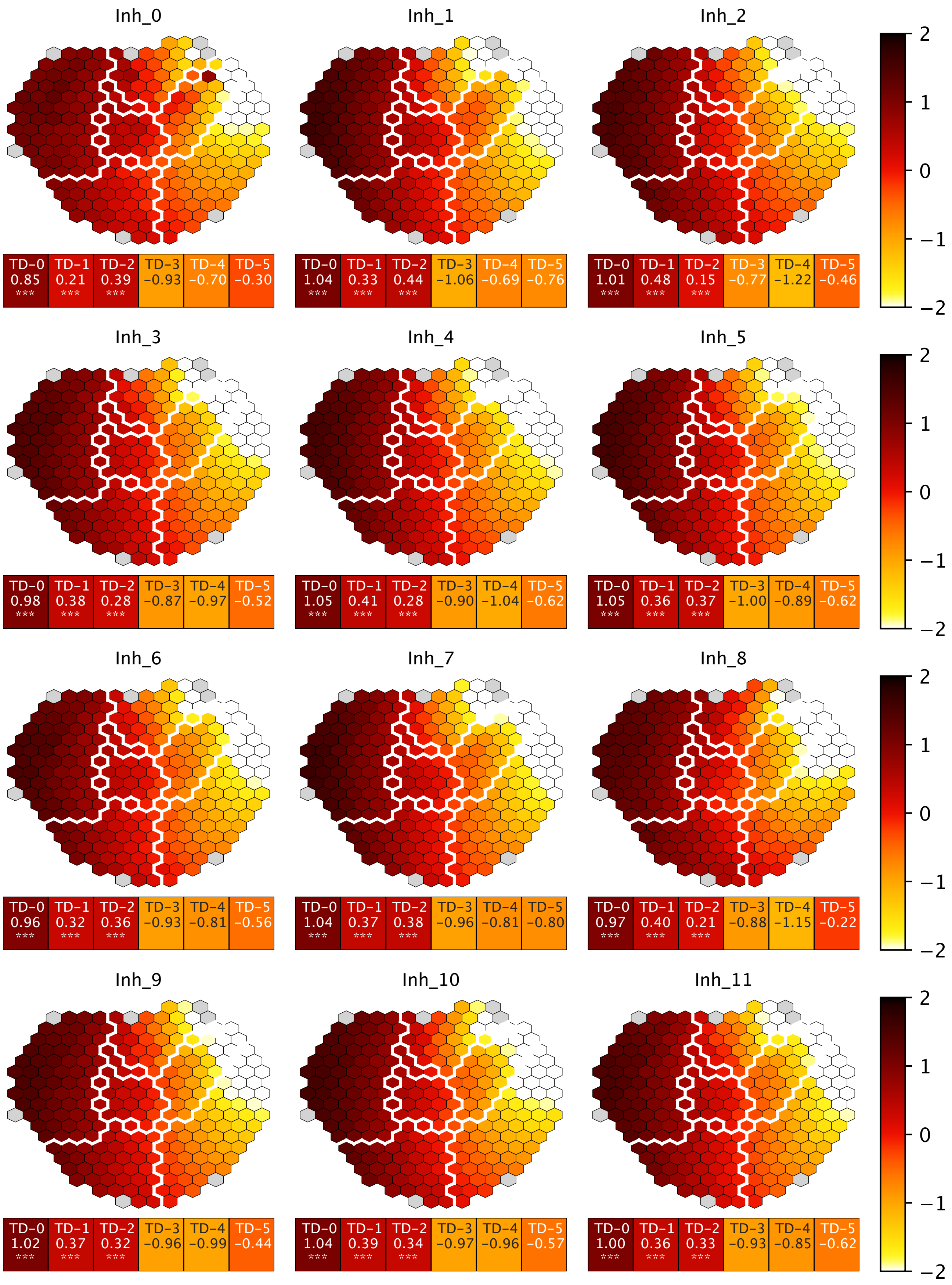

Supplemental Figure S2d bis: - Tissue Domain Hexbin UMAP representation of inhibitory neurons

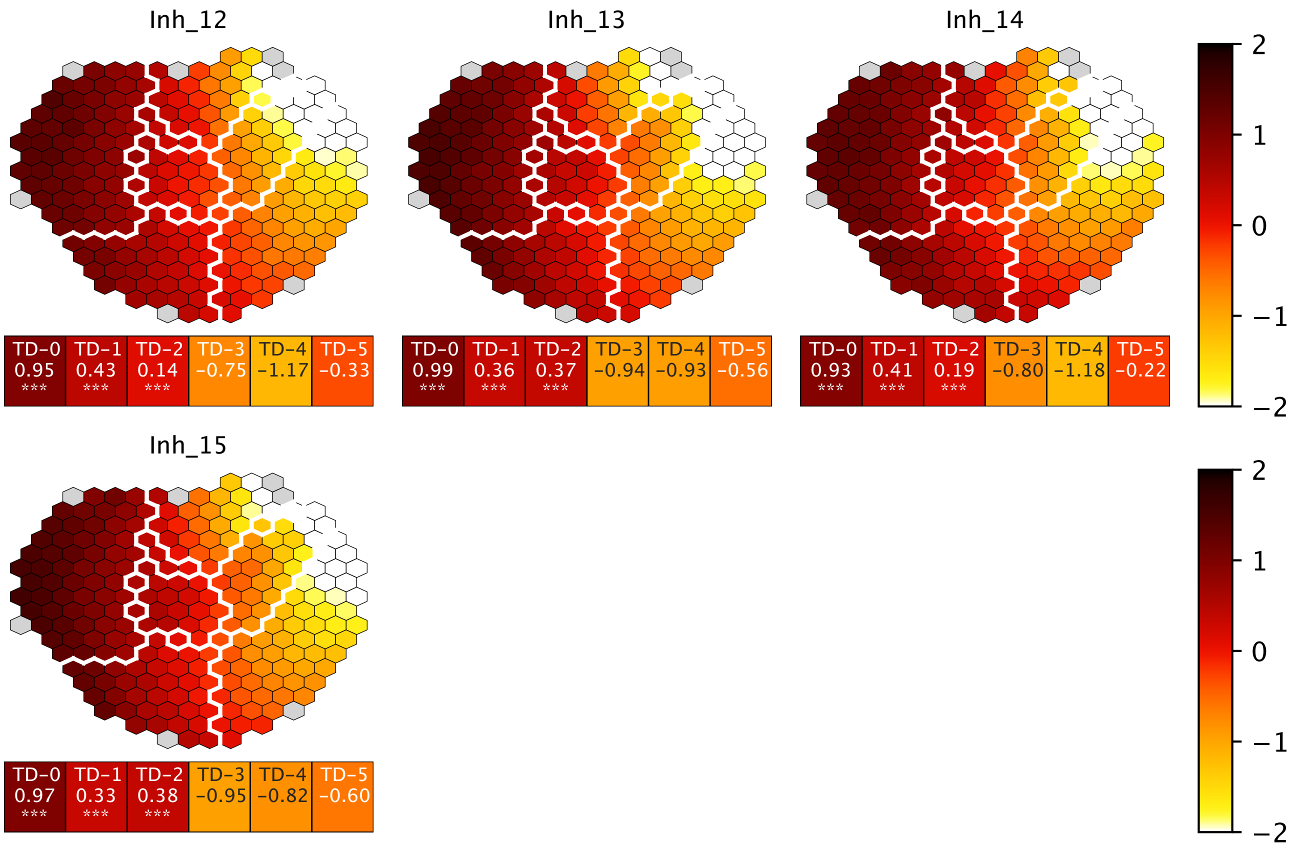

Supplemental Figure S2e: - Tissue Domain Hexbin UMAP representation of microglia

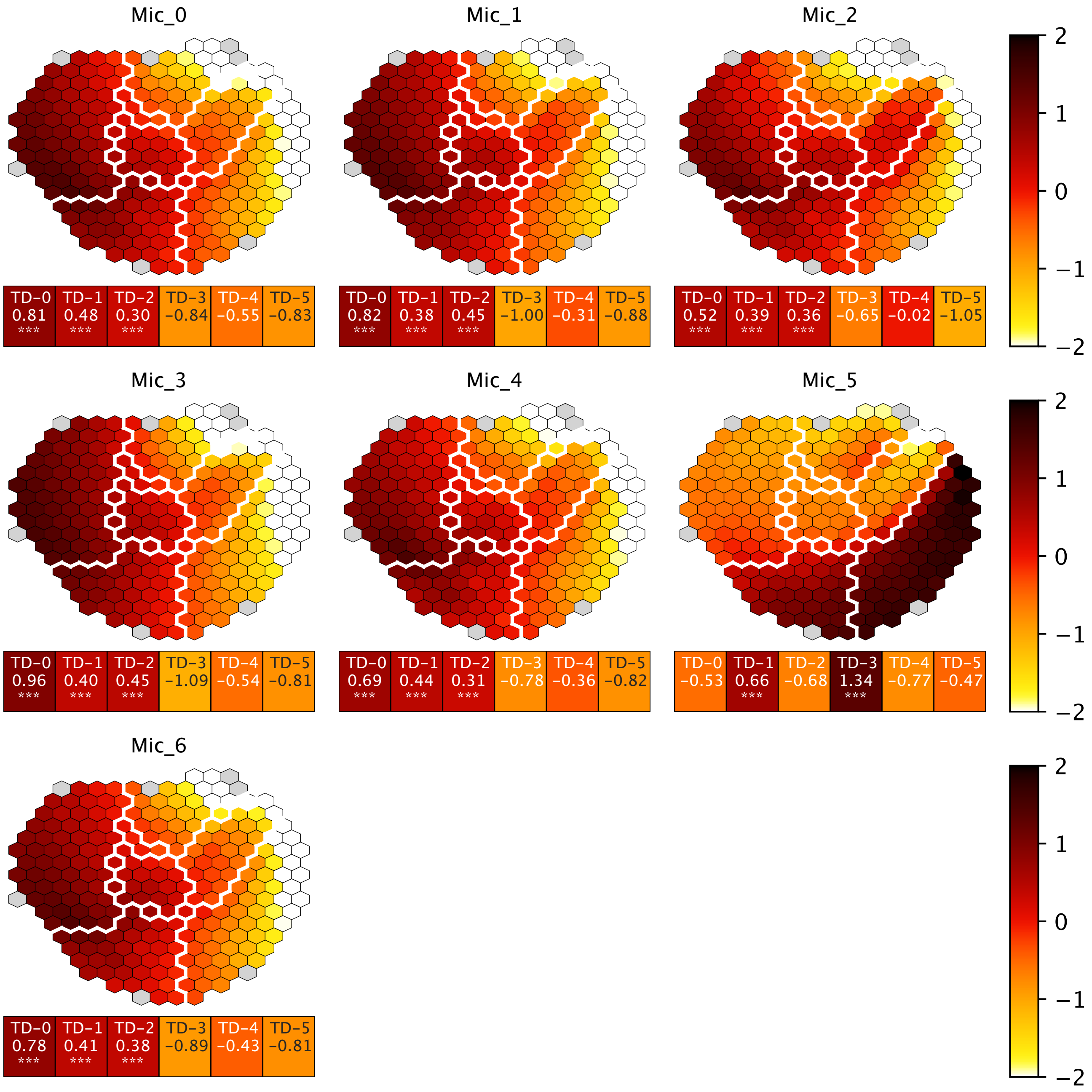

Supplemental Figure S2f: - Tissue Domain Hexbin UMAP representation of oligodendrocytes

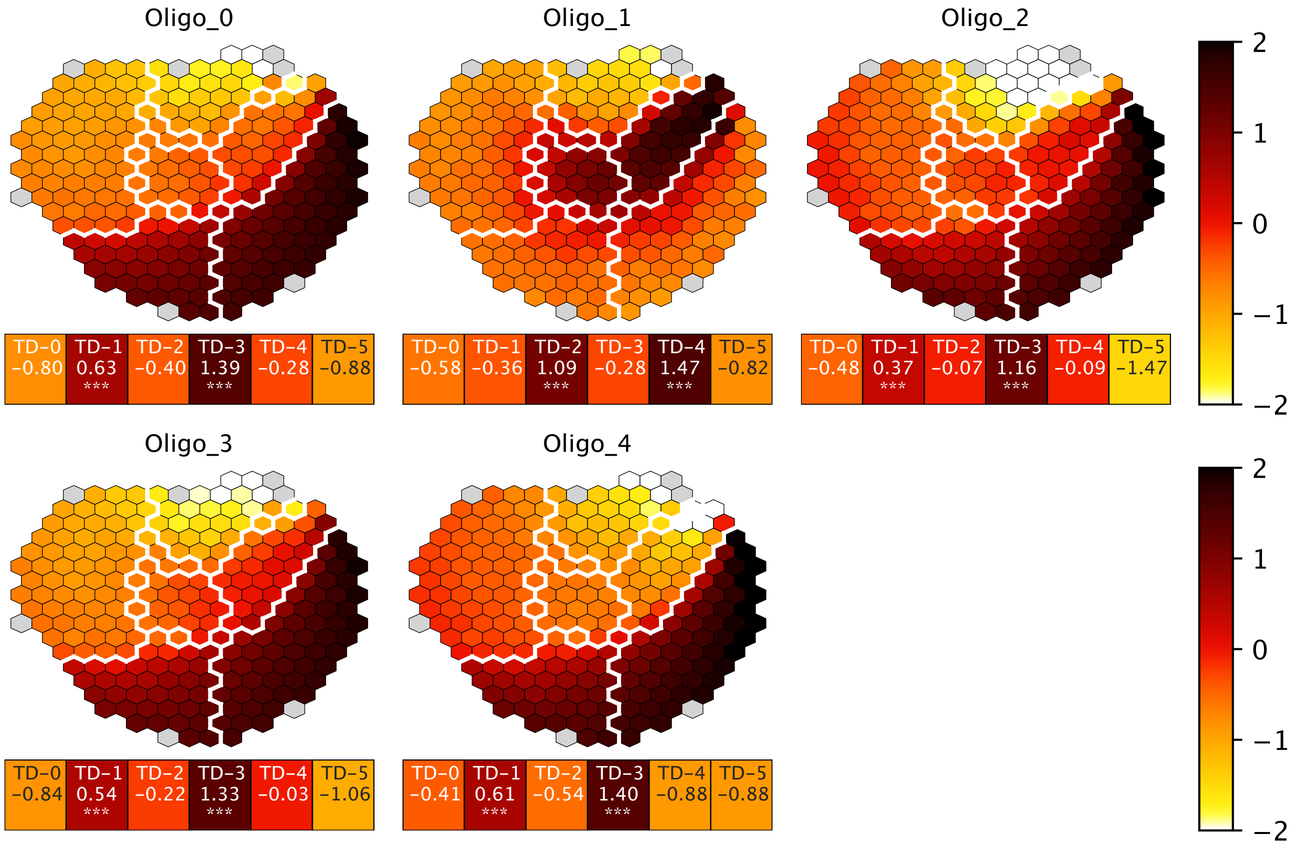

Supplemental Figure S2g: - Tissue Domain Hexbin UMAP representation of OPCs

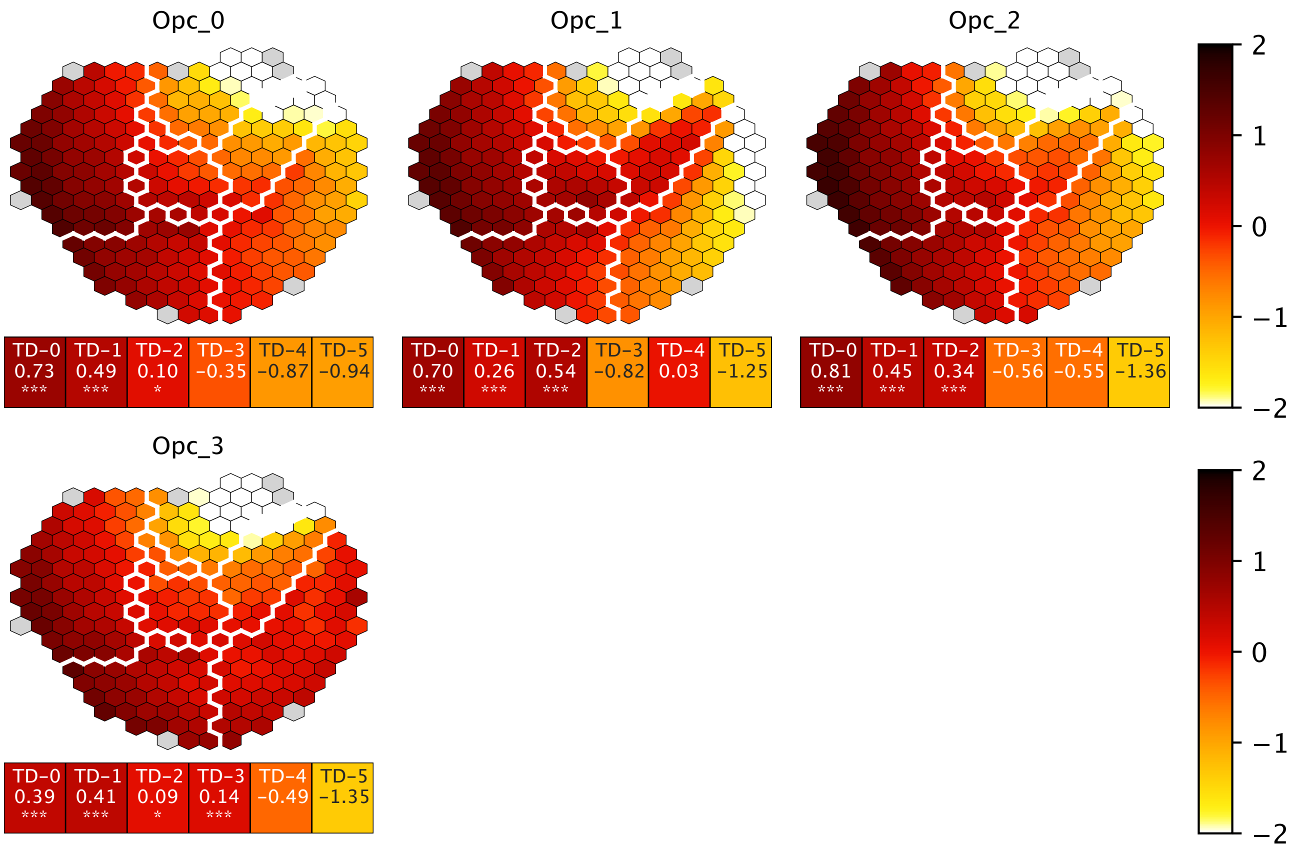

Supplemental data 1: Single Cell Biology

Single nuclei RNA-seq: identification of cellular subpopulations. We sequenced 164,230 single nuclei from sections adjacent to those used for Visium spatial transcriptomics from 24 individuals in the OCT cohort. Nuclei were isolated using the Optiprep density gradient protocol and processed with Chromium Next GEM Single Cell 3' Reagent Kits v3.1 (10X Genomics). After removing 51,532 doublets, we obtained 112,698 high-quality single-nuclei transcriptomic profiles with an average of 3,399 measured genes per nucleus (IQR 1,563-4,663).

Major cell type annotation. Using log-transformed counts of highly variable genes (HVGs) and scVI integration to minimize batch effects (Visium slide ID, RIN, cortical area), we identified six major cell types via Leiden clustering and transcriptomic annotation (Fig 1h & S1a). We annotated these cell types[6]: 27% of the cells were excitatory neurons marked by *NRGN* (30,554 nuclei), 13% were inhibitory neurons (*GAD1* - 15,081 nuclei), 11% were astrocytes (*AQP4* - 12,638 nuclei), 38% were oligodendrocytes (*MBP* - 42,698 nuclei), 5% were microglia (*CSF1R* - 6,039 nuclei,), 5% were oligodendrocyte precursor cells (OPCs, main marker *VCAN* - 5,688 nuclei). The relative proportions of these cell types are consistent with prior studies[6,7].

Spatial mapping of cellular subpopulations. Using Cell2location[8] we mapped cell-type abundances to Visium spots. This analysis reproduced expected laminar patterns (Extended Data Figure 2b-e, Table S8): microglia and astrocytes enriched in layer 1; excitatory and inhibitory neurons across layers 2-6; and oligodendrocytes in deep layers/white matter (Extended Data Figure 2a). Layer-specific predictions were validated using external single nuclei seq datasets[9] and Visium-derived marker genes. Excitatory neuronal subtypes localized to their expected layers (Extended Data Figure 2b-f), for example: Exc_0/2/4/10/14 to L2-3; Exc_1/6/8/9/12/13 to L4-5, and Exc_3/5/7/11 to L6. Xenium analysis confirmed correct cortical layer localization (Fig. 4b).

Characterization of neuronal subpopulations.

We annotated 15 excitatory neuronal subtypes and 16 inhibitory neuronal subtypes according to established transcriptomic references[2,6,9]. (Fig. S1d-S1h).

Excitatory neurons were classified by laminar identity and projection class (intratelencephalic (IT), extratelencephalic (ET), near-projecting (NP) and corticothalamic (CT))[2,6,10] (Fig. S1d-e). We annotated Five L2/3 IT (Exc_0/2/4/10/14), Four L4/5 IT (Exc_1/6/8/12), Two L6 IT (Exc_3/11), One L5 ET (Exc_13), One L5/6 NP (Exc_9), Two L6 CT (Exc_5/7), (Fig. S1d & S1e). Exc_13 (L5 ET) and Exc_1/6/8/12 (L4/5 IT) reflected classical cortico-cortical and subcortical projection identities [10]. Cell-abundance clustering identified OCT-TD5 as enriched for L2/3_IT, L5_IT, and L5_ET neurons, forming a cortico-cortical loop microenvironment.

Otero et al.[1] showed that tangle-bearing neurons express *LAMP5*, *COL5A2*, and *RORB* (L2/3) and *RORB* and *PCP4* (L5). We confirmed that all L2/3 IT neurons in our dataset expressed these markers, while only Exc_1 among L4/5 IT neurons expressed *RORB* and *PCP4* (Fig. S1f).

Inhibitory neurons were classified into MGE-derived (*LHX6*+) and CGE-derived (*ADARB2*+) subtypes. We identified as MGE-derived: three Pvalb (Inh_0/13/14), four Sst (Inh_1/8/10/15), one Chandelier (Inh_9) and as CGE-derived**:** three Vip (Inh_2/4/7), three Lamp5 (Inh_3/6/11), one *SNCG* (Inh_5), one Pax6 (Inh_12) (Fig. S1g & h)[2,6]. Several studies suggest early vulnerability of *SST*+ or *LAMP5*+ inhibitory neurons in AD[1,6,11–13]. Based on SEA-AD [6], Inh_1 corresponds to the vulnerable L2/3 *SST*+ population, and Inh_11 to the vulnerable L1 *LAMP*5+ subtype (Table S5).

Glia populations.
Glia exhibit dynamic, context-dependent transcriptional states[6,14]. We identified seven microglial, seven astrocyte, five oligodendrocyte, and four OPC states (Fig. S1i-p).
Microglia. Mouse AD models show a transition from homeostatic microglia to disease-associated microglia (DAM)[15], but human microglia adopt HLA-enriched, lipid-associated, and cytokine-responsive states[16–18]. Integration with SEA-AD[6] and ROSMAP[17] confirmed correspondence between our OCT microglial states and established human states
Mic_0:
• Aligns with SEA-AD PVM_3 (*GPNMB*+, *PTPRG*+, *CLEC5A*+, *CTSD*+) (Fig 3l, Table S5).
• Represents an early Aβ-associated, transitional state.
• Increased near Aβ plaques but not pTau (Fig. 3m).
Mic_1
• Homeostatic (*CX3CR1*+, *P2RY12*+, Fig. 3l, Table S4).
• Corresponds to SEA-AD PVM_2 (Fig 3l, Table S5).
Mic_2
• Cytokine-responsive (*SPP1*+, *CCL3*+, *CCL4*+, *CLEC7A*+).
• Lipid-associated gene enrichment (*PLA2G4C*, *ADAM9*, *TNFRSF1B*) (Table S4).
• Maps to SEA-AD PVM_4 and CRM states (Fig 3l, Table S5).
• Strongly enriched for late-PIGs but not early-PIGs.
Mic_5
• Ribosomal biogenesis (*FTH1*+, ribosomal subunits).
• Corresponds to RM[18] (Fig 3l, Table S5).
• Enriched for early-PIGs, late-PIGs, and mPIGs, suggesting a late-stage Aβ-induced activation state (Fig. 3m)
• Prominent in TD1 and TD3 (Fig. 2e).
• Represents a terminal pro-inflammatory state, distinct from Mic_2.
Astrocytes. We identified three classical classes: protoplasmic astrocytes (Astro_1/2/4/5), fibrous astrocytes (Astro_0), interlaminar astrocytes (Astro_6). Astro_4 was enriched for glutamatergic synapse genes; Astro_5 for oxidative phosphorylation (Table S7). Astro_5 co-localized with L2/3_IT and L5_IT neurons in OCT-TD5. Astro_0 was most strongly induced along both A- and T-axes and enriched for *GFAP*, *C3*, *SERPINA3*. Interlaminar Astro_6 (layer 1) expressed *GFAP* and formed long descending processes[19]. They appear in TD3 and TD4 (Fig. 2e).
Oligodendrocyte lineage. OPCs progressed from OPC_0/1 → OPC_3 (COP-like) → OPC_2, consistent with known OPC maturation patterns. OLs expressed myelin genes, with Oligo_1 showing reduced *PLP1* and enriched chromatin-remodeling pathways (GO:0006338, Table

S7). Oligodendrocytes were highly sensitive to pathology: Oligo_0/2/3/4 enriched near Aβ plaques (A-axis, Extended Data Figure 5e); Oligo_1 was dominant in pTau+ regions (T-axis, Extended Data Figure 5e), co-enriched with vulnerable L2/3_IT neurons (Exc_4). This suggests potential involvement of oligodendroglia in local vulnerability and tau-associated degeneration.

Supplemental Data 2: Weighted Gene Co-expression Networks (WGCNA)

Weighted gene co-expression network analysis (WGCNA) assumes that genes with correlated expression patterns share biological functions[20]. By clustering genes according to co-expression, WGCNA identifies functional gene modules, each assigned an arbitrary color label by the algorithm. We performed WGCNA independently on Visium spatial transcriptomic data from gray matter Visium spots of the OCT and CEN cohorts. A minimum module size of 30 genes yielded 16 modules in OCT and 20 in CEN.

Cohort-1: Octogenarians (OCT).
We analyzed 17,271 genes across 43,169 gray-matter Visium spots, identifying 16 co-expression modules. Based on Gene Ontology (Table S4) and cell-type enrichments (Extended Data Figure 5a) we conclude that eight modules primarily reflected neuronal biology:
• OCT-Midnightblue was enriched for inhibitory neuron markers; GO analysis identified GABAergic interneuron differentiation and potassium transport
• OCT-Yellow, OCT-Red, and OCT-Magenta were enriched for excitatory neuronal markers;
• OCT-Lightcyan, OCT-Salmon, OCT-Blue, and OCT-Cyan showed mixed excitatory/inhibitory enrichment.
Six modules were predominantly glial:
• OCT-Green-Yellow, OCT-Tan, and OCT-Pink were enriched for microglial markers. GO terms indicated inflammation (OCT-Green-Yellow), vascular/microglial signaling (OCT-Tan), and mitochondrial metabolism (OCT-Pink).
• OCT-Purple was enriched for oligodendrocyte/OPC markers related to myelination.
• OCT-Turquoise was enriched for astrocytic markers (e.g., *APOE*, *CLU*).
• OCT-Black showed mixed astrocytic, microglial, and oligodendrocytic signatures (*GFAP*, *INPP5D*).
Two additional modules (OCT-Green and OCT-Brown) contained mixed cellular signatures. Correlation of OCT module expression with cognitive status (Extended Data Figure 5c) and Tissue Domains (Extended Data Figure 5b) revealed that five modules, OCT-Midnightblue, OCT-Pink, OCT-Red, OCT-Yellow, and OCT-Brown, were preferentially expressed in healthy controls. The remaining modules were enriched in OCT+DEM and OCT–DEM, reflecting diverse responses to Aβ and pTau.
As expected, healthy-control modules were enriched in OCT-TD0. Interestingly, OCT-Brown and OCT-Yellow also appeared in OCT-TD5, suggesting these TDs retain partially preserved neuronal programs. Among AD-related modules:
• OCT-Yellow, OCT-Lightcyan, OCT-Salmon, OCT-Magenta, and OCT-Brown were enriched in OCT-TD0 and OCT-TD5, both associated with Ast_5 and Exc_1/2/13 (Fig. 2e, Extended Data Figure 5b).
• OCT-Cyan, OCT-Blue, and OCT-Purple were enriched in OCT-TD2 and OCT-TD4, microenvironments with Oligo_1, Exc_4 and pTau (Fig. 2e, Extended data Fig. 5b).

• OCT-Green-Yellow, OCT-Tan, OCT-Pink, OCT-Turquoise, OCT-Black were enriched in OCT-TD1 and OCT-TD3, associated with Mic_5, Ast_0/3/6, and Oligo_0/2/3/4. Together, these glial modules highlight inflammation, mitochondrial stress, and vascular–immune signaling as key responses to Aβ and pTau in OCT+DEM brains.

Cohort-2: Centenarians (CEN).

We investigated 18,090 genes across the spatial transcriptomic profiles of 45,701 Visium spots from the gray matter of the CEN cohort and identified 20 modules of co-expressed genes. Interestingly, 19 of these modules show significant associations with one or more networks identified in the OCT cohort (Extended Data Fig. 5g). Based on biological functions (Table S4) and the overlap with the OCT WGCNA modules, we identified 7 neuronal modules (CEN-Lightyellow, CEN-Gray60, CEN-Lightgreen, CEN-Lightcyan, CEN-Red, CEN-Salmon, CEN-Tan) and 9 glial modules (CEN-Yellow, CEN-Black, CEN-Purple, CEN-Pink, CEN-Magenta, CEN-Blue, CEN-Green-Yellow, CEN-Cyan, CEN-Midnightblue).Eight modules show great similarity between the CEN and the OCT cohorts:

OCT-Midnightblue (inhibitory neurons) splits into: CEN-Lightyellow (*RELN*-associated signaling; overlap 16 genes; OR = 177; $p = 5\times10^{-28}$) and CEN-Grey60 (*KCNAB1*, *KCNAB3* and *GAD1*, *GAD2*-associated pathways; overlap 47 genes; OR = 662; $p = 6\times10^{-95}$)

OCT-Salmon showed strongest overlap with CEN-Lightcyan (overlap = 64; OR = 203; $p = 7\times10^{-104}$), corresponding to *KCND2*/*KCNIP2* potassium transport.

OCT-Cyan splits into: CEN-Lightgreen (overlap 33 genes; OR = 377; $p = 3\times10^{-62}$), enriched for *SEMA3*/*EPHA* axon-guidance signaling and CEN-Red (overlap 41 genes; OR = 18.5; $p = 1.5\times10^{-32}$)

These strong overlaps indicate preserved neuronal transcriptional programs across aging cohorts, despite differences in pathology burden.

Oligodendrocyte module OCT-Purple mapped largely to CEN-Black (255/318 shared genes; OR = 241; $p \approx 0$). These genes involved RHO-family GTPase signaling, sphingolipid biosynthesis, *SOX10*-driven myelination, and *PLP1*/*CNP*-mediated myelin maintenance.

The inflammatory microglial module OCT-Green-Yellow overlapped strongly with CEN-Purple (105/172 genes; OR = 105; $p = 2.4\times10^{-173}$), representing *TREM2*/*TYROBP* signaling and the *C1Q* complement pathway. Astroglial genes from OCT-Black redistributed into CEN-Yellow (162 genes; OR = 162; $p = 9.9\times10^{-73}$) and CEN-Green-Yellow (43 genes; OR = 43; $p = 1.9\times10^{-26}$). The large OCT-Blue module splits into CEN-Brown (1,196 genes; OR = 22.4; $p \approx 0$) and CEN-Blue (1,255 genes; OR = 2.17; $p = 2\times10^{-77}$); both enriched for DNA repair, replication stress, and ER stress, consistent with OCT-Blue. Finally, OCT-Pink overlapped with CEN-Magenta (222 genes; OR = 376; $p \approx 0$), representing ribosomal and mitochondrial translation pathways.

Conclusion

Across two independent cohorts representing distinct aging trajectories, we identified eight conserved transcriptional modules, including: *RELN*-related inhibitory neuron signaling (16 genes); *KCNAB*-related potassium transport (47 genes); *KCND2*/*KCNIP2* ion-channel regulation (64 genes); *SEMA3*/*EPHA*-guided axon signaling (33 genes); Myelin-associated

SOX10/*PLP1*/*CNP* pathways (263 genes); Ribosomal/translation pathways (223 genes); Microglial complement/*TREM2* activation (112 genes). Notably, the 112 microglial activation genes were consistently upregulated in response to Aβ and pTau in both cohorts, while *KCND2*/*KCNIP2* potassium-channel genes were consistently downregulated in pTau-rich environments. These results reveal a conserved microglial activation program linked to amyloid pathology and suggest a conserved neuronal vulnerability program associated with tau pathology, across both OCT and CEN brains.

References;

1. Otero-Garcia, M. *et al.* Molecular signatures underlying neurofibrillary tangle susceptibility in Alzheimer's disease. *Neuron* **110**, 2929-2948.e8 (2022).
2. Tasic, B. *et al.* Shared and distinct transcriptomic cell types across neocortical areas. *Nature* **563**, 72–78 (2018).
3. Paolicelli, R. C. *et al.* Microglia states and nomenclature: A field at its crossroads. *Neuron* **110**, 3458–3483 (2022).
4. Sadick, J. S. *et al.* Astrocytes and oligodendrocytes undergo subtype-specific transcriptional changes in Alzheimer's disease. *Neuron* **110**, 1788-1805.e10 (2022).
5. Murdock, M. H. & Tsai, L.-H. Insights into Alzheimer's disease from single-cell genomic approaches. *Nat. Neurosci.* **26**, 181–195 (2023).
6. Gabitto, M. I. *et al.* Integrated multimodal cell atlas of Alzheimer's disease. *Nat. Neurosci.* https://doi.org/10.1038/s41593-024-01774-5 (2024)
7. Mathys, H. *et al.* Single-cell transcriptomic analysis of Alzheimer's disease. *Nature* **570**, 332–337 (2019).
8. Kleshchevnikov, V. *et al.* Cell2location maps fine-grained cell types in spatial transcriptomics. *Nat. Biotechnol.* **40**, 661–671 (2022).
9. Hodge, R. D. *et al.* Conserved cell types with divergent features in human versus mouse cortex. *Nature* **573**, 61–68 (2019).
10. Baker, A. *et al.* Specialized Subpopulations of Deep-Layer Pyramidal Neurons in the Neocortex: Bridging Cellular Properties to Functional Consequences. *J. Neurosci.* **38**, 5441–5455 (2018).
11. Cain, A. *et al.* Multicellular communities are perturbed in the aging human brain and Alzheimer's disease. *Nat. Neurosci.* **26**, 1267–1280 (2023).
12. Mathys, H. *et al.* Single-cell atlas reveals correlates of high cognitive function, dementia, and resilience to Alzheimer's disease pathology. *Cell* **186**, 4365-4385.e27 (2023).
13. Waller, R., Mandeya, M., Viney, E., Simpson, J. E. & Wharton, S. B. Histological characterization of interneurons in Alzheimer's disease reveals a loss of somatostatin interneurons in the temporal cortex. *Neuropathology* **40**, 336–346 (2020).
14. Escartin, C. *et al.* Reactive astrocyte nomenclature, definitions, and future directions. *Nat. Neurosci.* **24**, 312–325 (2021).
15. Keren-Shaul, H. *et al.* A Unique Microglia Type Associated with Restricting Development of Alzheimer's Disease. *Cell* **169**, 1276-1290.e17 (2017).
16. Sun, N. *et al.* Human microglial state dynamics in Alzheimer's disease progression. *Cell* **186**, 4386-4403.e29 (2023).
17. Green, G. S. *et al.* Cellular communities reveal trajectories of brain ageing and Alzheimer's disease. *Nature* **633**, 634–645 (2024).
18. Mancuso, R. *et al.* Xenografted human microglia display diverse transcriptomic states in response to Alzheimer's disease-related amyloid-β pathology. *Nat. Neurosci.* **27**, 886–900 (2024).
19. Falcone, C. *et al.* Cortical Interlaminar Astrocytes Are Generated Prenatally, Mature Postnatally, and Express Unique Markers in Human and Nonhuman Primates. *Cereb. Cortex* **31**, 379–395 (2021).
20. Zhang, B. & Horvath, S. A general framework for weighted gene co-expression network analysis. *Stat. Appl. Genet. Mol. Biol.* **4**, Article17 (2005).
