# Supplementary material for: Human microglial transitions at the Aβ–tau inflection point associate with divergent pathways to dementia and resilience
Source: Nat Med. 2026 Jun 4;32(6):2047–59. doi: 10.1038/s41591-026-04393-8 (PMC13278961; doi:10.1038/s41591-026-04393-8)
Supplement: Supplementary file 2 — Reporting Summary [file 41591_2026_4393_MOESM2_ESM.pdf]

Corresponding author(s): Niels Plath
Mark Fiers
Bart de Strooper

Last updated by author(s): Nov 23, 2025

Reporting Summary

Nature Portfolio wishes to improve the reproducibility of the work that we publish. This form provides structure for consistency and transparency in reporting. For further information on Nature Portfolio policies, see our Editorial Policies and the Editorial Policy Checklist.

Statistics

For all statistical analyses, confirm that the following items are present in the figure legend, table legend, main text, or Methods section.

n/a	Confirmed	
☐	☒	The exact sample size (*n*) for each experimental group/condition, given as a discrete number and unit of measurement
☐	☒	A statement on whether measurements were taken from distinct samples or whether the same sample was measured repeatedly
☐	☒	The statistical test(s) used AND whether they are one- or two-sided<br>*Only common tests should be described solely by name; describe more complex techniques in the Methods section.*
☐	☒	A description of all covariates tested
☐	☒	A description of any assumptions or corrections, such as tests of normality and adjustment for multiple comparisons
☐	☒	A full description of the statistical parameters including central tendency (e.g. means) or other basic estimates (e.g. regression coefficient) AND variation (e.g. standard deviation) or associated estimates of uncertainty (e.g. confidence intervals)
☐	☒	For null hypothesis testing, the test statistic (e.g. *F*, *t*, *r*) with confidence intervals, effect sizes, degrees of freedom and *P* value noted<br>*Give P values as exact values whenever suitable.*
☐	☒	For Bayesian analysis, information on the choice of priors and Markov chain Monte Carlo settings
☐	☒	For hierarchical and complex designs, identification of the appropriate level for tests and full reporting of outcomes
☐	☒	Estimates of effect sizes (e.g. Cohen's *d*, Pearson's *r*), indicating how they were calculated

Our web collection on statistics for biologists contains articles on many of the points above.

Software and code

Policy information about availability of computer code

Data collection	All software used in this study consists of publicly available tools and standard analysis pipelines. Image acquisition and processing were performed using Axio Scan Z1 (Zeiss), NIS-Elements (Nikon Version 3.1), QuPath (0.2.3). Spatial transcriptomics and single-nucleus RNA-seq data were processed using Cell Ranger v6.0.1, Space Ranger v1.2,
Data analysis	All software used in this study consists of publicly available tools and standard analysis pipelines.<br>Spatial transcriptomics was analyzed using Loupe Browser v5.0 (10x Genomics), Xenium Ranger v1.7, Souporcell 2.0, Scanpy 1.8.2, scVI/ scANVI (scvi-tools versiom 1.1.6) Deconvolution and tissue-domain modeling were conducted using Cell2Location (Version 0.1), Harmony (Version 0.06), and Matplotlib (version 3.9.1) for visualization. Differential gene expression and abundance analyses were performed with edgeR(version 3.9.0) , while MAGMA v1.10 was used for GWAS gene aggregation. Additional statistical analyses were carried out in Python using SciPy v1.13.1 and statsmodels v0.14.5. All tools are openly available and referenced in the Methods; no custom software was generated for this study.

For manuscripts utilizing custom algorithms or software that are central to the research but not yet described in published literature, software must be made available to editors and reviewers. We strongly encourage code deposition in a community repository (e.g. GitHub). See the Nature Portfolio guidelines for submitting code & software for further information.

Data

Policy information about availability of data

All manuscripts must include a data availability statement. This statement should provide the following information, where applicable:

- Accession codes, unique identifiers, or web links for publicly available datasets
- A description of any restrictions on data availability
- For clinical datasets or third party data, please ensure that the statement adheres to our policy

Data Availability
Upon publication, all raw sequencing data will be made available on the European Genome-Phenome archive, under control by a data access committee.

Research involving human participants, their data, or biological material

Policy information about studies with human participants or human data. See also policy information about sex, gender (identity/presentation), and sexual orientation and race, ethnicity and racism.

Reporting on sex and gender	The Dutch brain bank provided us with biological sex of the brain samples. Samples were randomly allocated to experimental groups with stratification for known confounding factors, subject to availability constraints. Both sexes were included with balanced representation where feasible; however, sex-specific effects were not statistically evaluated due to inadequate sample sizes for powered subgroup analyses.
Reporting on race, ethnicity, or other socially relevant groupings	No race or ethnicity is reported on. All samples were of Dutch descent.
Population characteristics	We selected two cohorts; the OCT cohort of 24 individuals an age of approximately 80 years old and 20 individuals in the CEN cohort (100+ years old).
Recruitment	We selected a total of 56 individuals from the Netherlands Brain Bank (NBB, Netherlands Institute for Neuroscience, Amsterdam) and from the Dutch 100-plus Study, randomizing for all available potential confounding factors.
Ethics oversight	Ethics Committees of UZ Leuven (Belgium). Informed concent was obtained for all samples. For more details see: https://www.brainbank.nl/about-us/ethics/

Note that full information on the approval of the study protocol must also be provided in the manuscript.

Field-specific reporting

Please select the one below that is the best fit for your research. If you are not sure, read the appropriate sections before making your selection.

☒ Life sciences ☐ Behavioural & social sciences ☐ Ecological, evolutionary & environmental sciences

For a reference copy of the document with all sections, see nature.com/documents/nr-reporting-summary-flat.pdf

Life sciences study design

All studies must disclose on these points even when the disclosure is negative.

Sample size	We based our sample size selection based on the availability of high quality material and sample sizes chosen by (technologically) comparable studies.
Data exclusions	6 samples were very early on excluded from the OCT cohort due to sample quality issues.
Replication	Replication was performed using orthogonal spatial methods: 10x Visium and Xenium spatial transcriptomics; Immunohistochemistry microscopy and comparison to 3rd party data.
Randomization	Allocation in experimental groups was performed based on age, Amyloid beta state, combined with cognitive state for the OCT cohort. The CEN cohort was selected from the Dutch 100+ study, and no further subdivisions were made.
Blinding	Investigators were not blinded during the study.

Reporting for specific materials, systems and methods

We require information from authors about some types of materials, experimental systems and methods used in many studies. Here, indicate whether each material, system or method listed is relevant to your study. If you are not sure if a list item applies to your research, read the appropriate section before selecting a response.

Materials & experimental systems

n/a	Involved in the study
☐	☒ Antibodies
☒	☐ Eukaryotic cell lines
☒	☐ Palaeontology and archaeology
☒	☐ Animals and other organisms
☒	☐ Clinical data
☒	☐ Dual use research of concern
☒	☐ Plants

Methods

n/a	Involved in the study
☒	☐ ChIP-seq
☒	☐ Flow cytometry
☒	☐ MRI-based neuroimaging

Antibodies

Antibodies used

Antibodies for IHC 10X Visium:
4G8 anti-Aβ (mouse, BioLegend 800701/800702; 1:100);
donkey anti-mouse Alexa555 (donkey, ThermoFisher A31570; OCT:1:200, CEN:1:500);
Streptavidin Alexa488 (ThermoFisher S32354; OCT:1:200, CEN:1:300);
NeuN / RBFOX3 Alexa647 (mouse, Novus Biologicals NBP1-92693AF647; 30µg/ml);
AT8-biotin phospho-Tau (mouse, ThermoFisher MN1020B; 1:30)

Antibodies for Immunofluorescence
HLA-DR/DP/DQ (mouse, Abcam ab7856; 1:100);
SPP1 / Osteopontin (rabbit, Merck Millipore HPA027541; 1:200);
IBA1 (goat, Abcam ab5076; 1:200);
CD45-A488 (mouse, BioLegend 304017; 1:50);
AT8-biotin phospho-Tau (mouse, ThermoFisher MN1020B; 1:1000);

Validation

Most antibodies used in this study are commercially available and have been validated by their respective manufacturers, as documented in the technical data sheets accessible on the vendors' websites.
4G8 has been validated for IHC-paraffin on human tissue.
Alexa555-conjugated secondary antibodies, Streptavidin Alexa488, and NeuN/RBFOX3 have been validated for immunofluorescence, IHC, and IHC-paraffin in human samples.
HLA-DR/DP/DQ has been validated for WB, flow cytometry, and IHC-paraffin (human).
SPP1 has been validated for WB and IHC (human).
IBA1 has been validated for WB and IHC-paraffin (human).
CD45-A488 has been validated for flow cytometry (human).
AT8-biotin (MN1020B) has been validated for ELISA and IHC-paraffin (human).

Plants

Seed stocks

Report on the source of all seed stocks or other plant material used. If applicable, state the seed stock centre and catalogue number. If plant specimens were collected from the field, describe the collection location, date and sampling procedures.

Novel plant genotypes

Describe the methods by which all novel plant genotypes were produced. This includes those generated by transgenic approaches, gene editing, chemical/radiation-based mutagenesis and hybridization. For transgenic lines, describe the transformation method, the number of independent lines analyzed and the generation upon which experiments were performed. For gene-edited lines, describe the editor used, the endogenous sequence targeted for editing, the targeting guide RNA sequence (if applicable) and how the editor was applied.

Authentication

Describe any authentication procedures for each seed stock used or novel genotype generated. Describe any experiments used to assess the effect of a mutation and, where applicable, how potential secondary effects (e.g. second site T-DNA insertions, mosiacism, off-target gene editing) were examined.
