# Supplementary material for: Human microglial transitions at the Aβ–tau inflection point associate with divergent pathways to dementia and resilience
Source: Nat Med. 2026 Jun 4;32(6):2047–59. doi: 10.1038/s41591-026-04393-8 (PMC13278961; doi:10.1038/s41591-026-04393-8)
Supplement: Supplementary file 3 — Peer Review File [file 41591_2026_4393_MOESM3_ESM.pdf]

nature portfolio

Peer Review File

Human microglial transitions at the Aβ–Tau inflection point associate with divergent pathways to dementia and resilience

Corresponding Author: Professor Bart De Strooper

This file contains all reviewer reports in order by version, followed by all author rebuttals in order by version.

Version 0:

Reviewer comments:

Reviewer #1

(Remarks to the Author)
In this manuscript, Lu and Chen et al leverage a series of spatial transcriptomic platforms to describe cell fates in Alzheimer's disease (AD). The authors make the following claims: (1) there is a key inflection point marked by a switch from microglial expression of early plaque-induced genes (PIGs) to expression of late PIGs linked to tau pathology; (2) resilience emerges through different immune strategies with octogenarians without dementia lacking late-stage microglial activation despite their despite Aβ burden, and (3) centenarians remaining cognitively-intact and resisted tau pathology despite display of late-stage microglial activation. A major strength of the study and value for the field is the side-by-side comparison of spatial technologies. The manuscript uses unique cohorts that, in general, are understudied: (1) octogenarians with or without dementia and (2) centenarians with advanced amyloid pathology, but limited tau pathology and cognitive decline. A strength of the cohorts is the ability to ask questions about pathologic features with and without dementia. However, because these cohorts are somewhat atypical of the "classic" study design of neuropathology free and AD pathology that we see in ROSMAP and other cohorts, the authors should moderate their conclusions. The manuscript represents an exciting approach to look beyond descriptive features of cell types, cell signatures, and pathology to begin to address hypotheses regarding the transition from amyloid to tau pathology and microglia's role. This is a major strength. However, given that the data is obtained at disease end stage and because we do not have sufficient information to understand the disease trajectory longitudinally of these individuals, the authors should be more cautious in their interpretation. For example, a major conclusion that "there is a key inflection point marked by a switch from microglial expression of early plaque-induced genes (PIGs) to expression of late PIGs linked to tau pathology". Additionally, overall, the manuscript is lacking critical details in the results section that provide context for the study design and approach along with critical QC that is fundamental to the understanding and interpretation of the later findings. After addressing the comments below, the manuscript could be an important one for the field.

Major
1. Authors use terms "Diffuse plaque", "core plaque", "neuritic plaque", and "unclassified plaque"; however, details are lacking in the manuscript regarding how these plaque types were defined. For example, please define antibodies used to identify the plaque types (labeled in figure) and/or the specific criteria used to define these types. The manuscript would benefit from the inclusion of a table that quantifies the distribution of the plaque types within each sample. Overall, the manuscript would be more clear if the authors provide more context as to how these plaque types are defined and how they differ in their contribution to disease pathogenesis – the lack of this description makes it especially difficult to follow along when this pathology is being considered when assessing the TD clusters and their overlap with varying levels of pathology.
2. Define the pTau antibody used in the brains in the results.
3. The cohort labels are misleading. As they are labeled and presented it seems like cohort 1 is discovery and cohort 2 is replication, but really these cohorts are quite different and allow for specific questions. This should be revised in the figures and text.
4. Section: "Spatial transcriptomics reveals distinct pathological niches in octogenarian and centenarian Alzheimer's brains." There is little discussion of the spatial transcriptomics in this section of the results – only how it was done. It seems that the different pathological niches are defined by the immunofluorescence. Please provide more information on the different niches and how spatial transcriptomics is being used to define these niches. The results subsection title should be revised if the niches are defined by immunofluorescence or add more information as to what is considered a pathological niche, and if

this definition is derived from the spatial transcriptomic data, please include more information on how the data was used.
5. How are the pathological niches quantified statistically?
6. Many figures were challenging to interpret. Labels were often lacking or too heavily relying on abbreviations/jargon.
7. The manuscript was very dense and challenging to review. Revising the overall flow would be beneficial. Additionally, consider minimizing abbreviations. For example, the use of TD. In many cases, figures panels are referred to out of order in the text or are never mentioned in the results. This needs to be corrected.
8. The paragraph that begins with "Cluster 0 and 1 represent homeostatic/minimally pathological states…" is incredibly challenging to understand. Of what cells? The following sentence should be expanded as it seems like two ideas: "56% come from OCT_HC (Fig. 2b), showing a wide range of neuronal and glial subtypes (Fig 2d, e)." These challenges are likely a reflection of the spatial transcriptomics field being so new and the analysis methods still evolving. This paper contains impactful data but for it to have a meaningful impact and utility to the field more broadly, there needs to be a clearer breakdown of the analysis steps. The results section needs to more clearly state how the TD clusters were defined and validated. Please provide details regarding how many nuclei contribute to each TD and how the individual brain samples are distributed across the TD. If TD's contain multiple cell-types, this should be explicitly stated. Why was this approach used versus others taken to look at gene expression signatures relative to plaque/pTau proximity. These are important aspects to be added early in the narrative.
9. Results should provide details of the methods and results of the spatial transcriptomics before diving into interesting observations. Basic comparisons and QC should be included in the main figures (particularly Figure 1).
10. One strength of the study is the use of parallel spatial platforms. The authors should provide additional details regarding performance, strengths and limitations as this would massively benefit the field.
11. Fig S1D: It is unclear what comparisons are being made since there are 3 groups being compared for each cortical layer (OCT+DEM, OCT-DEM, and OCT-HC). A Welch's t test was run as opposed to a 1-way ANOVA. Please either re-run the correct statistical analysis for the groups being analyzed or provide more information on what comparisons are being made and remove data that isn't being analyzed from the figure. Please also confirm that the appropriate statistical test is being run on all comparisons being made throughout the manuscript and provide these details in each relevant figure.
12. While this reviewer appreciates that APOE genotypes were matched to the best degree possible in the cohorts and that matching is challenging when postmortem tissue is limited, Fig S1A indicates that the majority of OCT+DEM are APOE34, which there are very few APOE4 carriers among other OCT cohorts and in the CEN. Given this distribution, it remains possible that APOE4 is driving some of the phenotypes reported in the OCT+DEM findings. These should be further tested and discussed in the paper.
13. A lot of data presented in the supplemental figures and tables was important for determination of how to proceed with analyses presented in the main figures. For example, data presented in Supplemental Fig1D led the authors to focus on TDs from cortical layers 3-5. These data should be moved to the main figures.
14. The term "inflection point" is too strong given the cross-sectional nature of the data that is also end stage tissue.
15. Authors should provide more details and context regarding the genes driving the inflection point. This will add to the novelty and impact of the paper and including these findings more prominently in the main figures would be helpful.
16. Given this group's contributions to our understanding of PIGs via spatial in mouse models, the paper would be stronger with experimental illustration of whether the signatures observed in the human brains conserved in mouse model.
17. More data on other cell-types beyond PIGs
18. More representative IHC data generated with the spatial platforms would be helpful to understand and interpret the main findings from the study.
19. Modules are different colors based on the analysis. So a cyan module in one figure is the brown module in another (as an example). While this is a constraint of the packages, the authors could help the reader by providing additional labels for the pathways in the figures that provide more context.
20. Figure 1A: There is no description of what is being stained in the main figure or the figure legend. Please add this information to both.
21. Figure 1B labels should include n's for the first cohort.
22. Please spell out acronyms (e.g., MMSE) prior to use to improve ease of reading, especially for audiences slightly removed from clinical side of the field.
23. Fig 1C: There is no label for what is being stained in the main figure. The figure legend describes abeta and pTau, but there is no mention of what color each was in IF image. Please add this information to the main figure and the figure legend. Additionally, there isn't a clear definition in the figure or the figure legend what the yellow circles are. Based on the context in the results section, these are TDs, but to improve ease of reading, please add this to the figure legend.
24. Fig 1H: Please label what each color is in the main figure and not just the figure legend to improve ease of reading. This panel should also be moved up in the figure to between what is currently 1C and 1D. Finally, a yellow dashed circle is mentioned in the figure legend but not included in the figure. Please add this.
25. Fig S1C and S1D: Please add figure legend with what each color stands for. Otherwise, make the figure legend in S1B larger so it's clearer that it also applies to Fig S1C and S1D
26. "The separation between OCT+DEM and OCT-DEM was primarily driven by an increased number of pTau-positive TDs in the demented group". Use of the phrase "the demented group" may be considered offensive. It's advised to change the phrasing/terminology used to describe this group of individuals for example "group with dementia".
27. It's quite difficult to follow along with the figures as the panels are not all referenced in the text in the order by which they appear in the figures. Please reorganize the figures to match the order in which they are referenced in the text.
28. Fig 1D-G are not referenced in the text. Please add them.
29. "Aβ and pTau pathology concentrated in cortical layers L3–5, affecting 62% of OCT+DEM and 41% of OCT-DEM TDs in these regions (Fig. 2)." It is unclear where this information is presented in Figure 2. Please be more specific about its location.
30. Fig 2B-E: Please label what the color scale is denoting in these panels
31. "Cluster 2 represents low-pathology TDs": What is considered "low pathology"? Please define this.

32. Fig 5E: There are inconsistencies in how cell clusters are referred to in the text vs in the figure (e.g., “Mic_2” vs “Micro_2”). Please make these consistent to ensure ease of reading.
33. SEA-AD atlas and ROSMAP datasets are referenced numerous times in this study, however little context is provided on what they include. Please add context to aid readers unfamiliar with these datasets in understanding what they are and why the similarities you’re observing are important.
34. Please provide more context on what mouse PIGs are in the results section. Some readers may be unfamiliar.
35. “…enriched in such genes including SPI1, APOC1, HLA-DRB1, MS4A6A, MS4A4A, HLA-DRB5, TREM2 and APOC2 (Fig. S5h).” “… and (e) is predominantly expressed in microglia”. Prior to these statements, there is no explanation of why these genes are important or why OCT-Green-Yellow cluster being predominantly expressed in microglia is relevant to the transition between response to abeta pathology   tau pathology. Please provide more context on why microglia are important in the context of AD.
36. “Genes enriched in Mic_2 include SPP1, CCL3, CH25H, and RGS.” Gene names should be italicized.
37. It would be helpful to add a panel like Fig 3I to Figure 3 to show the spatial distribution of the TD clusters (bottom UMAP in Fig 2A). This would make it easier for readers to reference when assessing the data presented in Fig 3D-F.
38. It would be helpful to add a panel in Fig 4 with a representative image of what would be mapped on the A-axis vs T-axis.
39. “Cell type distributions along these axes were analyzed using a rolling window approach (Fig. 4c-d).” Please provide more context on what the rolling window approach is in the results section where it is introduced.
40. Please label the color scales in Figure 5F, G, and I.

Reviewer #2

(Remarks to the Author)
This manuscript examines Alzheimer’s disease (AD) progression and cognitive resilience through spatial transcriptomics and single-nucleus RNA sequencing on brain tissue from octogenarians (with and without dementia) and cognitively intact centenarians with amyloid-β (Aβ) accumulation. Six cellular microenvironments are identified, reflecting AD progression with a transition from Aβ-driven to tau-driven pathology, marked by a shift in microglial states from early inflammatory (early plaque-induced genes, PIGs) to late antigen-presenting (late-PIGs) phenotypes. Resilient individuals exhibit distinct mechanisms: octogenarians without dementia do not induce late-PIGs, while centenarians uncouple late-PIG activation from tau accumulation. Microglial transitions are proposed as potential therapeutic targets. The technical foundation, particularly the use of spatial transcriptomics, is impressive, and the comparison of octogenarian (OCT) and centenarian (CEN) cohorts is ambitious. Yet, the narrative can feel inconsistent, visuals sometimes lack interpretive clarity, and the CEN cohort’s potential, particularly regarding co-pathologies and dementia trajectories, is underexplored. Overall, despite containing exciting methods and cohorts, the manuscript’s results are underwhelming, with limited novel insights.

Major points of critique
- The term “tissue domains” (TDs) is ambiguous, potentially referring to Visium spots, clusters, or another entity, making it difficult to follow without clarification on whether Visium was performed on multiple 10μm sections of 8mm punches.
- Claims about CEN co-pathologies seem overstated without robust testing or controls, which makes it challenging to fully appreciate resilience arguments, especially when compared to a controlled OCT cohort focused solely on Aβ and tau pathologies.
- The CEN cohort’s unique characteristics, such as resilience mechanisms and co-pathologies, feel underexplored, with the assignment of CEN to OCT clusters appearing arbitrary and no clear investigation into dementia trajectories or cell-type-specific signatures.
- Restricting weighted gene co-expression network analysis (WGCNA) to gray matter and focusing on microglia lacks clear rationale, especially given oligodendrocyte associations with tau pathology (e.g., cluster 4); exploring other cell types and justifying white matter exclusion would strengthen the analysis.
- The significance of gene modules (e.g., greenyellow module) feels overstated, as other modules (e.g., black) show similar significance; the method for determining module-pathology specificity (e.g., correlation, group comparison, multivariate modeling) and potential confounding by spatial gradients or layering effects need clearer explanation.
- Despite its technical strengths, the study struggles to deliver novel insights, missing opportunities to fully leverage spatial transcriptomics or the CEN cohort to uncover new aspects of resilience or AD progression.

Minor critiques
- Several figure panels feel redundant (e.g., Fig. 2A/2H) or unnecessary (e.g., Fig. 1D UMAP); merging Figures 1 and 2 could streamline presentation, as Figure 1 focuses on experimental setup while Figure 2 presents biological data.
- Missing or unclear labels (e.g., Fig. 1H lacks protein/color labels; Fig. 3B has swapped tau labels) reduce interpretability; adopting colorblind-friendly palettes (e.g., magenta/green instead of red/green) would improve accessibility.
- Including spatial plots (e.g., q05 abundance, deconvoluted cell type distributions) and hierarchical clustering for heatmaps could enhance clarity; a UMAP labeled by deconvoluted cell types might aid interpretation.
- Figure 2’s color schemes are confusing, with clusters and heatmaps using similar colors without clear correspondence, making it hard to follow.
- The CEN cohort’s biphasic distribution (Fig. S1C) may reflect sex differences or other variables (e.g., TDP-43), but these are not explored, leaving potential insights unaddressed.
- Running WGCNA separately on CEN and assigning CEN to OCT clusters feels arbitrary, potentially overlooking unique CEN clusters.
- Stratifying TDs based on Aβ/tau status without considering neighboring TDs (e.g., A+T- TDs surrounded by T+ TDs) may introduce bias; accounting for spatial context could strengthen the analysis.
- The regression of cortical layers using Harmony and potential layer-specific biases in clusters need clearer explanation.

- Fig. 1H lacks the yellow dashed circle mentioned in the legend, causing confusion.
- Fig. 2D's microglia enrichment in homeostatic/pathology-free clusters seems inconsistent with the microglia-tau pathology link; the significance of plaque/tau overlap in homeostatic clusters (Fig. 2F) is difficult to appreciate without further analysis.
- Fig. 3C's terminology (differential expression vs. gene set enrichment analysis) is inconsistent, and the comparison being performed is unclear.
- Fig. 3G-L's focus on microglia (e.g., mic_5 aligning with late-PIGs) is difficult to fully appreciate, as oligodendrocytes are enriched in tau-associated clusters; imaging data could bolster these findings.
- Fig. 4C/D regression plots may not add significant value and resemble loess plots; oligodendrocyte enrichment near plaques/tau is noted but underexplored.
- Fig. 5's assignment of CENs to OCT clusters feels confusing and lacks clear rationale; focusing on cell-type-specific signatures might be more informative.
- The generalizability of early/late-PIG dynamics to other brain regions (e.g., hippocampal CA1) remains unclear.
- The claim that late-PIGs are uncoupled from tau in CENs is difficult to appreciate, as CENs have less tau pathology overall; evidence linking late-PIGs to tau prevention, rather than co-occurrence with resilience, would strengthen the argument.

Statistical Concerns
- When significance is shown in figures, specifying the statistical test (e.g., Mann-Whitney U, Welch's test) would improve transparency.
- Clarifying whether p-values are raw or adjusted, with adjustments for multiple comparisons as needed, would enhance rigor.
- Specifying whether tests are one-sided or two-sided, particularly for Mann-Whitney U, is necessary for clarity.
- For Cell2Location models (RegressionModel, Cell2location model), specifying the batch variable (e.g., sample ID) or providing a rationale for its absence would improve reproducibility.
- In Extended Data Figure 1D, justifying the use of Welch's test over a paired test for repeated measures, or clarifying that repeated measures do not apply, would strengthen the analysis.
- Hierarchical clustering could improve heatmap clarity, and supplementary UMAPs (single-cell: sample ID; C2L clustering: layer, sample ID) would aid integration visualization.
- Evaluating the value of hierarchical clustering of samples would help assess its contribution to the analysis.
- Including spatial plots of q05 abundance would enhance visualization of deconvolution results.
- Fig. 2E's subtype enrichment quantification and whether Cell2Location used subtypes in the reference need clearer explanation.
- Fig. 4C/D's regression should be evaluated for added value or considered for simplification/removal.
- Reordering comparisons (e.g., A+T- vs. A-T- instead of A-T- vs. A+T-) would improve clarity.

Reviewer #3

(Remarks to the Author)
To investigate infliction points in AD pathology, the authors utilized spatial transcriptomics and single-nucleus RNA sequencing on brain tissue from octogenarians both with and without dementia, as well as from centenarians who exhibited consistent Aβ and tau accumulation. They focused on small tissue domains near Aβ plaques or tau pathology and validated results with Xenium in situ hybridization. Unbiased clustering of the ~60 000 tissue domains (TDs) from 24 individuals revealed six distinct TD clusters associated with pathology and cognitive status. Cluster 0 reflected homeostatic states; Clusters 1–2 were transitional, 3–4 showed advanced inflammation, and 5 indicated late-stage neuronal dysregulation. These clusters outline a spatial trajectory from homeostasis to advanced AD pathology characterized by increasing inflammation and tau accumulation. Focusing on microglia within these TD, they identified an immune transition point where a switch in microglial states, from early inflammatory (early PIGs) to late antigen presenting phenotypes (late PIGs) occurred. The shift from early to late microglia across the TD seems minimal and occurred mostly form cluster 3 to 4. The TD domains from centenarians were comparable to those form the octogenarians. However, octogenarians seemed to avoid late microglial activation, while centenarians tolerated it without progressing to tau pathology.
In summary, this is an interesting approach attempting to determine cellular changes that associate with infliction point such as the conversion to tau pathology.

Major points:
1) Validation is missing. The authors should aim at validating early and late microglia. It is essential to know whether they are able to confirm their bioinformatical analysis using orthogonal approaches. The authors could for example use immunohistochemistry with antibodies against the identified markers to validate these results. This is important as the difference of early and late PIGs shown in Figure 3d and 3e seem to be minimal.
2) The interpretation of the data shown for microglia for centenarians is a bit confusing. In Figure 5 there seems to be no TD differences between plaque and amyloid pathology; and no differences between early and late PIGs. The data does not confirm the data of octogenarians. The authors use this data to claim that centenarians are resilient to late microglia changes without progressing to tau. Another explanation is that advanced age is driver of late microglia. Validation using independent methods is necessary before making these conclusions. Can the authors confirm the disconnect of tau pathology and late microglia in centenarians using immunohistochemistry?
3) How did the authors use proximity to pathology as criteria to classify cells? In 2D this is possible. How, can the authors exclude pathological changes within the depth of the tissue that is not seen in 2D.
4) The authors need to improve the visualization of the data to make the manuscript readable for a wider audience. The main messages are difficult to grasp. For example, the OCT-Green-Yellow module is not presented sufficiently in the figures. The changes in microglial pathways across early and late states are not displayed in the main figures.
5) The question arises whether the changes in PIGs reflect differences in resilience mechanism as the authors claim in the

title and the abstract. A more careful interpretation of the data is required.
6) Microglia have been studied extensively in AD. The authors need to determine whether early and late microglia are detected in other data sets. An attempt is made in Figure 3g but it is not clear from this analysis whether similar transitions correlate with an early and late stage of the disease.

Specific points to the figures:
Figure 2: shows only clustering of TD from cortex 3-5. Could the authors show TD from other brain regions? White matter?
Figure 2c: how was the correlation with pathology carried out? Was plaques or tau pathology recognized by Visium? The definition of plaque and pTau pathology needs to be defined.
The labeling in Figure 2c showing A and T axis is not understandable. Rephrase in the legend.
Figure 2f is not understandable. It looks like %TD with plaques or with pTau is not very different between the TD clusters. However, in 2c there are striking differences. TD0 without pathology in 2c but in 2f with pathology.
The data in 2g and the model in 2f is interesting. Can a trajectory of the associated cellular changes be shown? Separate for microglia, oligodendrocytes and neurons?
Figure 3 is not easy to follow, and more for the specialized reader. For example, 3f the different microglia nomenclature across the 3 reference studies is not easy to grasp. What is the message of Figure 3? It would be more interesting to get more information on genes and pathways that discriminate between early and late microglia. Figure 3c is an attempt to do this but this can be improved.
Figure 3a is not readable. Colors cannot be seen.
Figure 3B mislabeling of positive and negative in the tau axis?
Figure 4 is simply a validation of the Visium data by Xenium data. Are there any additional information using Xenium. The quality of Figure 4a needs to be improved.
Figure 5 there seems to be no TD differences between plaque and amyloid pathology; and no differences between early and late PIGs. What is the main message of this figure?

Version 1:

Reviewer comments:

Reviewer #1

(Remarks to the Author)
The authors have adequately addressed all previous concerns through substantial revisions, including added details in the results section and reorganization of the narrative and figures. These changes improve readability and better emphasize the findings' relevance to the field.

Reviewer #2

(Remarks to the Author)
This manuscript presents a descriptive, multi-omics analysis of AD pathology in understudied cohorts, highlighting microglial roles in resilience. The data quality is commendable, with robust integration of spatial and single-nucleus modalities, orthogonal validation, and careful cohort selection to minimize confounders. However, while the authors propose new terminology, the novelty in methods and concepts is limited. The core findings (microglial state shifts, PIG programs, Aβ-tau transitions) build incrementally on prior work, particularly Mathys et al., Nature, 2019 and Chen et al., Cell, 2020, without transformative insights. Overall, the impact is moderate, as the study remains correlative and lacks mechanistic depth or functional validation, thereby limiting its broader influence in the field. While the resilience angle is intriguing, it feels underexplored, and the emphasis on "inflection points" risks overinterpretation given the static, end-stage nature of the data.

Strengths:
1. The data are high quality and technically rigorous. The integration of Visium (60,129 + 61,739 spots from OCT and CEN), snRNA-seq, and Xenium is well-executed, with strong QC (spot filtering, pathology segmentation, layer-specific deconvolution via Cell2Location). The orthogonal validation across modalities enhances reliability. The continuous pathology quantification is a sensible advance over categorical labels, enabling the nuanced modeling utilized within the manuscript.
2. The OCT (matched, minimal co-pathology) and CEN (resilient centenarians with high Aβ) cohorts are valuable and understudied, allowing side-by-side exploration of dementia vs. resilience in human aging. This addresses a gap in typical AD datasets, which often include broader age ranges and confounders.
3. The rebuttal effectively resolves ambiguities, particularly around terminology and cohort framing, which has improved readability and interpretability. Additions like explicit cautions on longitudinal inferences are important changes.
4. The data could serve as a resource for hypothesis generation, especially on microglial-tau links, and aligns with emerging non-linear AD models.

Critiques:
1. The identification of microglial transitions (early to late PIGs) and tissue domains echoes prior spatial/snRNA-seq studies, such as SEA-AD's cell states and ROSMAP's trajectories. The "inflection point" concept, while framed as novel, is essentially a spatial correlate of established Aβ-tau cascades without new mechanistic revelations. The resilience findings are descriptive and also not groundbreaking. For example, uncoupling late PIGs from tau in centenarians aligns with known

immune adaptations in aging.
2. Claims of "divergent pathways" and therapeutic targets are overstated given the correlative design. This critique remains unaddressed. End-stage data cannot truly resolve temporal dynamics, despite spatial proxies. While the rebuttal acknowledges this caveat, the abstract/main text still implies causality. Phrases like "define divergent pathways" should be altered to "correlate with" to avoid implying causation.
3. Co-pathologies in CEN, like TDP-43 in 13/20 cases, confound resilience interpretations, and the study underplays this. Quantification of how co-pathologies like TDP-43 correlate with late PIG uncoupling in CEN is underexplored.
4. Functional experiments are absent, limiting translational value.
5. While improved post-rebuttal, some details remain sparse. Exact Leiden parameters are not presented, nor are Harmony thresholds. Focus on superior frontal gyrus (layers 3-5) is justified but limits generalizability to the Aβ-tau transitions, especially when regions like the entorhinal cortex and hippocampus contain higher amounts of tau pathology.
6. Statistical power for subgroup comparisons (8 per OCT group) is low, risking type II errors.
7. The resilience discussion feels tacked on, with CEN as a "complementary" cohort rather than deeply integrated.
8. The CD45 staining is labeling lipofuscin and/or dystrophic neurites. The claim that CD45 and MHCII expression is restricted to Aβ plaques and not tau pathology is also misleading. While tau is used in the staining, the images are biased by showing Aβ plaques or regions without tau altogether. Further, brain areas with high tau pathology, such as the entorhinal cortex or hippocampus, are known to contain abundant MHCII positive microglia.
9. It is not clear whether TDs reflect true biological niches or technical artifacts due to batch effects.

Reviewer #3

(Remarks to the Author)
The authors have answered all my issues. I recommend publication of this interesting work.

Open Access This Peer Review File is licensed under a Creative Commons Attribution 4.0 International License, which permits use, sharing, adaptation, distribution and reproduction in any medium or format, as long as you give appropriate credit to the original author(s) and the source, provide a link to the Creative Commons license, and indicate if changes were made.
In cases where reviewers are anonymous, credit should be given to 'Anonymous Referee' and the source.
The images or other third party material in this Peer Review File are included in the article's Creative Commons license, unless indicated otherwise in a credit line to the material. If material is not included in the article's Creative Commons license and your intended use is not permitted by statutory regulation or exceeds the permitted use, you will need to obtain permission directly from the copyright holder.
To view a copy of this license, visit https://creativecommons.org/licenses/by/4.0/

Reviewers' Comments (black) and our rebuttal (blue). Text cited from the revised paper in small print (and red):

Reviewer #1 (Remarks to the Author):

In this manuscript, Lu and Chen et al leverage a series of spatial transcriptomic platforms to describe cell fates in Alzheimer's disease (AD). The authors make the following claims: (1) there is a key inflection point marked by a switch from microglial expression of early plaque-induced genes (PIGs) to expression of late PIGs linked to tau pathology; (2) resilience emerges through different immune strategies with octogenarians without dementia lacking late-stage microglial activation despite their despite Aβ burden, and (3) centenarians remaining cognitively-intact and resisted tau pathology despite display of late-stage microglial activation. A major strength of the study and value for the field is the side-by-side comparison of spatial technologies. The manuscript uses unique cohorts that, in general, are understudied: (1) octogenarians with or without dementia and (2) centenarians with advanced amyloid pathology, but limited tau pathology and cognitive decline. A strength of the cohorts is the ability to ask questions about pathologic features with and without dementia. However, because these cohorts are somewhat atypical of the "classic" study design of neuropathology free and AD pathology that we see in ROSMAP and other cohorts, the authors should moderate their conclusions. The manuscript represents an exciting approach to look beyond descriptive features of cell types, cell signatures, and pathology to begin to address hypotheses regarding the transition from amyloid to tau pathology and microglia's role. This is a major strength. However, given that the data is obtained at disease end stage and because we do not have sufficient information to understand the disease trajectory longitudinally of these individuals, the authors should be more cautious in their interpretation. For example, a major conclusion that "there is a key inflection point marked by a switch from microglial expression of early plaque-induced genes (PIGs) to expression of late PIGs linked to tau pathology". Additionally, overall, the manuscript is lacking critical details in the results section that provide context for the study design and approach along with critical QC that is fundamental to the understanding and interpretation of the later findings. After addressing the comments below, the manuscript could be an important one for the field.

We thank the reviewer for their thoughtful and constructive assessment of our work. We greatly appreciate the recognition of the study's strengths, including the OCT and CEN cohorts, the side-by-side comparison of spatial transcriptomic technologies, and the opportunity these datasets provide to examine resilience and microglial state transitions in an understudied part of the AD spectrum.
In response to the reviewer's concerns we have revised the manuscript in several ways. First, we have clarified that the inflection points we identify are defined in a spatial and pathological sense rather than as longitudinal transitions, and we have added explicit caution regarding interpretation in the absence of true temporal information. Second, we have expanded methodological descriptions throughout the Results and Methods to provide clearer context for the study design, the rationale for our focus on layer 3 to 5, and the

alignment between spatial and single-nucleus datasets. Third, we have added additional QC information, including details of pathological segmentation, Visium spot filtering, and validation across modalities, to aid interpretation of downstream analyses.
We are grateful for the reviewer's comments which helped to strengthen the clarity and framing of the manuscript.

Major

1. Authors use terms "Diffuse plaque", "core plaque", "neuritic plaque", and "unclassified plaque"; however, details are lacking in the manuscript regarding how these plaque types were defined. For example, please define antibodies used to identify the plaque types (labeled in figure) and/or the specific criteria used to define these types. The manuscript would benefit from the inclusion of a table that quantifies the distribution of the plaque types within each sample. Overall, the manuscript would be more clear if the authors provide more context as to how these plaque types are defined and how they differ in their contribution to disease pathogenesis – the lack of this description makes it especially difficult to follow along when this pathology is being considered when assessing the TD clusters and their overlap with varying levels of pathology.
We thank the reviewer for raising this point and apologize for the lack of clarity in the earlier version. As noted, the inconsistency arose from a shift in our analytical strategy while performing the work over the five years since this study was started. Initially, we annotated pathology using classical morphological categories ("diffuse," "core," "neuritic," and "unclassified"). Representative examples are shown in Fig. 1b, their distribution across samples is summarized in Table S1, and the identification criteria are now described in detail in the Methods and Figure 1 Legend, with explicit reference to antibodies and published criteria (we paste the relevant passages from the paper below).
While this describes the observed pathology in the samples in classical terms, we needed for the main analyses to adopt a continuous, quantitative framework based on Aβ and phosphorylated Tau signal intensities which better capture pathology burden than categorical plaque labels (Fig. 1e). This shift was necessary because strict morphological classes excluded many informative tissue domains with Aβ or pTau pathology that did not meet classical criteria (for example, domains containing both Aβ and Tau but lacking neuritic plaque features). We now introduce this quantitative framework explicitly in the first paragraph of the Results and we provide representative examples of Visium spots with increasing Aβ and pTau burden in Figure 1e. Below the relevant passages in the paper (they are also indicated in yellow in the revised manuscript.

Material & Methods - Pathological Features and Scoring

In this context cored plaques were defined as cortical amyloid plaques with a compact, central amyloid core and a halo of diffuse, extracellular Aβ aggregates[59] . In contrast, diffuse plaques were considered as sharply delineated, extracellular Aβ aggregates within the cortical neuropil that did not contain a core of amyloid material[59]. All other plaque types with ill-defined borders or linked to specific anatomical areas such as subpial or white matter Aβ aggregates[59] were considered as unclassified plaques in the context of this study.

Figure legend Fig 1

(b) Representative immunofluorescence images of diffuse - sharply-delineated, extracellular Aβ aggregates within the cortical neuropil that did not contain a core of amyloid material[59]; cored - cortical amyloid plaques with

a compact, central amyloid core and a halo of diffuse, extracellular Aβ aggregates[59]; and neuritic - diffuse or cored plaques that contain pTau-positive dystrophic neurites[59]
(e) **Visium spots reveal a spatial continuum from $A\beta^{-}pTau^{-}$ to $A\beta^{+}pTau^{+}$ pathology**. Representative immunofluorescence images illustrate a progressive continuum of pathology, from spots lacking detectable Aβ and pTau ($A\beta^{-}pTau^{-}$) to those showing combined pathology ($A\beta^{+}pTau^{+}$). Each panel represents one pathology state, with the number of Visium spots per category shown at the top and the bottom of the figure. White circles mark the actual Visium spot boundaries (55 µm diameter). Percentages (A: Aβ; T: pTau) indicate the proportion of spot area occupied by each pathology. Scale bar = 50 µm.
First paragraph of the result section:
Pathology staining, performed on the same tissue section, was quantified within each spot as the percentage overlap between the Visium spot area and segmented regions based on the immunofluorescent signal for Aβ (4G8) and phosphorylated Tau (AT8) (Fig. 1e, Table S1 & S2). This continuous, spatially resolved measure replaced categorical plaque annotations (diffuse, cored, neuritic, see Fig. 1b), allowing quantitative modeling of pathology–transcriptome relationships (Fig. 1e, Table S1 & S2). The Visium transcriptomic analysis was performed on the CEN and the OCT cohort with the same workflow.

2. Define the pTau antibody used in the brains in the results.
We refer the reviewer to Material and Methods: "Tau-morphology across experimental groups" where we wrote in **Tau-morphology across experimental groups.** pTau-pathology, assessed using and antibody against phosphorylated Tau exhibiting the abnormal phosphorylation sites Ser202 and Thr205 (clone: AT8, Pierce, USA, 1/100)[72,73], revealed large qualitative differences across experimental groups.

3. The cohort labels are misleading. As they are labeled and presented it seems like cohort 1 is discovery and cohort 2 is replication, but really these cohorts are quite different and allow for specific questions. This should be revised in the figures and text.
We thank the reviewer for highlighting this point. We agree that the original cohort labels could inadvertently suggest a discovery–replication structure. The OCT and CEN cohorts were instead selected to address complementary biological questions: the OCT cohort to capture the classical Aβ–Tau disease continuum, and the CEN cohort to interrogate resilience in advanced aging. We have revised text and figure legends throughout to make this distinction explicit. These changes clarify that the two cohorts address distinct biological questions rather than representing a discovery–replication design. Some <u>examples</u> of revisions include:
Introduction: To overcome these limitations, we selected 24 well-characterized octogenarian brains (OCT cohort) matched for age, sex, and APOE status, and free of major non-AD lesions, and a complementary second cohort of 20 centenarian brains from the Dutch 100-plus Study[44–46], spanning preserved to impaired cognition.
Results, first paragraph: To examine resilience mechanisms in advanced aging, we analyzed a complementary cohort of 20 centenarian brains (CEN) from the Dutch 100-plus Study[6,45,47].
Figure 1a legend: The OCT cohort - designed to dissect cellular responses associated with AD pathology and cognitive decline - includes 24 individuals: 8 healthy controls (OCT_HC), 8 with AD pathology but no dementia (OCT-DEM), and 8 with both pathology and dementia (OCT+DEM). The CEN cohort - included to explore mechanisms underlying cognitive resilience in advanced aging – comprises 20 centenarians (CEN) with high Aβ-burden and variable co-pathologies.

4. Section: "Spatial transcriptomics reveals distinct pathological niches in octogenarian and centenarian Alzheimer's brains." There is little discussion of the spatial transcriptomics in this section of the results – only how it was done. It seems that the different pathological niches

are defined by the immunofluorescence. Please provide more information on the different niches and how spatial transcriptomics is being used to define these niches. The results subsection title should be revised if the niches are defined by immunofluorescence or add more information as to what is considered a pathological niche, and if this definition is derived from the spatial transcriptomic data, please include more information on how the data was used.

We thank the reviewer for this valuable comment and agree that the original description did not sufficiently explain how spatial transcriptomics defines the pathological environments in our study. We have made several revisions to address this.

First, we refined terminology throughout the manuscript. In the previous version the term "*Tissue Domains*" was not consistently distinguished from individual "*Visium Spots*" which caused ambiguity. In line with established usage in the field [e.g. PMID: 27365449, 37758947, 32637622], we now refer to the 55µm capture areas as "*Visium spots*" and reserve "*Tissue Domains (TDs)*" for transcriptionally defined clusters of Visium spots that share similar cellular composition or gene expression profiles.

Second, we revised section titles to better reflect the actual analytical workflow. These changes clarify that the TDs are derived from spatial transcriptomic data rather than predefined immunofluorescence categories.

Third, we expanded the relevant Results text to clarify how spatial transcriptomics defines the TDs.

To deconvolve cell composition within Visium spots, we applied Cell2Location[48] confirming layer specific enrichment of excitatory neurons (Fig. S3, Supplemental data A). Harmony[49] was then used to correct for layer effects, followed by unbiased Leiden clustering which identified 6 distinct clusters of Visium spots, termed Tissue Domains (TDs: OCT-TD0 to OCT-TD5, Fig. 2a), each representing a microenvironment with a characteristic cellular composition (Fig. 2b-e). Pathological relevance was inferred a posteriori by correlating TD composition with the OCT cohorts (Fig. 2b) and local Aβ and pTau burden (Fig. 2c), rather than used as input for clustering

Finally, in the first Results paragraph we clarified that pathology is modeled using continuous, spatially resolved quantification of Aβ and pTau signal rather than categorical plaque types, which provides the framework for linking spatial transcriptomes to local pathology.

We believe these changes now clearly explain how spatial transcriptomics, rather than immunofluorescence categorization, defines the pathological tissue domains used throughout the manuscript

5. How are the pathological niches quantified statistically?

We thank the reviewer for this question. Tissue domains (previously referred to as "pathological niches") are defined using Leiden clustering [PMID: 30914743], as implemented in *Scanpy* [PMID: 29409532]. This graph-based method constructs a k-nearest neighbor graph from Visium spots based on their inferred cell type abundance profiles and

partitions the graph into robust communities. These communities correspond to Visium spot clusters that share similar cellular composition. We now refer to these clusters as *Tissue Domains (TDs)*, in line with conventions in the spatial transcriptomics literature.

The pathological relevance of each TD was quantified using two complementary statistical approaches. First, we tested whether TDs were enriched in specific octogenarian diagnostic groups (OCT_HC, OCT–DEM, and OCT+DEM) using one-sided Mann–Whitney U tests and Bonferroni correction (Figure 2b). Second, we examined how much each Visium spot overlapped with Aβ- or pTau-immunostaining (Figure 2c) again using one-sided Mann Whitney U tests comparing all spots within a TD to all other spots. These analyses quantify how each TD relates to local pathology burden and to clinical group.

To ensure that this workflow is fully transparent, we added the following clarification to the Methods section:

Tissue Domain clustering and visualization We performed clustering on the cell2location cell type abundance matrix. We first normalized these abundances to z-scores based on the total cell abundance per Visium spot. We then used the mid-layer (layer 3, 4 and 5) Visium spots of our 24 OCT subjects. To mitigate layer-specific variations, we apply Harmony[49] integration on the layer. The same procedure was used for the 20 CEN subjects. Subsequently, Leiden clustering is applied to cluster these Visium spots, only based on cell abundance, into six distinct clusters, termed "Tissue Domains" for the OCT (named Octogenarian Tissue Domain OCT-TD0 to OCT-TD5) cohort and seven Tissue Domains for the CEN cohort (named Centenarian Tissue Domain CEN-TD0 to CEN-TD6).

6. Many figures were challenging to interpret. Labels were often lacking or too heavily relying on abbreviations/jargon.
We thank the reviewer for this helpful observation. We have thoroughly revised all main and supplemental figures to improve clarity. Specifically, we have expanded or replaced abbreviations in axis labels and legends, added missing labels for anatomical regions, TD identities, and pathology axes, increased font sizes, and standardized color scales across figures. Several panels were restructured to reduce visual complexity, and all legends were rewritten for clarity and consistency. We believe these changes substantially improve figure interpretability.

7. The manuscript was very dense and challenging to review. Revising the overall flow would be beneficial. Additionally, consider minimizing abbreviations. For example, the use of TD. In many cases, figures panels are referred to out of order in the text or are never mentioned in the results. This needs to be corrected.

We thank the reviewer for this important comment and fully agree that improving readability and flow was essential. We have now substantially revised the manuscript to address these concerns.
First, we reorganized the Results section to establish a clearer narrative progression (cohort description → spatial architecture → WGCNA modules → early/late PIG inflection point → microglial states → validation → centenarian comparison). Subsection titles were rewritten to match their content and improve navigability.
Second, we reduced the use of abbreviations wherever possible.

Third, we systematically reviewed all figure references in the text. Panels are now cited in logical order, and each displayed panel is explicitly referenced at least once in the Results. Several figure legends were expanded to ensure they stand alone without requiring cross-referencing.
Together, these changes streamline the flow of the manuscript and significantly improve clarity.

8. The paragraph that begins with “Cluster 0 and 1 represent homeostatic/minimally pathological states…” is incredibly challenging to understand. Of what cells? The following sentence should be expanded as it seems like two ideas: “56% come from OCT_HC (Fig. 2b), showing a wide range of neuronal and glial subtypes (Fig 2d, e).” These challenges are likely a reflection of the spatial transcriptomics field being so new and the analysis methods still evolving. This paper contains impactful data but for it to have a meaningful impact and utility to the field more broadly, there needs to be a clearer breakdown of the analysis steps. The results section needs to more clearly state how the TD clusters were defined and validated. Please provide details regarding how many nuclei contribute to each TD and how the individual brain samples are distributed across the TD. If TD’s contain multiple cell-types, this should be explicitly stated. Why was this approach used versus others taken to look at gene expression signatures relative to plaque/pTau proximity. These are important aspects to be added early in the narrative.
We very much appreciate this critique and agree that the original paragraph was difficult to follow. In response, we have substantially rewritten the relevant section to clarify (i) how Tissue Domains were defined, (ii) how spatial transcriptomics, cell-type deconvolution, and pathology measurements were integrated, and (iii) how TDs relate to individual brains and cellular compositions. The original text beginning “Cluster 0 and 1 represent homeostatic/minimally pathological states…” is now replaced and now reads:

TD0 and TD1 represent homeostatic environments with minimal pathology, comprising 56% of spots from healthy controls (OCT_HC) and containing a broad diversity of neuronal and glial subtypes. TD2 corresponds to a transitional environment where the earliest cellular responses to pathology appear; it is evenly represented across the cohort (Fig. S4) and enriched in the Exc_4 excitatory neuronal subtype and Oligo_1 oligodendrocytes subtype (Fig. 2e & S5). TD3 and TD5 were both strongly associated with Aβ-plaques but differed in their cellular composition. TD3 was enriched in reactive glia and oligodendrocytes (Supplemental data A, Fig. S6) consistent with an early gliotic and myelination-related response surrounding plaques. TD5, in contrast, was dominated by excitatory neurons and astrocytes showing transcriptional enrichment for oxidative phosphorylation and mitochondrial metabolism pathways, indicating elevated energy metabolism rather than overt stress signaling. TD4 represented a distinct, pTau-dominated environment (often co-occurring with Aβ), enriched in DAM-like microglia (Mic_2), reactive astrocytes, Oligo_1 and Exc_4 neurons, partly overlapping with vulnerable neuronal populations described in the SEA-AD atlas[34] (Table S5). Among the microglial states identified in our snRNA-seq dataset (Fig. S2i), Mic_1 and Mic_3 represent homeostatic states, Mic_5 reflects a ribosomal-biogenesis activation state and Mic_2 corresponds to a disease-associated inflammatory state; full characterization of these states is presented in the microglial transition section below (Fig 3l, S6a & Table S7). All Tissue Domains, ranging from low to high pathology, are observed across each of the three cohorts (Fig. S4)

We further explain with much more clarity how the TDs were statistically defined in the manuscript, and see, please, point 4 & 5 of this rebuttal. While TDs are derived from Visium spots, not nuclei, the reviewer’s concern about sample distribution is very important. To

address the this concern, we have improved upon the original Fig. 2f, (which is now Fig. S4), and provide an overview of how the number of Visium spots per TDs distribute across the cohorts (OCT_HC, OCT-DEM & OCT+DEM) in Fig. S4a; how our continuous pathological measurements distribute across TD and cohort (Fig. S4b-e); and how the Visium spots distribute across the TDs per individual sample (Fig. S4f).

To explain why percentage overlap was chosen over proximity, we intentionally adopted a continuous per-spot quantification of pathology early in the analysis (Fig 1e). As outlined in the manuscript (first section of results). Traditional plaque classification (cored, diffuse, neuritic) failed to capture a substantial fraction of signal that did not adhere to those categories, whereas continuously measuring pathology burden per spot enabled quantitative modeling of AD pathology.

Overlap was specifically preferred because proximity alone does not reflect plaque density or intensity: two spots overlapping a staining region may differ profoundly in pathology if one overlaps a dense deposit and the other near a faint, diffuse signal. Percentage overlap directly captures this quantitative variation, proximity much less. Hence we believe it provides a more biologically meaningful representation of local pathological load, which is particularly important when modeling transcriptional responses to graded pathology. This strategy is further supported by our prior spatial transcriptomics study in mouse AD models (PMID: 32702314), where overlap-based quantification proved highly effective.

The revised manuscript now clarifies this rationale more explicitly.

9. Results should provide details of the methods and results of the spatial transcriptomics before diving into interesting observations. Basic comparisons and QC should be included in the main figures (particularly Figure 1).
We thank the reviewer for this valuable suggestion. We have revised the Results to present the spatial transcriptomics framework, pathology quantification, and key QC steps clearly at the beginning, before introducing any biological interpretation.

The Results section now begins with a concise introduction of the spatial transcriptomics workflow, summarizing Visium sampling, cortical layering, alignment of Aβ and pTau immunofluorescence, quantification of pathology by percent overlap per spot, and the introduction of the continuous A- and T-axis framework.
Figure 1 and S1 now includes basic spatial QC, showing pathology distributions across layers, cohort-level summaries, and representative Visium spots spanning the full pathology range.
Before introducing Tissue Domains, we now clearly describe the number of Visium spots, gene detection statistics, Cell2location-based cell-type deconvolution, rationale for focusing on cortical layers 3–5, and consistency of pathology burden across individuals.
Finally, QC elements previously only in the supplement (pathology distributions, layer enrichment, snRNA-seq annotation QC, spatial alignment consistency) are now directly referenced in the main text to better support data quality and methodological transparency.

This anchors the main analysis in well-presented QC information. Together, these revisions clarify the methodological basis of the spatial transcriptomics analysis and provide the essential QC context required for interpreting subsequent findings.

10. One strength of the study is the use of parallel spatial platforms. The authors should provide additional details regarding performance, strengths and limitations as this would massively benefit the field.
We thank the reviewer for highlighting the strength of combining parallel spatial transcriptomics platforms. Our intention in using both Visium and Xenium was to leverage their complementary properties: Visium provides broad transcriptomic coverage across large tissue areas, while Xenium offers high-resolution, hybridization-based spatial localization with single-molecule sensitivity. Although detailed benchmarking of spatial technologies is beyond the scope of this biologically focused manuscript, we agree that clarifying how these platforms complement each other will benefit readers. We have therefore added a concise description to the Results and Methods summarizing the strengths and limitations most relevant to our study design, with appropriate citations to comprehensive comparative reviews [PMID: 36192637; 38965443; 36990426] for readers seeking technical depth. Detailed benchmarking of spatial technologies is beyond the scope of this manuscript. Our results could be interpreted as evidence for the power of the platforms we used. Most experiments are at least a year old due to the time it takes to analyse the data and write the manuscript - and so we are hesitant to make strong statements about suitability of our platforms in the fast-moving field of spatial transcriptomics. There are however sufficient reviews published [PMID: 36192637; 38965443; 36990426] that provide much more comprehensive overviews of the field.

11. Fig S1D: It is unclear what comparisons are being made since there are 3 groups being compared for each cortical layer (OCT+DEM, OCT-DEM, and OCT-HC). A Welch's t test was run as opposed to a 1-way ANOVA. Please either re-run the correct statistical analysis for the groups being analyzed or provide more information on what comparisons are being made and remove data that isn't being analyzed from the figure. Please also confirm that the appropriate statistical test is being run on all comparisons being made throughout the manuscript and provide these details in each relevant figure.
We thank the reviewer for drawing attention to this issue. Following figure reorganization, the former Fig S1D is now represented in Fig. 1c and Fig S1b. We agree that Welch's t-test was not appropriate for comparisons involving more than two phenotype groups and is not optimal for non-parametrically distributed data. We have therefore reanalyzed all pathology comparisons using a nonparametric ANOVA that correctly accommodates the three OCT groups (OCT+DEM, OCT−DEM, OCT-HC) and the centenarian cohort (CEN), stratified by cortical layer and not assuming normally distribution.
In the revised analysis, we tested whether pathology levels differed between phenotype groups within each cortical layer for all five pathological metrics (Aβ plaques, pTau, Cored plaques, neuriotic plaques, and diffuse plaques) (Fig. 1c & S1b). All p-values were Bonferroni-corrected for multiple testing. Figures and legends have been updated

accordingly, and all statistical tests throughout the manuscript have been reviewed to ensure consistency and correctness. The *Methods now include the following clarification:*

Pathology differences across cortical layers. *To examine the distribution of pathology load across cortical layers (Fig. 1c), Aβ and pTau levels were expressed as log-transformed mean percentages per cortical layer and donor. For each pathological marker, values were averaged per donor and per layer. Differences between phenotype groups (OCT+DEM, OCT−DEM and CEN) within layers were tested using a permutation-based ANOVA. Under the null hypothesis of no group effect, phenotype labels were permuted within each layer while preserving donor-level pathology distributions (5,000 permutations). For each permutation, a between-group test statistic was recomputed and compared to the observed statistic to obtain an empirical P-value. P-values were Bonferroni-corrected across cortical layers (α = 0.05).*

Cell-type enrichment within TD clusters. *To assess cellular enrichment within TDs (Figs. 2d and 2e), cell-type and subtype abundances were estimated using Cell2location. Expected values under independence were calculated from group means (mean abundance per TD and per cell type). Enrichment was quantified as* log_2*(observed / expected). One-sided P-values for over-enrichment were derived from standardized residuals and corrected for multiple testing using the Bonferroni method.*

These revisions ensure that the reported comparisons and statistical inferences fully reflect the structure of the data.

12. While this reviewer appreciates that APOE genotypes were matched to the best degree possible in the cohorts and that matching is challenging when postmortem tissue is limited, Fig S1A indicates that the majority of OCT+DEM are APOE34, which there are very few APOE4 carriers among other OCT cohorts and in the CEN. Given this distribution, it remains possible that APOE4 is driving some of the phenotypes reported in the OCT+DEM findings. These should be further tested and discussed in the paper.

We thank the reviewer for this thoughtful comment. We agree that the APOE genotype is an important modifier of microglial and glial responses, and that the relative enrichment of APOE ε4 carriers in the OCT+DEM group raises the possibility that some observed phenotypes could be influenced by genotype. Because of the limited cohort size, however, we do not have sufficient statistical power to dissect APOE-specific effects with confidence. To address this concern, we verified that the key microglia trajectories and co-expression module patterns are consistent across individuals within each phenotypic group and across pathology-defined tissue domains, independent of APOE status. These include the transition from early to late PIG programs and the emergence of TD3 and TD4 as the main pathology-associated environments. This supports that the core findings reflect pathology-associated rather than genotype-driven effects.

We have added the following statement in the Discussion to acknowledge this limitation:

The large number of spatially resolved Visium spots spanning a broad continuum of pathology, provides high confidence in the observed cellular transitions. The inflection points we describe are spatial–pathological rather than longitudinal, yet their coherence across tens of thousands of tissue domains indicates that they represent robust biological checkpoints. Although cohort size limits individual-level analyses such as APOE genotype effects, and despite the higher co-pathology burden in CEN brains, the reproducibility of microglial transition across both cohorts suggests a shared underlying trajectory of AD progression

And

Sample size limited individual-level analyses such as APOE genotype effects, and CEN brains contained higher levels of co-pathology, although these factors did not obscure the shared microglial transitions observed across cohorts.

13. A lot of data presented in the supplemental figures and tables was important for determination of how to proceed with analyses presented in the main figures. For example, data presented in Supplemental Fig1D led the authors to focus on TDs from cortical layers 3-5. These data should be moved to the main figures.

We thank the reviewer for this helpful suggestion and agree that several elements originally included in the supplementary figures are essential for understanding the rationale behind our analytical design. In the revised manuscript, we have therefore reorganized the figures and moved key information, including the distribution of Aβ and pTau pathology across cortical layers (previously Supplemental Figure 1D), into the main Fig. 1c. Presenting these data upfront provides a clear justification for focusing subsequent analyses on tissue domains located in cortical layers 3–5, where pathology is most abundant and informative. To highlight this rationale in the main text, we added the following sentence to the Results section “Six distinct Tissue Domains define AD progression in octogenarian brains”:
Because white matter content was highly variable across samples due to differences in dissection depth, quantitative analyses were restricted to gray matter. Within the gray matter, layers 3–5 showed the highest pTau accumulation and strongest Aβ overlap across individuals, identifying them as the most informative region to study the Aβ–Tau interface (Fig. 1c, S1b).

14. The term “inflection point” is too strong given the cross-sectional nature of the data that is also end stage tissue.
We appreciate the reviewer’s caution regarding the term “inflection point.” We agree that our study is cross-sectional, and that the tissue represents advanced disease stages. To address this, we now explicitly qualify the term as referring to a spatial–pathological, rather than temporal, transition.
The large number of spatially resolved tissue domains (over 60,000 in the OCT cohort alone) spans a wide continuum of local pathology within the same brain region, ranging from pathology-free spots to Aβ-only to Aβ+pTau co-pathology. This spatial heterogeneity enables the reconstruction of distinct stages of cellular change and allows to identify points at which microglial states shift sharply in association with the transition from Aβ- to Tau-dominated pathology.
To avoid any implication of longitudinal inference, we have revised the manuscript text to clarify this interpretation wherever the term “inflection point” appears.
Finally, the spatial positioning of the HLA-enriched microglial state we observe is consistent with independent findings from Mancuso *et al.* (2024) and with ongoing work from our group, both of which place antigen-presenting microglia late in the pathological cascade. This independent convergence supports the interpretation that the spatial–pathological transition we describe corresponds to a late-stage shift in microglial activation.

15. Authors should provide more details and context regarding the genes driving the inflection point. This will add to the novelty and impact of the paper and including these findings more prominently in the main figures would be helpful.
We thank the reviewer for this valuable suggestion. We fully agree that making the genes driving the transition more explicit improves both clarity and impact. In the revised version, we have included the complete lists of early- and late-plaque-induced genes (PIGs) in Table

S3 together with cross-references in the Results section. These lists highlight the functional shift between the two states, including complement and TREM2 associated signaling (SKY, FYB1, HCK; BLNK; FC receptors, SIGLECs) among the early-PIGs, and antigen presentation and cytokine related genes among the late-PIGs (HLA-DRA/DPA1/DPB1/DMA/DMB/DOA, HLA-C, CD74). Finally, we have clarified in the figure legends where representative early- and late-PIGs are visualized, ensuring that the gene-level drivers of the transition are now more prominently integrated into the main narrative.

16. Given this group’s contributions to our understanding of PIGs via spatial in mouse models, the paper would be stronger with experimental illustration of whether the signatures observed in the human brains conserved in mouse model.
We thank the reviewer for this excellent suggestion. We agree that demonstrating conservation of plaque-induced gene signatures across species reinforces the biological relevance of the findings. In response, we have now reintroduced the mouse PIG gene set into Figure 3m, showing that these genes map predominantly to microglial states and align closely with the human early-PIG program.
In addition, we clarified in the Results section *“Neuroinflammatory signatures defined by co-expression modules”* that the mouse PIGs are derived from our previous spatial transcriptomic work in mouse Aβ model brains.
TREM2-related signaling genes (Table S3) and showed strong overlap with the Plaque Induced Genes identified in mouse models (mouse-PIGs; log OR: 4.06, $p = 2.2 \cdot 10^{-16}$)[23], highlighting a conserved innate immune response to Aβ across species.
The statistical comparison shows a robust overlap between the human OCT-Green-Yellow module and the mouse PIG gene set, reinforcing the cross-species conservation of the early microglial response.

17. More data on other cell-types beyond PIGs
We thank the reviewer for this comment and agree that our dataset contains extensive information on many cell types beyond microglia. Detailed analyses of astrocytic, oligodendroglial, and neuronal states are provided in Supplemental Data A and B, including subtype definitions, layer mapping, GO enrichment, and WGCNA module associations. These results demonstrate that multiple glial and neuronal programs change with pathology, but the strongest and most reproducible transitions are microglial and define the Aβ-to-Tau inflection point. Given the scope and focus of *Nature Medicine*, we centered the main text on the cellular programs most directly linked to disease progression and resilience. We fully agree that additional analyses of other cell types will be valuable, and we expect that these will form the basis of dedicated follow-up studies. Rapid publication of the present manuscript will ensure that the field can access and build upon these resources immediately.

18. More representative IHC data generated with the spatial platforms would be helpful to understand and interpret the main findings from the study.
We agree that representative IHC data aligned with the spatial transcriptomic platforms are important for interpretation. In the revised manuscript, we have expanded these data and integrated them more directly with the spatial analyses. Specifically:

Figure 1e now includes representative Visium regions spanning low to high Aβ and pTau burden, illustrating how pathology quantification aligns with the spatial grid.
Figure 4a has been updated to show a full Xenium field with segmented cells, DAPI, Aβ, pTau, and selected transcripts, providing a direct visual link between pathology and spatial gene expression.
Figures 4g and 5j now include additional immunofluorescence staining for SPP1 and MHC-II in both the octogenarian and centenarian cohorts, demonstrating microglial expression of these markers at neuritic plaques.
Together, these additions provide clearer visual context for the spatial transcriptomic findings and directly support the pathology-associated microglial programs described in the manuscript.

19. Modules are different colors based on the analysis. So a cyan module in one figure is the brown module in another (as an example). While this is a constraint of the packages, the authors could help the reader by providing additional labels for the pathways in the figures that provide more context.
We agree that reliance on the color-based labeling of WGCNA modules can be confusing especially since module colors are assigned independently in each analysis and therefore do not correspond across cohorts or figures. While renaming modules is challenging because each captures multiple partially overlapping biological processes, we have taken several steps to improve readability and contextual interpretation.
First, in the main text, we now refer to modules using both the color label and a brief functional descriptor. For example, in the Results section: “Neuroinflammation and resilience mechanisms in centenarians” we describe:
Two modules differed markedly between cohorts. OCT-Tan, upregulated along the A-axis in OCT, was downregulated in CEN, and enriched for wound healing (GO:0042060, p-adj=7.8x10^{-8}) and angiogenesis (GO:0045765, p-adj=8x10^{-7}, Table S4). Conversely, OCT-Midnight Blue, was strongly enriched in CEN and associated with neuropeptide signaling (GO:0007218, p-adj=3x10^{-6}) and interneuron migration (GO:1904936, p-adj=5x10^{-6}).
Second, throughout the manuscript we focus analyses on the OCT-Green-Yellow module, which represents the key immune transition explored across both cohorts. For the centenarian cohort, we highlight the strong correspondence of centenarian modules to OCT modules (Fig. S7) and again anchor the interpretation around OCT-Green-Yellow to maintain consistency.
Together, these changes ensure that modules are identifiable by both color and biological context while avoiding oversimplified renaming.

20. Figure 1A: There is no description of what is being stained in the main figure or the figure legend. Please add this information to both.
We thank the reviewer for noting this omission. We have now updated both Figures and legends. Specifically figure 1A has been drastically changed and we now describe the markers in Fig 1c represented by each colour channel (Aβ: 4G8, pTau: AT8, nuclei: DAPI), and this convention is applied consistently throughout the manuscript. These additions ensure that Figure 1c can be interpreted independently and aligns with the staining descriptions provided in subsequent figures (notably Figures 1e, 4a, 4g, and 5i).

21. Figure 1B labels should include n's for the first cohort.
We have added cohort sizes to this figure (which has been moved to Figure 1a).

22. Please spell out acronyms (e.g., MMSE) prior to use to improve ease of reading, especially for audiences slightly removed from clinical side of the field.
We thank the reviewer for this helpful suggestion. We have now spelled out all clinical abbreviations, including *Mini-Mental State Examination (MMSE)* and *Amyloid–Braak–CERAD (ABC) score*, upon their first mentioning in the main text. We have also added a glossary to Table S1, listing the clinical parameters and their corresponding abbreviations for ease of reference.

23. Fig 1C: There is no label for what is being stained in the main figure. The figure legend describes abeta and pTau, but there is no mention of what color each was in IF image. Please add this information to the main figure and the figure legend. Additionally, there isn't a clear definition in the figure or the figure legend what the yellow circles are. Based on the context in the results section, these are TDs, but to improve ease of reading, please add this to the figure legend.
We thank the reviewer for pointing out these omissions. We have now revised all figures and clarified all colours codes and annotations. The previous Figure 1c has been removed during figure reorganization, but the relevant panel reappears in the updated Figure 1b. In this revised figure, we explicitly indicate the colour channels for Aβ (4G8), pTau (AT8), and nuclei (DAPI) both in the panel and in the legend. Additionally, the circles originally shown are indeed the Visium spot boundaries. These are now marked in panel 1e and clearly defined in the legend as: "White circles mark the Visium spot capture areas (55 μm diameter."

24. Fig 1H: Please label what each color is in the main figure and not just the figure legend to improve ease of reading. This panel should also be moved up in the figure to between what is currently 1C and 1D. Finally, a yellow dashed circle is mentioned in the figure legend but not included in the figure. Please add this.
We thank the reviewer for these helpful suggestions and apologize for the confusion created in the previous version of the manuscript. In the revised figures, we have reorganized the layout of Figure 1 to provide a clearer and more logical flow of the data, in line with this and other reviewer comments. We have added explicit colour labels directly onto all relevant panels to improve readability without requiring reference to the legend.

25. Fig S1C and S1D: Please add figure legend with what each color stands for. Otherwise, make the figure legend in S1B larger so it's clearer that it also applies to Fig S1C and S1D
We thank the reviewer for this helpful suggestion. Panels S1c and the first two panels of S1d have been moved to the main Figure 1 (now Fig. 1c and 1c respectively), and the remaining panels are now presented as Fig. S1b. The colors in these figures correspond to individual

samples. We've added a legend to the new Fig. 1b (previously S1D) and explicitly state the meaning of the colors in all figure legends.

26. "The separation between OCT+DEM and OCT-DEM was primarily driven by an increased number of pTau-positive TDs in the demented group". Use of the phrase "the demented group" may be considered offensive. It's advised to change the phrasing/terminology used to describe this group of individuals for example "group with dementia".
We thank the reviewer for pointing this out and apologize for the earlier phrasing. We have revised the sentence to use person-first language and to avoid terminology that could be perceived as insensitive. The corrected sentence now reads: *"indicating that tau pathology, not amyloid load, best distinguishes individuals living with dementia from those who remain cognitively intact."* We have also checked the rest of the text.

27. It's quite difficult to follow along with the figures as the panels are not all referenced in the text in the order by which they appear in the figures. Please reorganize the figures to match the order in which they are referenced in the text.
We thank the reviewer for this helpful observation. We have carefully reviewed all figures to ensure that the order of panels now matches the sequence in which they are referenced in the text. In cases where reordering panels was not practical due to layout constraints, we revised the figure legends to guide the reader through the sequence described in the text. These changes substantially improve the readability and coherence between the Results and figures.

28. Fig 1D-G are not referenced in the text. Please add them.
We thank the reviewer for drawing attention to this omission. In the revised manuscript all figure panels are now referenced in the text, or have been removed where appropriate. The previous Fig. 1D-G have been integrated into the new Fig. 1, and panels are now explicitly cited in the Results. As part of the broader reorganization of figures for clarity, the previous Fig.1g has been removed.

29. "Aβ and pTau pathology concentrated in cortical layers L3–5, affecting 62% of OCT+DEM and 41% of OCT-DEM TDs in these regions (Fig. 2)." It is unclear where this information is presented in Figure 2. Please be more specific about its location.
We thank the reviewer for catching this error. The figure reference was indeed incorrect. In the reorganized text and figures we now make the reference correctly: "Within the gray matter, layers 3–5 showed the highest pTau accumulation and strongest Aβ overlap across individuals, identifying them as the most informative region to study the Aβ–Tau interface (Fig. 1c, S1b).

30. Fig 2B-E: Please label what the color scale is denoting in these panels.
We thank the reviewer for this helpful suggestion. We have now added explicit labels to all colour scales indicating precisely what each scale represents (for example, z-normalized cell-type abundance, percentage pathology overlap, or $\log_2$ enrichment scores). We also

reviewed all other figures to ensure that every heatmap and hexbin plot includes a fully labeled colour scale.

31. “Cluster 2 represents low-pathology TDs”: What is considered “low pathology”? Please define this.
We thank the reviewer for highlighting this ambiguity. In the previous version, the term “low pathology” was imprecise. We have now removed this phrasing and clarified the biological meaning of TD2 in the revised manuscript:
“TD2 corresponds to a transitional environment where the earliest cellular responses to pathology appear; it is evenly represented across the cohort (Fig. S4) and enriched in the Exc_4 excitatory neuronal subtype and Oligo_1 oligodendrocytes subtype (Fig. 2e & S5)”
This revised wording accurately reflects that TD2 corresponds to an early or intermediate transcriptional response to pathology rather than a pathology-free state.

32. Fig 5E: There are inconsistencies in how cell clusters are referred to in the text vs in the figure (e.g., “Mic_2” vs “Micro_2”). Please make these consistent to ensure ease of reading.
We thank the reviewer for drawing attention to this inconsistency. We have reviewed the full manuscript, all figure legends and the corresponding panels to ensure uniform nomenclature. All microglial clusters are now consistently labeled using the“Mic_X” format. This correction has been applied throughout the manuscript for clarity and ease of reading.

33. SEA-AD atlas and ROSMAP datasets are referenced numerous times in this study, however little context is provided on what they include. Please add context to aid readers unfamiliar with these datasets in understanding what they are and why the similarities you’re observing are important.
We thank the reviewer for this helpful suggestion. To aid readers who may be unfamiliar with these foundational datasets, we have added brief contextual descriptions to the Introduction:
“The Seattle Alzheimer’s Disease (SEA-AD) atlas[34] is a large, multi-donor single-nucleus RNA-sequencing resource generated from human frontal and temporal cortex, covering the full spectrum of Alzheimer’s pathology and incorporating harmonised cell-type annotations across hundreds of thousands of nuclei. The ROSMAP cohort[20] combines longitudinal clinical data from older adults with post-mortem molecular profiling, including extensive single-nucleus RNA-sequencing and detailed neuropathological assessments. These datasets have been central to defining disease-associated cell states in AD, but neither includes spatial transcriptomic information, which is essential because AD brains contain a mosaic of local pathological states within the same tissue.”

34. Please provide more context on what mouse PIGs are in the results section. Some readers may be unfamiliar.
We thank the reviewer for this helpful comment. To assist readers who may be unfamiliar with the mouse plaque-induced genes (PIGs), we have added the following clarification to the *Results* section:
TREM2-related signaling genes (Table S3) and showed strong overlap with the Plaque Induced Genes identified in mouse models (mouse-PIGs; log OR: 4.06, $p = 2.2 \cdot 10^{-16}$)[23], a micro- and astroglial program induced by proximity to Aβ plaques, highlighting a conserved innate immune response to Aβ across species.
This addition provides clear context for readers without assuming prior knowledge and underscores the relevance of the cross-species comparison..

35. "…enriched in such genes including SPI1, APOC1, HLA-DRB1, MS4A6A, MS4A4A, HLA-DRB5, TREM2 and APOC2 (Fig. S5h)." "… and (e) is predominantly expressed in microglia". Prior to these statements, there is no explanation of why these genes are important or why OCT-Green-Yellow cluster being predominantly expressed in microglia is relevant to the transition between response to abeta pathology à tau pathology. Please provide more context on why microglia are important in the context of AD.

We thank the reviewer for this valuable comment. We agree that additional context on the central role of microglia in Alzheimer's disease strengthens the rationale for our findings. We have therefore added following clarifications:

Among these, the OCT-Green-Yellow module (172 genes) emerged as the dominant pathology-responsive program, showing the strongest and most consistent upregulation with increasing Aβ- and pTau burden (Fig. 3a). Genome-wide association studies (GWAS)[24] and mechanistic analyses[25] have established microglia and immune pathways as major genetic determinants of AD. The OCT-Green-Yellow module was highly enriched for microglia markers

And

Integration with AD GWAS summary statistics[24], aggregated using MAGMA[51], revealed that OCT-Green-Yellow was the only module significantly enriched for AD risk genes (Fisher's exact test, p-adj = $6{\cdot}10^{-7}$), including *SPI1, APOC1, HLA-DRB1, MS4A6A, MS4A4A, HLA-DRB5, TREM2* and *APOC2* (Fig. S7h)

This concise additions provide context for readers unfamiliar with the central role of microglia in AD pathogenesis and clarifies why the microglial enrichment of the OCT-Green-Yellow module is biologically relevant to the Aβ-to-tau transition.

36. "Genes enriched in Mic_2 include SPP1, CCL3, CH25H, and RGS." Gene names should be italicized.

We thank the reviewer for the observation. While we agree that gene symbols are commonly italicized in some journals, *Nature Medicine*'s formatting guidelines specify that gene symbols should **not** be italicized. We have therefore retained the formatting consistent with the journal's style guide (see *Nature Portfolio Submission Guidelines: Gene Nomenclature*).

37. It would be helpful to add a panel like Fig 3I to Figure 3 to show the spatial distribution of the TD clusters (bottom UMAP in Fig 2A). This would make it easier for readers to reference when assessing the data presented in Fig 3D-F.

We thank the reviewer for this thoughtful suggestion. We agree that the visual proximity of several UMAPs in the manuscript may have contributed to confusion about which analyses derive from spatial transcriptomics and which derive from single-nucleus RNA sequencing. Fig. 3h (previously Fig. 3i) represents the *single-nucleus RNA-seq microglia UMAP*, where clusters Mic_0–Mic_5 correspond to transcriptionally defined microglial states. Because these clusters are derived from snRNA-seq rather than Visium data, the Tissue Domain (TD) clusters shown in Fig. 2a cannot be directly projected onto this UMAP.

Nevertheless, to improve clarity and provide an immediate visual reference for readers, we have taken the following steps:

(i) added explicit plot titles to the new Fig. 3h (previously 3i) identifying it clearly as an "*sn-RNAseq microglia" UMAP*;

(ii) copied the TD UMAP hexbin plot from Fig. 2a into Fig 3 so that readers can directly compare the TD structure while interpreting panels 3d–f

(iii) standardized terminology for Tissue Domains throughout the figure and legend to avoid confusion between TDs and microglial states.

38. It would be helpful to add a panel in Fig 4 with a representative image of what would be mapped on the A-axis vs T-axis.
We fully agree with the reviewer that such a visual reference improves clarity. To address this, we created a new panel (now included as *Figure 1e*) showing representative Visium spots with increasing levels of Aβ and pTau pathology, illustrating how the A-axis (Aβ-associated) and T-axis (Tau-associated) are defined. This addition provides readers with an intuitive visual guide for interpreting subsequent analyses.

39. "Cell type distributions along these axes were analyzed using a rolling window approach (Fig. 4c-d)." Please provide more context on what the rolling window approach is in the results section where it is introduced.
We thank the reviewer for this helpful suggestion. We have expanded the description in the Results to clarify how the rolling-window analysis was performed. The revised text now reads:
"Cell-type distributions along these axes were analysed by ordering all Xenium pseudo-cells according to their distance from the nearest Aβ or pTau signal and then calculating the local proportion of each cell type in a rolling window of 5,000 cells, which smooths local variability while preserving spatial trends (Fig. 4c–d)."

40. Please label the color scales in Figure 5F, G, and I.
We thank the reviewer for catching this omission. All color scales in Figure 5f, 5g, and 5i have now been properly labeled to indicate what each represents (for example, percentage pathology overlap or normalized module scores). This correction has also been applied consistently across related figures for clarity.

Reviewer #2 (Remarks to the Author):

This manuscript examines Alzheimer's disease (AD) progression and cognitive resilience through spatial transcriptomics and single-nucleus RNA sequencing on brain tissue from octogenarians (with and without dementia) and cognitively intact centenarians with amyloid-β (Aβ) accumulation. Six cellular microenvironments are identified, reflecting AD progression with a transition from Aβ-driven to tau-driven pathology, marked by a shift in microglial states from early inflammatory (early plaque-induced genes, PIGs) to late antigen-presenting (late-PIGs) phenotypes. Resilient individuals exhibit distinct mechanisms: octogenarians without dementia do not induce late-PIGs, while centenarians uncouple late-PIG activation from tau accumulation. Microglial transitions are proposed as potential therapeutic targets. The technical foundation, particularly the use of spatial transcriptomics, is impressive, and the comparison of octogenarian (OCT) and centenarian (CEN) cohorts is ambitious.
We thank the reviewer for the thoughtful and encouraging assessment of our study. We appreciate the recognition of the technical strengths, in particular the integration of spatial transcriptomics with single-nucleus RNA sequencing and the value of analysing two rare and complementary human cohorts. We have revised the manuscript extensively in response to

the reviewer's comments. These revisions clarify the analytical framework, strengthen the rationale for comparing octogenarians and centenarians, and more clearly highlight the novel insights that emerge from these combined datasets.

Yet, the narrative can feel inconsistent, visuals sometimes lack interpretive clarity, and the CEN cohort's potential, particularly regarding co-pathologies and dementia trajectories, is underexplored. Overall, despite containing exciting methods and cohorts, the manuscript's results are underwhelming, with limited novel insights.
While we appreciate the reviewer's perspective, we respectfully disagree that the study provides "limited novel insights". We have revised the manuscript to improve narrative flow, clarify figure interpretation, and strengthen the integration between the OCT and CEN cohorts. We also highlight more clearly the conceptual and methodological advances enabled by this dataset.
First, we introduce a continuous spatial–pathological framework (A and T axes) that allows reconstruction of cellular transitions directly within intact human cortex, moving beyond categorical plaque annotations.
Second, by integrating spatial and single-nucleus transcriptomics across two deeply phenotyped human cohorts, we define a high-resolution sequence of glial and neuronal state changes spanning the Aβ-to-Tau transition.
Third, we identify a spatial inflection point characterized by a switch from complement-rich microglial activation to antigen-presenting late-PIG states, a feature shared across both cohorts and conserved when mapped onto mouse plaque-induced programs.
Fourth, we show that late-PIG activation can occur independently of Tau accumulation in centenarians, revealing an altered microglial trajectory that may underpin cognitive resilience.
Fifth, we use machine-learning–based cross-cohort mapping to anchor centenarian tissue domains onto octogenarian pathological niches, enabling direct comparison of resilience and vulnerability states. Together, these findings provide a coherent mechanistic framework for how local pathology shapes microglial states in the human brain and help explain why some individuals maintain cognitive function despite substantial Aβ burden. We hope these clarifications help convey the novelty and importance of the findings.

Regarding the CEN cohort, we fully acknowledge its complexity and the challenges inherent to advanced aging. Given the limited sample size and low pTau burden, we focused our analysis on Aβ-associated pathology to enable robust comparison with the OCT cohort. Despite these constraints, the CEN data reveal a notable finding: activation of late-PIG programs in the absence of Tau pathology, suggesting a decoupling of inflammatory activation from neurodegeneration, an important feature of cognitive resilience.

We agree that this dataset holds substantial additional potential. However, it represents the outcome of five years of experimental and analytical work, and our goal in the current manuscript is to establish a clear, interpretable foundation. Together with Henne Holstege, we are now expanding the CEN cohort to include additional neuropathological, clinical, and

molecular parameters. This ongoing work will allow deeper exploration of resilience trajectories in subsequent publications, building upon the framework established here.

Major points of critique

- The term “tissue domains” (TDs) is ambiguous, potentially referring to Visium spots, clusters, or another entity, making it difficult to follow without clarification on whether Visium was performed on multiple 10µm sections of 8mm punches.

This concern was also noted by Reviewer 1, and we recognize that our earlier terminology and descriptions were insufficiently clear and therefore caused confusion. We apologize for this and have now made substantial revisions throughout the manuscript to resolve the ambiguity.

First, we now explicitly distinguish between *Visium spots and Tissue domains (TDs). Visium spots* refer to the individual 55 µm capture areas generated on each 10 µm cryosection. *Tissue domains (TDs)*, in contrast, are clusters of Visium spots that share a similar transcriptomic or inferred cellular composition. This revised terminology aligns with common usage in the spatial transcriptomic field [PMID: 27365449, 37758947, 32637622]. For consistency, we have also removed the term “cellular niche,” which previously contributed to ambiguity.

Second, we clarified that Visium was performed on single 10 µm sections of each 8 mm tissue punch, and that TDs arise from clustering of Visium spots within the same section, not from combining multiple sections.

Third, we have revised section titles throughout the manuscript to better reflect the analytical workflow.

Fourth, we expanded the methodological description to explain precisely how TDs were derived. For each Visium spot, we inferred cell-type composition using Cell2Location [PMID: 35027729], corrected for layer effects using Harmony and then performed Leiden clustering to the inferred cellular abundance matrix. This yielded six TDs in the octogenarian cohort and seven in the centenarian cohort, each with a characteristic cellular signature (Figs. 2a-d and 5a). Pathological relevance was inferred a posteriori by correlating TD membership with Aβ and pTau immunofluorescence signals (Fig. 2c) and with clinical groups (OCT_HC, OCT-DEM, OCT+DEM; Fig. 2b).

Finally, to clarify the pathological framework, we now introduce at the beginning of the *Results* section (see also new figure 1e) that Aβ and Tau pathology were modeled as continuous variables (*Aβ-axis* and *Tau-axis)* derived from quantitative immunofluorescence overlap with Visium spots. This continuous model replaces the earlier categorical plaque classifications and makes explicit that spatial transcriptomic information defines TDs, while immunostaining provides contextual pathological annotation only.

- Claims about CEN co-pathologies seem overstated without robust testing or controls, which makes it challenging to fully appreciate resilience arguments, especially when compared to a controlled OCT cohort focused solely on Aβ and tau pathologies.

We thank the reviewer for raising this important point. We agree that the CEN cohort presents substantial biological complexity due to the presence of multiple age-related co-pathologies. Our intention was not to attribute specific mechanistic effects to these co-pathologies, but rather to examine how Aβ-associated transcriptional programs manifest within this advanced-aging context. To avoid overstating our conclusions, we now explicitly clarify that the CEN analyses focus solely on Aβ-linked cellular states and do not seek to model the influence of individual co-pathologies.
We also recognize that the OCT and CEN cohorts differ in both clinical composition and co-pathology burden, and that this limits direct comparison of all disease features. We explicitly enumerate the co-pathologies in the CEN and mention throughout the manuscript that we limit our analysis to the Aβ component in the CEN cohort. We write in the Discussion:
"The large number of spatially resolved Visium spots spanning a broad continuum of pathology, provides high confidence in the observed cellular transitions. The inflection points we describe are spatial–pathological rather than longitudinal, yet their coherence across tens of thousands of tissue domains indicates that they represent robust biological checkpoints. Although cohort size limits individual-level analyses such as APOE genotype effects, and despite the higher co-pathology burden in CEN brains, the reproducibility of microglial transition across both cohorts suggests a shared underlying trajectory of AD progression."
We have revised the manuscript to avoid any possible overstatement regarding co-pathologies in centenarians and to frame the CEN analyses clearly as an examination of Aβ-driven immune responses within the broader biological context of extreme aging.

- The CEN cohort's unique characteristics, such as resilience mechanisms and co-pathologies, feel underexplored, with the assignment of CEN to OCT clusters appearing arbitrary and no clear investigation into dementia trajectories or cell-type-specific signatures.
We thank the reviewer for this thoughtful comment. The assignment of CEN tissue domains to OCT-derived clusters was not arbitrary. It was performed using a supervised multi-layer perceptron classifier trained on OCT-TDs and achieving 93% accuracy in internal cross-validation. This approach ensures that CEN-TDs are interpreted within the same spatial–pathological framework as the OCT data and allows a direct and reproducible comparison across cohorts..
We fully agree that the CEN cohort provides a unique window into resilience in advanced aging. However, the modest sample size and the diversity of co-pathologies present in centenarians limit the statistical power for cell-type specific or trajectory-level analyses beyond Aβ-associated transcriptional changes. For this reason, we focused the primary analysis on Aβ-linked microglial states where both pathology burden and spatial contrast were sufficient for robust modelling.
Within this focused framework, the CEN cohort nevertheless reveals a key and non-trivial insight. Late-PIG programs are activated in CEN regions with Aβ but minimal Tau, indicating that microglial activation and Tau-linked neurodegeneration can be uncoupled. This decoupling supports the idea that microglial state transitions sit upstream of Tau pathology and that resilience can emerge by interrupting the progression from early to late immune activation.
We acknowledge that the full potential of the CEN cohort, especially for defining dementia trajectories and cell-type specific resilience signatures, is not yet realized in the present study. We are actively expanding the cohort with additional neuropathological, molecular,

and clinical data in collaboration with Henne Holstege. These ongoing efforts will allow a deeper dissection of resilience mechanisms in subsequent work, building on the spatial and transcriptional framework established here.

- Restricting weighted gene co-expression network analysis (WGCNA) to gray matter and focusing on microglia lacks clear rationale, especially given oligodendrocyte associations with tau pathology (e.g., cluster 4); exploring other cell types and justifying white matter exclusion would strengthen the analysis.

We thank the reviewer for this constructive comment and agree that the rationale for focusing the WGCNA to gray matter and highlighting microglia modules should be made more explicit. Our sampling strategy was designed to capture the cortical ribbon across layers 1-6 of the cortex. As a consequence, the amount of white matter (WM) included in each punch varied considerably across samples. Because spatial transcriptomic RNA capture is strongly influenced by myelin content and local RNA density, heterogeneous WM inclusion introduces technical variability that is difficult to normalize across donors. For this reason, we restricted the WGCNA to Visium spots mapped to grey matter, ensuring consistency and comparability across individuals.

Within the grey matter dataset, the OCT-Green-Yellow module emerged as the most robust pathology -associated network. It showed the strongest association with both Aβ and pTau burden and the highest enrichment for AD GWAS loci, providing a clear rationale for emphasizing microglial pathways in the main text.

Importantly, WGCNA also identified several additional modules corresponding to other cell types, including oligodendrocytes. These are fully described in Supplemental Data A and B. We agree with the reviewer that the oligodendrocyte signatures, particularly in TD4, suggest a possible role in Tau-associated pathology. This is an important observation and represents a promising direction for future mechanistic work, but was outside the scope of the present study.

Finally, we have clarified in the revised manuscript that we anticipate additional biological insights to be gleaned from these datasets. All raw and processed data will be made available upon publication, enabling deeper exploration of non-microglial pathways by the community.

- The significance of gene modules (e.g., the OCT-Green-Yellow module) feels overstated, as other modules (e.g., black) show similar significance; the method for determining module-pathology specificity (e.g., correlation, group comparison, multivariate modeling) and potential confounding by spatial gradients or layering effects need clearer explanation.

We thank the reviewer for this important comment. We agree that several modules, including OCT-Black, also show significant associations with pathology. Our intention was not to overstate the dominance of the OCT-Green-Yellow module, but to highlight it as the most integrative and consistently pathology-linked module across multiple independent criteria.

1. It shows the strongest and most consistent correlation with both Aβ and pTau burden across layers 3–5 (Fig. 3a), using gene–pathology correlation scores summarised at the module level by GSEA.

2. It is the most microglia-enriched module (Suppl. Fig. S7a), aligning with well-established microglial involvement in AD progression.
3. It shows the highest enrichment for AD GWAS loci and significant overlap with mouse plaque-induced genes (PIGs), supporting biological relevance across species.
4. It is selectively enriched in the highly pathological TD3 and TD4, aligning module activity with spatial progression of disease.

We employed several independent methods to determine module specificity

1. We assessed whether covariates should be included in differential expression analyses, but several variables (for example mitochondrial read fraction) were themselves strongly correlated with local pathology and likely reflect genuine biological responses to Aβ and pTau. Including such covariates would risk regressing out meaningful biology, so no covariates were applied.
2. Enrichment analyses were performed using GSEA on ranked differential expression lists for each cell type versus all others from our snRNA-seq reference, without additional covariate adjustment.
3. Overlap with AD GWAS loci was evaluated using Fisher exact tests (Bonferroni-corrected), comparing our WGCNA modules to MAGMA-derived genes from Kunkle et al. [PMID: 31417202].
4. Tissue Domains (TDs) were derived from Visium data restricted to cortical layers 3–5, where Aβ and pTau pathology are most abundant, after Harmony correction for laminar effects. Module scores were computed per Visium spot and compared across TDs using one-sided, Bonferroni-corrected Mann–Whitney U tests.

These points are now explicitly described in the revised Methods to ensure full transparency of the analytical framework.

- Despite its technical strengths, the study struggles to deliver novel insights, missing opportunities to fully leverage spatial transcriptomics or the CEN cohort to uncover new aspects of resilience or AD progression.

We refer to our response to the referees comments at the beginning of their critique for a full discussion of the conceptual advances, but summarize here the specific novel insights that become accessible only through the spatially resolved framework used in this study.
Our work introduces a continuous spatial–pathological model (A- and T-axes) that maps microglial state transitions along the no-pathology → Aβ → Aβ+pTau gradient within intact human cortex. This allows us to identify early-PIG and late-PIG programs as spatially ordered microglial states and to localize a discrete inflection point marking the shift from Aβ-driven innate activation to Tau-associated antigen presentation. This spatial reconstruction, made possible by tens of thousands of Visium spots per cohort, is not accessible through classical histopathology or snRNA-seq alone.
The integration of spatial transcriptomics with snRNA-seq across two rare, deeply phenotyped human cohorts provides an additional conceptual advance: in centenarians, late-PIG programs are activated in the absence of Tau pathology, revealing an altered inflammatory trajectory that may represent a molecular signature of cognitive resilience. To

our knowledge, such a decoupling of antigen-presenting microglial states from Tau accumulation has not been previously observed in human tissue.
These insights are now highlighted more clearly in the revised Discussion: that spatially defined microglial transitions represent key checkpoints in AD progression, and that resilience may arise either by preventing the transition to the late-PIG state or by uncoupling late-PIG activation from Tau deposition.
These insights are now highlighted more clearly in the revised Discussion: that spatially defined microglial transitions represent key checkpoints in AD progression, and that resilience may arise either by preventing the transition to the late-PIG state or by uncoupling late-PIG activation from Tau deposition.

Minor critiques

- Several figure panels feel redundant (e.g., Fig. 2A/2H) or unnecessary (e.g., Fig. 1D UMAP); merging Figures 1 and 2 could streamline presentation, as Figure 1 focuses on experimental setup while Figure 2 presents biological data.
We thank the reviewer for this helpful suggestion. In response to this and related comments from the other reviewers, we have substantially reorganised the figure set to improve narrative flow and remove redundancies. Several panels identified as duplicative (such as the previous Fig. 2A/2H) have been removed or consolidated, and the UMAP panel in the original Fig. 1D has been omitted. We also critically re-evaluated the separation between Figures 1 and 2; although full merging was not feasible due to space and layout constraints, we simplified both figures and clarified their respective roles, with Figure 1 now focused on the spatial framework and pathology quantification, and Figure 2 presenting the resulting tissue domains and biological interpretations. We believe these changes considerably improve clarity, reduce visual redundancy, and strengthen the overall logic of the manuscript.

- Missing or unclear labels (e.g., Fig. 1H lacks protein/color labels; Fig. 3B has swapped tau labels) reduce interpretability; adopting colorblind-friendly palettes (e.g., magenta/green instead of red/green) would improve accessibility.
We thank the reviewer for the careful reading and for highlighting specific issues that reduced interpretability. We have now systematically reviewed all figures and corrected missing or swapped labels (for example, the protein/color annotations in the previous Fig. 1h and the tau labels in Fig. 3b). Clear color keys are now embedded directly within each panel. In addition, to improve accessibility, we have replaced red/green combinations with colorblind-friendly palettes (for example, magenta/green) across all figures and legends. Together with the broader figure refinements prompted by reviewer feedback, these changes have substantially improved clarity and interpretability.

- Including spatial plots (e.g., q05 abundance, deconvoluted cell type distributions) and hierarchical clustering for heatmaps could enhance clarity; a UMAP labeled by deconvoluted cell types might aid interpretation.

We thank the reviewer for this constructive suggestion. In response, we have added a new Supplemental Figure 5 that includes TD-UMAPs labeled by Cell2Location deconvoluted cell types and subtypes. These panels provide a clearer visual link between spatial clustering and inferred cellular composition.

- Figure 2's color schemes are confusing, with clusters and heatmaps using similar colors without clear correspondence, making it hard to follow.
We thank the reviewer for bringing this to our attention. In response to this comment and similar feedback from other reviewers, we have redesigned Figure 2 to improve visual clarity and eliminate any unintended correspondence between cluster colors and heatmap scales. Cluster identities now use a dedicated, discrete color palette, while heatmaps employ continuous, non-overlapping color gradients. The relationship between panels is now clearly indicated in the figure legend. We believe the revised Figure 2 is substantially clearer and easier to follow.

- The CEN cohort's biphasic distribution (Fig. S1C) may reflect sex differences or other variables (e.g., TDP-43), but these are not explored, leaving potential insights unaddressed
We thank the reviewer for this thoughtful observation. During sample selection we attempted to randomize for all relevant parameters, such as sex, APOE, co-morbidities, sample quality, as much as availability allowed. We do agree that the biphasic distribution seen in the CEN cohort (Fig. S1c) may indeed reflect underlying inter-individual factors such as sex, APOE genotype, or additional age-related co-pathologies including TDP-43. However, the current sample size does not permit robust statistical testing of these variables. For this reason, our analysis was designed to focus on pathology-associated transcriptional trajectories derived from the large number of spatially resolved tissue domains, rather than on per-individual stratifications with limited power.
We fully agree that such variables may shape resilience in advanced aging, and we now explicitly acknowledge this in the Discussion. The revised text reads:
The large number of spatially resolved Visium spots spanning a broad continuum of pathology, provides high confidence in the observed cellular transitions. The inflection points we describe are spatial–pathological rather than longitudinal, yet their coherence across tens of thousands of tissue domains indicates that they represent robust biological checkpoints. Although cohort size limits individual-level analyses such as APOE genotype effects, and despite the higher co-pathology burden in CEN brains, the reproducibility of microglial transition across both cohorts suggests a shared underlying trajectory of AD progression
This limitation and its implications are also discussed in our response to Reviewer 1, comment 12.

- Running WGCNA separately on CEN and assigning CEN to OCT clusters feels arbitrary, potentially overlooking unique CEN clusters.
We thank the reviewer for this thoughtful comment. Our decision to anchor the analysis on OCT-derived WGCNA modules reflects the deliberate design of the OCT cohort, which spans defined Aβ and pTau stages and therefore provides a structured framework for identifying transcriptional modules specifically linked to AD progression. In contrast, the CEN cohort represents a heterogeneous population with multiple age-related co-pathologies and

variable cognitive trajectories, making an unsupervised WGCNA less interpretable in the context of AD-specific biology.
To enable rigorous cross-cohort comparison, we mapped CEN tissue domains onto the OCT reference using a supervised multi-layer perceptron (MLP) classifier trained on the OCT TDs. This classifier achieved 93 percent accuracy in cross-validation, ensuring that CEN transcriptional states are placed within a biologically validated coordinate system rather than assigned to potentially unstable de novo clusters. This strategy allows us to directly test whether AD-associated trajectories defined in the OCT brains are preserved, altered, or decoupled in centenarians.
We agree that centenarians may also deploy transcriptional programs unique to resilience and extreme aging. To acknowledge this explicitly, we have added the following sentence to the Discussion:
OCT-derived WGCNA modules provided a shared analytical backbone for cross-cohort comparison, yet centenarians may also deploy resilience-specific programs not fully captured by OCT-derived modules. Larger cohorts will be required to define these pathways systematically. In this context, the OCT-Tan (wound healing and angiogenesis) and OCT-Midnight Blue (neuropeptide signaling and interneuron migration) modules may offer initial clues.

- Stratifying TDs based on Aβ/tau status without considering neighboring TDs (e.g., A+T- TDs surrounded by T+ TDs) may introduce bias; accounting for spatial context could strengthen the analysis.
We thank the reviewer for this thoughtful observation. We agree that local spatial context, including the pathological status of neighboring tissue domains, may influence cellular states and could in principle refine TD classification. Our current framework does not explicitly model spatial adjacency, but instead relies on continuous, per-spot quantification of Aβ and Tau burden across many thousands of Visium spots. This dense sampling provides substantial statistical power and yields highly reproducible enrichments of early- and late-PIG states across individuals, supporting the robustness of the inferred trajectories.
Spatial adjacency between tissue domains was not explicitly modelled, although future neighborhood-aware approaches may refine local interactions further. Trajectory inference tools were explored but did not increase robustness beyond the spatial–pathological ordering presented here, and cell-type specific trajectories will be addressed in follow-up work.

- The regression of cortical layers using Harmony and potential layer-specific biases in clusters need clearer explanation.
We thank the reviewer for this comment and the opportunity to clarify our rationale. Our aim in applying Harmony *[PMID: 31740819]* was to ensure that clustering reflected pathology-associated variation rather than laminar transcriptional differences, which are a dominant source of structure in the cortical transcriptome. Layer identity was therefore provided as a covariate so that Harmony would regress out systematic layer effects while preserving biological variation related to Aβ and pTau pathology. This approach allowed Visium spots from layers 3–5 to be clustered based on shared cellular composition and pathology-associated transcriptional programs rather than laminar position. We acknowledge that any layer-correction strategy has limitations, and we now state explicitly in the Methods that our intention was to mitigate layer-driven clustering rather than eliminate all laminar signals.
To improve clarity, the Result section has been revised as follows:

To deconvolve cell composition within Visium spots, we applied Cell2Location[48] confirming layer specific enrichment of excitatory neurons (Fig. S3, Supplemental data A). Harmony[49] was then used to correct for layer effects, followed by unbiased Leiden clustering which identified 6 distinct clusters of Visium spots, termed Tissue Domains (TDs: OCT-TD0 to OCT-TD5, Fig. 2a), each representing a microenvironment with a characteristic cellular composition (Fig. 2b-e). Pathological relevance was inferred *a posteriori* by correlating TD composition with the OCT cohorts (Fig. 2b) and local Aβ and pTau burden (Fig. 2c), rather than used as input for clustering.

And in the methods:

Tissue Domain clustering and visualization We performed clustering on the cell2location cell type abundance matrix. We first normalized these abundances to z-scores based on the total cell abundance per Visium spot. We then used the mid-layer (layer 3, 4 and 5) Visium spots of our 24 OCT subjects. To mitigate layer-specific variations, we apply Harmony[49] integration on the layer. This integration removes laminar transcriptional structure while preserving biological variation associated with local Aβ and pTau pathology. The same procedure was used for the 20 CEN subjects. Subsequently, Leiden clustering is applied to cluster these Visium spots, only based on cell abundance, into six distinct clusters, termed “Tissue Domains” for the OCT (named Octogenarian Tissue Domain OCT-TD0 to OCT-TD5) cohort and seven Tissue Domains for the CEN cohort (named Centenarian Tissue Domain CEN-TD0 to CEN-TD6). The clustering results are then visualized using UMAP (Uniform Manifold Approximation and Projection). To enhance visualization and address overplotting we employ Hexbin plots (using Matplotlib[86]. Hexbins with less than five Visium spots are grayed out. The hexbin plot employs three distinct coloring schemes: (1) Percentage score (using the fire color scheme) is used for categorical variables, indicating the proportion of Visium spots associated with a particular categorical variable. (2) The average score represents the aggregated mean of a continuous variable among the Visium spots within each hexagon. (3) The mean difference score (using a blue-red color scheme) reflects the difference in the aggregated, z-normalized, mean of a continuous variable, or between the z-normalized module scores of gene sets between OCT+DEM and OCT-DEM Visium spots within each hexagon.

- Fig. 1H lacks the yellow dashed circle mentioned in the legend, causing confusion.

We thank the reviewer for noting this inconsistency. Figure 1 has been substantially revised and reorganized, and all missing elements between panels and legends have been corrected. In the revised layout, the yellow dashed circle referenced in the previous legend should not have been associated with the original Fig. 1H. This has now been resolved: the yellow circle appears correctly in the new Figure 1e, where it marks the Visium spot boundaries, and the corresponding legend has been updated accordingly. The revised figure set no longer contains the inconsistency.

- Fig. 2D’s microglia enrichment in homeostatic/pathology-free clusters seems inconsistent with the microglia-tau pathology link; the significance of plaque/tau overlap in homeostatic clusters (Fig. 2F) is difficult to appreciate without further analysis.

We thank the reviewer for this careful observation. Upon re-examining the code underlying Fig. 2d, we identified an error in the plotting step that incorrectly assigned microglial enrichment to the homeostatic TDs. We sincerely apologize for this oversight. After correction, the expected pattern is now clearly recovered: microglia are enriched in the pathology-associated TD3, consistent with their known role in Aβ- and Tau-associated inflammation, while TD4 is predominantly oligodendrocyte-driven.

In light of this correction, the correspondence between microglial enrichment (Fig. 2d) and local Aβ/pTau burden (Fig. 2c) is now more coherent, and the pathological significance of TD3 is much easier to appreciate in the revised figure. We have updated both the figure and the legend and verified all related analyses for internal consistency.

- Fig. 3C's terminology (differential expression vs. gene set enrichment analysis) is inconsistent, and the comparison being performed is unclear.

We thank the reviewer for this helpful comment. We have clarified the comparisons underlying Fig. 3c and harmonized terminology across the figure, legend, Results, and Methods. Fig. 3c shows all genes from the OCT–Green-Yellow module, plotted according to two gene-level differential expression contrasts:

- The A-axis (x-axis) represents a differential expression analysis against the Aβ load with no pTau present (in the A+T− and A−T tissue domains).
- The T-axis (y-axis) represents a differential expression analysis against the pTau load in presence of Aβ (in the A+T+ and A+T− tissue domains).

By performing two pre-ranked GSEA analyses of the OCT-Green-Yellow module against both ranks we defined the early PIGS as the leading-edge genes found in the A-axis and equivalently, the late PIGs in the T-axis. The plot indicates the OCT-Green-Yellow genes in these two axes.

We have revised the Fig. 3c legend to use consistent terminology ("gene-level differential expression," "pre-ranked GSEA," "A-axis"," "T-axis") and updated the corresponding Results text for clarity.

(c) Gene-level differential expression of OCT-Green-Yellow module genes along the A- axis and the T-axis. Each point represents one gene from the OCT-Green-Yellow module positioned according to the log fold-change of the differential expression in the **A-axis** (x-axis, differential expression against Abeta in absence of pTau in the A+T− & A−T− Visium spots) and the **T-axis** contrast (y-axis, differential expression against pTau in presence of Abeta in the A+T+ & A+T− Visium spots), computed from Visium spots in layers 3–5. The colors indicate **early-PIGs** (leading-edge genes enriched along the A-axis, red), **late-PIGs** (leading-edge genes enriched along the T-axis, green), shared PIGs (genes contributing to both enrichments, purple), and remaining module genes (gray).

We have also revised the relevant Result section accordingly.

- Fig. 3G-L's focus on microglia (e.g., mic_5 aligning with late-PIGs) is difficult to fully appreciate, as oligodendrocytes are enriched in tau-associated clusters; imaging data could bolster these findings

We thank the reviewer for this valuable suggestion. Our primary objective in Fig. 3 was to dissect the microglial transitions that define the Aβ–Tau inflection point, as these represent the dominant and most reproducible pathology-associated signatures across octogenarians and centenarians. The alignment between mic_5 and the late-PIG gene set is supported by both Visium spatial mapping and Xenium single-molecule validation, strengthening the interpretation of this microglial trajectory.

We agree that oligodendrocytes also show Tau-associated transcriptional changes, particularly within TD4. These observations are included in the manuscript through GSEA of oligodendrocyte-enriched modules (for instance Fig. S5a) and further exploratory analyses provided in Supplemental Data A. However, given the space constraints of the main text and the focus of the current study on the Aβ–Tau microglial transition, we did not expand these results further. We acknowledge the reviewer's point that additional imaging-based validation

of oligodendrocyte states would strengthen the link with pTau pathology. We are currently extending these analyses as part of ongoing work and expect to present a fuller cell-type–resolved picture in a dedicated follow-up publication. The present manuscript lays the spatial and transcriptional framework necessary for these future studies.

- Fig. 4C/D regression plots may not add significant value and resemble loess plots; oligodendrocyte enrichment near plaques/tau is noted but underexplored.

We thank the reviewer for this comment. The original Fig. 4c–d indeed combined two types of trend visualizations: linear regression lines and rolling-window averages. The latter were sometimes visually reminiscent of LOESS curves, although they represent 5,000-cell rolling averages used to illustrate aggregate trends along the Aβ and Tau axes. Our goal was descriptive visualization more than formal statistical regression.

In line with the reviewer's suggestion, and to avoid over-interpretation, we have removed the linear regression lines entirely and retained only the rolling-window averages, which convey the same information more transparently and without implying an overly simplistic linear relationship.

Regarding oligodendrocytes, we agree that their enrichment near plaques and Tau pathology is noteworthy. We now refer to these patterns explicitly in the Results section for instance when we discuss TD3 ("Single-cell spatial profiling confirms early- and late-PIG expression dynamics") and cross-reference Supplemental Fig. S5 f-g and Fig.S6. A fuller exploration of oligodendrocyte responses is beyond the scope of the current manuscript but will be pursued in ongoing follow-up analyses.

- Fig. 5's assignment of CENs to OCT clusters feels confusing and lacks clear rationale; focusing on cell-type-specific signatures might be more informative.

We thank the reviewer for this constructive comment. Because we did not generate single-nucleus RNA-seq for the CEN cohort, direct inference of centenarian-specific cell states was not feasible. To enable biologically meaningful cross-cohort comparison, we therefore aligned CEN Visium data to the OCT-derived tissue domains using a two-step, fully data-driven approach:

1. Cell2Location deconvolution: CEN Visium spots were deconvolved using the OCT snRNA-seq reference to obtain cell-type compositions.
2. Supervised classification: These compositions were assigned to OCT tissue domains using a multi-layer perceptron (MLP) classifier trained on OCT TDs (93 percent cross-validated accuracy).

This strategy avoids arbitrary matching and places CEN transcriptional niches into the same spatial–pathological framework established in the OCT cohort, allowing direct comparison of Aβ- and Tau-associated trajectories.

We agree that cell-type–specific signatures are equally important. To address these independently of TD mapping, we performed WGCNA and GSEA on CEN Visium data, which show that the OCT-Green-Yellow microglial module remains strongly enriched along the CEN A-axis, whereas other modules exhibit cohort-specific behavior. These results demonstrate that the key conclusions about conserved early-PIG activation and decoupled late-PIG activation in centenarians are not dependent on the tissue-domain assignment

procedure. Together, the supervised TD mapping and independent module-based analyses provide complementary and robust insights into the inflammatory trajectories in centenarians.

- The generalizability of early/late-PIG dynamics to other brain regions (e.g., hippocampal CA1) remains unclear

We agree with the reviewer that the regional generalizability of early- and late-PIG dynamics is an important question. As noted in the Discussion, our analysis was restricted to cortical layers 3–5 of the frontal cortex, and other brain regions may show additional or distinct cellular transitions.

The analysis focused on cortical layers 3 to 5 of the frontal cortex, and additional spatial trajectories may exist in other brain regions that were not sampled here.

The present study focused on the superior frontal gyrus to ensure high-quality spatial resolution and consistent representation of the six cortical layers while minimizing variability introduced by regional differences.

Follow-up studies are ongoing to assess whether similar microglial transitions occur in other brain regions, including hippocampal CA1 and the entorhinal cortex, using both spatial transcriptomics and in situ validation approaches. These forthcoming datasets will allow us to determine the extent to which the early- and late-PIG trajectories identified here represent a conserved feature of AD progression across the human

- The claim that late-PIGs are uncoupled from tau in CENs is difficult to appreciate, as CENs have less tau pathology overall; evidence linking late-PIGs to tau prevention, rather than co-occurrence with resilience, would strengthen the argument.

We thank the reviewer for raising this important point. We agree that, given the overall lower Tau burden in centenarians (CEN), it would be inappropriate to imply that late-PIG activation directly prevents Tau pathology. Our interpretation is more cautious: in the OCT cohort, late-PIG activation emerges almost exclusively in tissue domains with both Aβ and Tau pathology, whereas in CEN, an inflammatory program with the same late-PIG transcriptional features is induced by Aβ alone, in the relative absence of Tau.

Thus, our conclusion is not that late-PIGs *block* Tau accumulation, but that late-PIG activation is not intrinsically coupled to Tau pathology, and that its downstream consequences depend on the broader cellular context. In centenarians, late-PIG expression co-occurs with preserved cognition and minimal Tau, suggesting that this state *can* arise in a non-pathogenic form. This distinction is now clarified in the revised Discussion.

We identified six Tissue Domains (TDs), with TD3 and TD4 marking the critical transition from Aβ-associated inflammation to pTau-associated degeneration. This shift coincided with a microglial change from early complement and TREM2 enriched activation states (early-PIGs) to later antigen-presenting phenotypes (late-PIGs). Two resilience strategies emerged. In octogenarians without dementia microglia mounted an early-PIG response but did not progress to the antigen-presenting late-PIG state, consistent with a restrained or self-limiting inflammatory trajectory. In centenarians, late-PIG programs were engaged despite the very low levels of Tau pathology. This indicates that late-PIG activation is not intrinsically pathogenic, but that its downstream consequences depend on the surrounding cellular and pathological context. Spatial transcriptomics and new immunofluorescence staining (Fig. 5j) both confirm that HLA-DR and SPP1 positive microglia can be found in Aβ-rich but pTau-poor regions, supporting this dissociation. In these individuals, late-PIG activation remains uncoupled from pTau accumulation and neurodegeneration, suggesting a resilience mechanism grounded not in

avoiding microglial activation, but in preventing its progression toward Tau-linked toxicity. Whether this altered trajectory reflects genetic, immune, or ageing-related factors in centenarians remains to be determined.

Statistical Concerns

- When significance is shown in figures, specifying the statistical test (e.g., Mann-Whitney U, Welch's test) would improve transparency.
- Clarifying whether p-values are raw or adjusted, with adjustments for multiple comparisons as needed, would enhance rigor.
- Specifying whether tests are one-sided or two-sided, particularly for Mann-Whitney U, is necessary for clarity.

We fully agree with the reviewer. We performed a systematic audit of all figures and legends and now explicitly state the statistical tests used for each comparison (e.g., Mann–Whitney), including whether tests were one- or two-sided and whether p-values are raw or multiple-comparison–adjusted. Where applicable, Bonferroni or FDR corrections are now specified directly in the figure legends. These updates have been applied consistently across all main and supplementary figures to ensure transparency and reproducibility. A dedicated Statistical Analysis subsection has been added to the Methods, detailing statistical procedures.

- For Cell2Location models (RegressionModel, Cell2location model), specifying the batch variable (e.g., sample ID) or providing a rationale for its absence would improve reproducibility.

We thank the reviewer for this valuable suggestion. We agree that transparency regarding batch and covariate handling in Cell2Location is essential for reproducibility. The Materials and Methods section has now been updated to clearly describe all batch variables and covariates used.

In the RegressionModel, we included sequencing lane ID as a batch covariate, the prefrontal cortex subregion (GFS) as a categorical covariate, and RNA integrity (RIN_NBB) as a continuous covariate to account for sample-level and regional variation. These covariates were selected based on preliminary QC analyses showing that they contributed independent variance without correlating with pathology.

For the Cell2Location spatial mapping model, Visium slide ID was included as a batch covariate, together with the same GFS and RIN_NBB covariates used in the RegressionModel. This ensures consistent correction across the reference and spatial models and avoids technical structure being misinterpreted as biological signal.

These details are now explicitly described in the Methods as follows:

Cell deconvolution. To spatially map brain cell types and states, as identified from the single nuclei analysis, onto the spatial Visium data, we employed the Cell2Location method[48]. In brief, we trained the snRNA-seq data using the RegressionModel with hyperparameters batch_size = 2500, train_size = 1, lr = 0.002, and max_epochs = 200. For the RegressionModel we used sequencing lane ID as a batch covariate, the prefrontal cortex subregion (GFS) as a categorical covariate, and RNA integrity (RIN_NBB) as a continuous covariate to account for regional and sample-level variation. Subsequently, we estimated the cluster mu by simulating 1000 samples from the posterior distribution, using the same batch size. The inferred reference cell state signatures obtained from this process were then utilized in the cell2location algorithm to estimate cell type abundance in each Visium

spot. For the Cell2Location deconvolution model, we utilized the hyperparameters N_cells_per_location = 8, detection_alpha = 200, max_epochs = 30000, batch_size = None, and train_size = 1. We used Visium slide ID as a batch covariate along with the same GFS and RIN_NBB covariates. The same configuration as the scRNA-seq model was used to draw samples from the posterior distribution for estimating the cell abundance score. In downstream analysis, we considered the 5% quantile of this posterior distribution (q05_cell_abundance) as a conservative estimate of cell abundance in each Visium spot.

- In Extended Data Figure 1D, justifying the use of Welch's test over a paired test for repeated measures, or clarifying that repeated measures do not apply, would strengthen the analysis.

We thank the reviewer for raising this important point. Following the restructuring of figures, the original Fig. S1D is now represented across Fig. 1c and Fig. S1b. We agree that Welch's t-test was not the most appropriate approach for these comparisons and have reanalyzed the data using a permutation-based nonparametric ANOVA to improve statistical rigor and transparency. Importantly, repeated-measures testing does not apply here because pathology values were summarized at the level of donor × cortical layer, and comparisons were made between phenotype groups, not within donors across conditions. In the revised analysis, we tested whether pathology levels differed across phenotype groups (AD+DEM, AD−DEM, AD_HC, and CEN) within each cortical layer for all five pathological markers (Aβ plaques, pTau, cored plaques, neuritic plaques, and diffuse plaques) (Fig. 1c and S1b). P-values were Bonferroni-corrected for multiple testing. All associated figures and legends have been updated accordingly, and the statistical framework has been reviewed for consistency across the manuscript.

To ensure full transparency, we added the following to the Methods:

Pathology differences across cortical layers. To examine the distribution of pathology load across cortical layers (Fig. 1c), Aβ and pTau levels were expressed as log-transformed mean percentages per cortical layer and donor. For each pathological marker, values were averaged per donor and per layer. Differences between phenotype groups (OCT+DEM, OCT−DEM and CEN) within layers were tested using a permutation-based ANOVA. Under the null hypothesis of no group effect, phenotype labels were permuted within each layer while preserving donor-level pathology distributions (5,000 permutations). For each permutation, a between-group test statistic was recomputed and compared to the observed statistic to obtain an empirical P-value. P-values were Bonferroni-corrected across cortical layers ($\alpha = 0.05$)

- Hierarchical clustering could improve heatmap clarity, and supplementary UMAPs (single-cell: sample ID; C2L clustering: layer, sample ID) would aid integration visualization.

We thank the reviewer for this helpful suggestion. In response we have added supplementary UMAPs that visualize both single-nucleus RNA-seq embeddings and the Cell2Location-inferred cell-type abundance embeddings annotated by sample ID and cortical layer. These appear in the revised manuscript as Supplementary Fig. S5 and are referenced in the Results section ("Six distinct Tissue Domains define AD progression in octogenarian brains"). They provide intuitive visual confirmation that the integration across samples and layers performed as expected and that the Tissue Domain clustering is not driven by batch or regional artefacts.

We limited hierarchical clustering to Fig. S1a, where it is informative for visualizing sample-level relationships. For the other heatmaps, we chose structured layouts (e.g., grouping by cell types in Fig. 2e), as we felt hierarchical clustering would not improve interpretability.

- Evaluating the value of hierarchical clustering of samples would help assess its contribution to the analysis.

We thank the reviewer for this constructive comment. Hierarchical clustering was used only in a descriptive, exploratory capacity and was not used for any inferential analysis or for defining Tissue Domains. Its sole purpose was to visualise how individual samples relate to one another based on their global pathology features (Aβ burden, pTau burden, ABC scores, and related neuropathology metrics). This is shown in Supplementary Fig. S1a.

The clustering revealed that centenarian (CEN) samples align most closely with OCT−DEM cases despite their substantial Aβ pathology. This pattern is consistent with preserved cognition and supports the interpretation that centenarians display a distinct resilience profile. We now state this explicitly in the Results section to ensure that the descriptive nature and limited analytical role of the hierarchical clustering are clear to the reader.

Hierarchical clustering of Visium pathology metrics and ABC staging, used only for descriptive visualization,

- Including spatial plots of q05 abundance would enhance visualization of deconvolution results.

We thank the reviewer for this helpful suggestion. In response, we have added a new Supplementary Figure S6 that visualizes the Cell2Location-derived q05 abundance estimates for all major cell types and subtypes across the sampled cortical regions. These plots provide a clearer overview of the deconvolution results and their laminar organization, and they complement the tissue domain analyses in the main figures.

- Fig. 2E's subtype enrichment quantification and whether Cell2Location used subtypes in the reference need clearer explanation.

We thank the reviewer for this helpful point. Cell2Location was run using our OCT single-nucleus RNA-seq reference, which contains all major cell types as well as their transcriptionally defined subtypes. These subtypes were therefore explicitly included in the deconvolution step.

The subtype enrichment values shown in Fig. 2e were computed as og_2(observed/expected), where the observed value corresponds to the mean Cell2Location-inferred abundance of that subtype within a given Tissue Domain, and the expected value is calculated using the product of the subtype's mean abundance across all domains (row mean) and the domain's mean total abundance across all subtypes (column mean), scaled by the overall mean, under an assumption of independence. This approach mirrors standard contingency-table enrichment logic and allows unbiased identification of subtypes disproportionately represented in each domain.

We have updated both the Figure 2 legend and the Methods section ("Statistical analysis" and "Cell2Location deconvolution") to clearly explain the inclusion of subtypes and the exact enrichment calculation.

Cell-type enrichment within TD clusters. To assess cellular enrichment within TDs (Figs. 2d and 2e), cell-type and subtype abundances were estimated using Cell2location. Expected values under independence were calculated from group means (mean abundance per TD and per cell type). Enrichment was quantified as $\log_2$(observed / expected). One-sided P-values for over-enrichment were derived from standardized residuals and corrected for multiple testing using the Bonferroni method.

- Fig. 4C/D's regression should be evaluated for added value or considered for simplification/removal.
We thank the reviewer for this helpful suggestion. We agree that linear regression lines did not add interpretive value and risked implying a model fit that was not intended. The underlying trends are better represented by the 5,000-cell rolling window averages alone. We have therefore removed all regression lines from Fig. 4c–d and simplified the panels to focus only on the cell types that show meaningful and biologically interpretable changes along the A- and T-axes.

- Reordering comparisons (e.g., A+T- vs. A-T- instead of A-T- vs. A+T-) would improve clarity.
We agree with the reviewer. All comparisons have been reordered and standardized for clarity throughout the manuscript. In the section "Dissecting early and late plaque-induced gene programs in AD," the axes are now defined consistently as follows:
This enabled two orthogonal comparisons (illustrated in Fig. 1e): the A-axis captures Aβ- associated changes without pTau (A+T- versus A-T- (n=6,885) and the T-axis captures changes on an Aβ background (A+T+ versus A+T-; n=8,463).

Reviewer #3 (Remarks to the Author):

To investigate infliction points in AD pathology, the authors utilized spatial transcriptomics and single-nucleus RNA sequencing on brain tissue from octogenarians both with and without dementia, as well as from centenarians who exhibited consistent Aβ and tau accumulation. They focused on small tissue domains near Aβ plaques or tau pathology and validated results with Xenium in situ hybridization. Unbiased clustering of the ~60 000 tissue domains (TDs) from 24 individuals revealed six distinct TD clusters associated with pathology and cognitive status. Cluster 0 reflected homeostatic states; Clusters 1–2 were transitional, 3–4 showed advanced inflammation, and 5 indicated late-stage neuronal dysregulation. These clusters outline a spatial trajectory from homeostasis to advanced AD pathology characterized by increasing inflammation and tau accumulation. Focusing on microglia within these TD, they identified an immune transition point where a switch in microglial states, from early inflammatory (early PIGs) to late antigen presenting phenotypes (late PIGs) occurred. The shift from early to late microglia across the TD seems minimal and occurred mostly form cluster 3 to 4. The TD domains from centenarians were comparable to those form the octogenarians. However, octogenarians seemed to avoid late microglial activation, while centenarians tolerated it without progressing to tau pathology.
In summary, this is an interesting approach attempting to determine cellular changes that associate with infliction point such as the conversion to tau pathology.
We thank the reviewer for their careful and constructive summary of our work and for recognizing the novelty of our integrative approach, combining spatial transcriptomics, single-nucleus RNA sequencing, and in situ validation across two rare and deeply phenotyped human cohorts. We particularly appreciate the reviewer's emphasis on the spatial trajectory of cellular states and the identification of an immune transition point in microglia, as well as the contrasting responses observed in octogenarians and centenarians. These indeed represent central strengths of the study.

In revising the manuscript, we have taken several steps to clarify and better highlight these aspects:
1. Clearer articulation of the spatial trajectory. We expanded the description of the six Tissue Domains (TDs) and explicitly linked their cellular composition to the spatial–pathological continuum, making the progression from homeostasis to Aβ-driven inflammation and ultimately Tau-associated degeneration more intuitive to follow.
2.Emphasis on the microglial inflection point. We refined the Results section on early- and late-PIGs to make the transition between TD3 and TD4 more explicit, including revised GSEA and spatial mapping panels that visually reinforce the emergence of antigen-presenting microglial states at the Aβ–Tau interface.
3. Clarification of OCT versus CEN responses. We strengthened the comparative analysis between octogenarians and centenarians, making it clearer that:
• OCT–DEM individuals mount early-PIG responses but do not transition into late-PIG activation;
• CEN individuals do activate late-PIG programs, yet remain largely free of Tau aggregation.

This contrast highlights distinct resilience strategies: restraint in octogenarians and tolerance or controlled resolution in centenarians.
4. Clarification on imaging validation. In response to the reviewer's comment about histological comparison, we now explicitly state that immunohistochemistry and Xenium are used as *qualitative* validation tools. Given that IHC can interrogate only a handful of proteins per slide, whereas spatial modules comprise hundreds of genes, we treat the imaging data as orthogonal confirmation of spatial patterns rather than a one-to-one quantitative replication.
We appreciate the reviewer's framing that this study identifies cellular changes associated with the inflection toward Tau pathology. We believe the revisions have strengthened the clarity and impact of this contribution.

Major points:

1) Validation is missing. The authors should aim at validating early and late microglia. It is essential to know whether they are able to confirm their bioinformatical analysis using orthogonal approaches. The authors could for example use immunohistochemistry with antibodies against the identified markers to validate these results. This is important as the difference of early and late PIGs shown in Figure 3d and 3e seem to be minimal.

We thank the reviewer for highlighting the importance of orthogonal validation. In response, we have substantially strengthened the experimental confirmation of early- and late-PIG microglial states.
1. Xenium in situ hybridization as orthogonal spatial validation. We would like to emphasize that the Xenium dataset constitutes an independent, orthogonal validation of our Visium and snRNA-seq findings. Xenium provides single-cell and subcellular resolution and uses a completely distinct probe chemistry. It confirms:
• the spatial localization of key early-PIG genes around Aβ plaques,
• the induction of late-PIG genes specifically in regions with Aβ+pTau pathology in OCT,
These patterns were not used in model training and independently support the transcriptional inferences. Nevertheless, we recognize that Xenium uses adjacent material from the same donors, and therefore we added two additional levels of validation.
2. We performed immunofluorescence on both OCT and CEN cortical tissue using two representative late-PIG markers: HLA-DR and SPP1.
• In OCT tissue with Aβ and pTau, microglia near plaques robustly express HLA-DR and SPP1 (Fig. 4g and S8).
• In CEN tissue, microglia surrounding Aβ plaques also express these markers despite minimal Tau, confirming the spatial uncoupling between late-PIG activation and Tau accumulation (Fig. 5j and S8).
This independent validation supports the existence and spatial specificity of the late-PIG state.
3. The reviewer rightly notes that the magnitude of early- vs late-PIG differences in Fig. 3g may appear subtle. We show that such subtlety is a feature of human pathology and has also been observed in independent datasets, especially when spatial context is included.

In particular, the Gazestani et al. 2023 cortical biopsy dataset (PMID: 37774681) is uniquely suited for comparison because pathology classification (no pathology / Aβ-only / Aβ+pTau) is performed on the same cortical region used for snRNA-seq. This is the only study available that mirrors our pathology-anchored axis design. Reanalysis of this dataset revealed:
• early-PIG enrichment in Aβ-only vs no-pathology microglia,
• strong late-PIG enrichment in Aβ+pTau vs Aβ-only microglia,
• a microglial subtype (“GPNMB–LPL”) that corresponds closely to our Mic_2, the cluster most strongly aligned with late-PIGs (Fig. 3l,n).
This provides cross-study validation of both the *direction* and *biological interpretation* of the early-to-late microglial transition. We highlight this agreement in the revised Results section (“Validation of early- and late-PIG expression dynamics”).

In summary, we have
• Added immunofluorescence validation of late-PIG markers (HLA-DR, SPP1) in OCT and CEN tissue.
• Integrated Xenium validation more explicitly as an orthogonal spatial confirmation.
• Added cross-dataset validation using Gazestani et al. 2023.
• Revised and expanded the Results section (“Orthogonal validation of Early- and Late-PIG programs across microglial states and pathology.”).

We believe these additions directly and comprehensively address the reviewer’s concerns about independent validation.

2) The interpretation of the data shown for microglia for centenarians is a bit confusing. In Figure 5 there seems to be no TD differences between plaque and amyloid pathology; and no differences between early and late PIGs. The data does not confirm the data of octogenarians. The authors use this data to claim that centenarians are resilient to late microglia changes without progressing to tau. Another explanation is that advanced age is driver of late microglia. Validation using independent methods is necessary before making these conclusions. Can the authors confirm the disconnect of tau pathology and late microglia in centenarians using immunohistochemistry?
We thank the reviewer for raising this important point. We agree that the interpretation of late-PIG activation in centenarians requires careful contextualization, particularly given the low overall Tau burden in the CEN cohort. We fully agree that CEN brains do not recapitulate the same separation of early- and late-PIG states observed in OCT, because pTau pathology is limited in centenarians. For this reason, CEN cannot be considered a replication cohort for Tau progression. Instead, it provides a unique opportunity to examine how microglial pathways behave in the presence of Aβ but in the near absence of Tau, which is precisely the scenario of interest when studying resilience. Despite low Tau levels, the spatial transcriptomic data show:
• clear activation of late-PIG genes in CEN tissue domains with Aβ pathology (Fig. 5f–i)
• no corresponding increase in Tau in these same regions
• quantitative late-PIG module enrichment along the A-axis in CEN.

Thus, the CEN data do not contradict the OCT findings; rather, they reveal that late-PIG activation can occur in the absence of Tau, something neither the OCT cohort nor most published snRNA-seq datasets can show. This is the core of our interpretation.

We agree that age could contribute to late microglial activation. However, age alone does not explain:
• the consistent spatial alignment of late-PIG expression with Aβ plaques,
• the absence of parallel Tau accumulation, and
• the preserved cognition in these individuals at the time of inclusion.
If age were the primary driver, we would expect widespread late-PIG activation throughout the cortex, not selective enrichment in Aβ-associated regions. Moreover, most elderly individuals with similar Aβ and microglial activation patterns accumulate Tau and progress to dementia, yet these centenarians do not. This argues that microglial activation occurs in a different downstream context.

Following the reviewer's suggestion, we performed immunofluorescence validation in centenarian tissue, focusing on HLA-DR and SPP1, two representative late-PIG markers. The findings directly support the transcriptomic interpretation:
• CEN microglia in Aβ-rich regions express HLA-DR and SPP1 (Fig. 5j and Fig. S8)
• These regions show minimal or no pTau staining
• The same markers in OCT are found only in Aβ+pTau regions (Fig. 4g)

This demonstrates a true spatial dissociation between late microglial activation and Tau accumulation in CEN, addressing the reviewer's request for independent validation. These new data are now included in the manuscript

We have revised the text to avoid implying that CEN microglia are "resilient" per se. Instead, our interpretation is:
• In OCT, late-PIG activation coincides with Tau pathology and occurs at the Aβ→Tau transition point.
• In CEN, late-PIG activation occurs without Tau, revealing that the same microglial program can exist in a fundamentally different pathological context.
• This decoupling suggests a resilience-associated trajectory, though it does not establish whether late-PIG activation is protective or neutral in centenarians. We have clarified this in in the Results and we write in the Discussion that:
"In centenarians, late-PIG programs were engaged despite the very low levels of Tau pathology present in this cohort. This indicates that late-PIG activation is not intrinsically pathogenic, but that its downstream consequences depend on the surrounding cellular and pathological context. Spatial transcriptomics and new immunofluorescence staining (Fig. 5j) both confirm that HLA-DR and SPP1 positive microglia can be found in Aβ-rich but pTau-poor regions, supporting this dissociation. In these individuals, late-PIG activation remains uncoupled from pTau accumulation and neurodegeneration, suggesting a resilience mechanism grounded not in avoiding microglial activation, but in preventing its progression toward Tau-linked toxicity. Whether this altered trajectory reflects genetic, immune, or ageing-related factors in centenarians remains to be determined"

3) How did the authors use proximity to pathology as criteria to classify cells? In 2D this is possible. How, can the authors exclude pathological changes within the depth of the tissue that is not seen in 2D.

We thank the reviewer for raising this important point. As clarified in our response to Reviewer 1, we do not rely on categorical plaque classifications but instead quantify pathology for each Visium spot using continuous Aβ and pTau measures derived from the exact same section (illustrated in the revised Fig. 1e). This continuous framework allows us to model pathology as a gradient along the A- and T-axes rather than relying on discrete morphological categories and provides a more robust measure of local pathological burden.

Regarding the question of 2D versus 3D proximity, we agree that spatial transcriptomics captures pathology within the section plane and cannot detect deposits above or below the surface. However, any such out-of-plane pathology would introduce random noise rather than systematic bias. Several aspects of our dataset minimise this concern. First, the very large number of Visium spots (>60,000 in OCT alone) provides substantial statistical power, and any undetected z-dimension pathology would weaken, not generate, the highly reproducible spatial gradients we observe. Second, these Aβ- and pTau-associated transcriptional patterns recapitulate across multiple sections per donor and across individuals, supporting that the dominant biological signal is captured within the 2D plane. Third, our Xenium in situ hybridisation experiments independently confirm that early- and late-PIG markers localise precisely to the Aβ- and Tau-defined microenvironments identified in the Visium data.
Together, these points give confidence that the continuous pathology framework we use accurately captures the spatial relationship between local Aβ/Tau load and transcriptional state, even though deposits outside the section plane cannot be excluded.

4) The authors need to improve the visualization of the data to make the manuscript readable for a wider audience. The main messages are difficult to grasp. For example, the OCT-Green-Yellow module is not presented sufficiently in the figures. The changes in microglial pathways across early and late states are not displayed in the main figures.
We thank the reviewer for this helpful suggestion. Similar concerns were raised by Reviewers 1 and 2, and we have substantially revised the figure layout and data visualization throughout the manuscript to improve readability and accessibility for a broad audience.
First, the presentation of the OCT-Green-Yellow module has been strengthened. It now appears prominently in the main figures:
• Fig. 3a shows its association with Aβ and pTau;
• Fig. 3c displays the A- and T-axis enrichment with clear leading-edge separation;
• Fig. 3e–g show its spatial distribution across TDs with improved color scales and annotations.
Second, the transition from early-PIG to late-PIG states is now visualised more clearly:
• Fig. 3d–g display early- and late-PIG module scores on the spatial map;
• The revised Fig. 3g highlights the microglial shift between TD3 and TD4;

• Fig. 4g and 5j present immunofluorescence validation of SPP1 and HLA-DR as representative late-PIG markers in OCT and CEN tissue.
Third, the overall figure design has been improved to make the conceptual flow more intuitive:
• Figures are reorganised to match the order of presentation in the text;
• Color palettes are now colorblind-accessible and used consistently across the manuscript;
• All axes, color scales, and statistical tests are clearly labelled;
• Figure legends have been rewritten for clarity and to minimise reliance on abbreviations.
Together, these changes ensure that the central findings—the OCT-Green-Yellow module, the early-to-late microglial transition, and the pathological inflection point—are now straightforward to follow and prominently displayed in the main figures.

5) The question arises whether the changes in PIGs reflect differences in resilience mechanism as the authors claim in the title and the abstract. A more careful interpretation of the data is required.
We thank the reviewer for this thoughtful comment. We agree that our study identifies molecular associations rather than causal mechanisms, and we did not intend to imply that PIG dynamics alone *define* resilience. Instead, our data show that resilient individuals follow distinct microglial trajectories in the presence of comparable Aβ burden, and these trajectories correlate with cognitive outcomes across the cohorts.
Our interpretation is therefore that PIG programs serve as molecular correlates of resilience, not mechanistic explanations. The core evidence supporting this interpretation is:
• Octogenarians without dementia mount an early-PIG response but do not transition to late-PIG states despite Aβ burden.
• Centenarians do activate late-PIG programs, but this activation remains uncoupled from Tau accumulation, suggesting an alternative trajectory compatible with preserved cognition.
• These patterns are robust across spatial transcriptomics, snRNA-seq integration, and immunofluorescence validation (Figs. 3, 4, 5).
However, we fully agree that these observations should be framed as hypothesis-generating and not as proof that microglial PIG modulation *causes* resilience.
To address this appropriately, we have revised the Abstract and the Discussion to clarify that:
• our findings describe distinct transcriptional trajectories associated with resilience,
• these trajectories provide a molecular framework for future mechanistic studies,
• and they should not be interpreted as direct causal determinants of resilience.

6) Microglia have been studied extensively in AD. The authors need to determine whether early and late microglia are detected in other data sets. An attempt is made in Figure 3g but it is not clear from this analysis whether similar transitions correlate with an early and late stage of the disease.
We thank the reviewer for this important point. Many individual genes that define the early- and late-PIG programs such as complement components, TREM2–TYROBP pathway members, and HLA class II genes, have indeed been reported previously in studies of microglial activation. What our study adds is the identification of these signatures as distinct,

spatially ordered components of a continuum anchored to local Aβ and pTau burden in intact human cortex.
To address cross-dataset consistency, we systematically compared our early- and late-PIG programs with independent human and mouse datasets (Fig. 3j and n). Early-PIGs show significant overlap with canonical TREM2–APOE-dependent DAM modules, while late-PIGs align with antigen-presenting microglial states described in recent human studies. However, most prior datasets lack spatial quantification of pathology and therefore cannot resolve whether these states emerge sequentially or simultaneously.
An exception is the study by Gazestani et al. (PMID: 37774681), which profiled microglia from cortical biopsies explicitly classified as *no pathology*, *Aβ-only*, and *Aβ+pTau* using immunohistochemistry on the same tissue used for snRNA-seq. Reanalysis of their dataset using our A-axis and T-axis contrasts showed:
• early-PIGs enriched in *Aβ-only* biopsies,
• late-PIGs enriched specifically in *Aβ+pTau* biopsies,
• and their "GPNMB–LPL" microglial population mapping closely onto our late-PIG–aligned Mic_2 cluster (Fig. 3l, 3n).
This independent result confirms that the sequential early-to-late transition we identify is not unique to our dataset, but reflects a reproducible progression linked to escalating pathology in human cortex. We have clarified this in the Results and Discussion.

Specific points to the figures:

Figure 2: shows only clustering of TD from cortex 3-5. Could the authors show TD from other brain regions? White matter?
We thank the reviewer for this suggestion. Our analysis was intentionally focused on cortical layers 3–5, as these layers consistently show the highest pTau burden and the clearest Aβ–Tau spatial gradients across individuals. Restricting the analysis to this region ensures uniform sampling, maximizes statistical power, and allows meaningful reconstruction of pathology-associated cellular transitions, including the early-to-late PIG inflection point.
White matter was not specifically targeted during tissue dissection and is therefore unevenly represented across samples. Preliminary inspection confirmed that white matter–rich Visium spots display distinct transcriptional profiles but are too inconsistently sampled to support reliable clustering or cross-individual comparisons. For this reason, and to avoid introducing noise driven by sampling variability rather than biology, we restricted TD clustering to layers 3–5.
We have clarified this rationale in both the Results and Methods sections.
Because white matter content was highly variable across samples due to differences in dissection depth, quantitative analyses were restricted to gray matter. Within the gray matter, layers 3–5 showed the highest pTau accumulation and strongest Aβ overlap across individuals, identifying them as the most informative region to study the Aβ–Tau interface (Fig. 1c, S1b).

Figure 2c: how was the correlation with pathology carried out? Was plaques or tau pathology recognized by Visium? The definition of plaque and pTau pathology needs to be defined.
We thank the reviewer for this important question. As clarified also in our response to Reviewer 1 (Point 5), all pathological metrics used in the manuscript were derived

independently of Visium from quantitative immunofluorescence, and are illustrated in Fig. 1e, which now explicitly shows the continuous Aβ- and pTau-burden used throughout the study. In brief, adjacent tissue sections were immunostained for Aβ and pTau, and for each Visium spot we quantified the percentage of Aβ-positive or pTau-positive area overlapping that spot. These values define two continuous measures per spot:

- the A-axis (Aβ burden)
- the T-axis (Tau burden)

As illustrated in Fig. 1e, this continuous approach replaces categorical plaque staging and provides a more precise representation of local pathology.
Importantly, plaque and pTau pathology were therefore not inferred from Visium gene expression, but were measured directly by immunofluorescence and then spatially registered to the Visium array.
For Fig. 2c, we compared the distribution of Aβ and pTau burden across Tissue Domains using a one-sided Mann–Whitney U test (testing enrichment in the focal TD), with Bonferroni correction for multiple comparisons. These analyses reveal which domains are preferentially located in regions with Aβ, pTau, or both, and thus bridge pathology with the underlying transcriptomic states.
This clarification has been added to the revised Methods, and Fig. 1e now serves as the reference panel defining precisely how pathology was quantified and used in all downstream analyses.

The labeling in Figure 2c showing A and T axis is not understandable. Rephrase in the legend.
We thank the reviewer for noting this source of confusion. As clarified in our responses above and to the other referees 1 (point 4) and 2 (pathology definition and figure clarity), pathology in this study is modeled as two continuous variables: an A-axis representing the quantitative Aβ burden (in the absence of pTau) and a T-axis representing quantitative pTau burden (in presence of Abeta). Both measures are derived directly from immunofluorescence quantification performed on the same tissue sections. We have rephrased the Figure 2c legend for clarity:

c) Pathology burden across Tissue Domains. *For each hexbin, the mean percentage of Visium spot area overlapping with pathology is shown. The first two panels display overlap with total Aβ (4G8) and pTau (AT8) signal, respectively. The right panels depict the two spatial axes used throughout the study: the A-axis, defined as Aβ overlap in spots lacking pTau signal (pTau = 0%); and the T-axis, defined as pTau overlap restricted to spots with detectable Aβ (Aβ > 0%). Heatmaps summarize mean pathology overlap per TD. Statistical testing was performed as in Figure 2b.*

In addition, we refer the reader to Figure 1e, which now illustrates representative Visium spots along these two axes for improved interpretability.

e) Visium spots reveal a spatial continuum from Aβ$^-$pTau$^-$ to Aβ$^+$pTau$^+$ pathology. Representative immunofluorescence images illustrate a progressive continuum of pathology, from spots lacking detectable Aβ and pTau (Aβ$^-$pTau$^-$) to those showing combined pathology (Aβ$^+$pTau$^+$). Each panel represents one pathology state, with the number of Visium spots per category shown at the top and the bottom of the figure. White circles mark the actual Visium spot boundaries (55 µm diameter). Percentages (A: Aβ; T: pTau) indicate the proportion of spot area occupied by each pathology. Scale bar = 50 µm.

Figure 2f is not understandable. It looks like %TD with plaques or with pTau is not very different between the TD clusters. However, in 2c there are striking differences. TD0 without pathology in 2c but in 2f with pathology.

We thank the reviewer for raising this point. As clarified also in our responses to Reviewers 1 (point 4) and 2 (pathology definitions and figure structure), the original panels in Figures 2c and 2f captured different aspects of pathology and therefore show different dynamic ranges. Note, some figures got reorganized while writing this rebuttal, and Fig. 2f is now Fig. S4. We will refer to the new figure numbers below.

To better clarify the ostensible discrepance between Fig. 2c and S4:
• Fig. 2c quantifies the mean pathology burden per Visium spot, measuring how much of the spot area overlaps with Aβ or pTau staining (continuous variable).
• Fig. S4a counted the proportion of Visium spots with any detectable pathology in each TD (binary variable). These measures behave differently. Even in low-pathology TDs (e.g. TD0), a certain number of spots may show minimal levels of staining, leading them to be counted as “pathology present” in the binary metric, while the average burden remains near zero in the continuous metric.

We have clarified these distinctions in both the Results and the figure legends. The Results now explicitly note:
All Tissue Domains, ranging from low to high pathology, are observed across each of the three cohorts (Fig. S4).
Since the presence–absence panel served primarily to demonstrate that all cohorts contain both pathological and low-pathology regions, we have moved it to Supplementary Fig. S4 to keep the main Figure 2 focused on the central continuous pathology measures.

The data in 2g and the model in 2f is interesting. Can a trajectory of the associated cellular changes be shown? Separate for microglia, oligodendrocytes and neurons?
We thank the reviewer for this thoughtful suggestion. We agree that trajectory analysis of cell-type specific changes would be informative, but although we experimented with trajectory-inference approaches in preliminary analyses, we were never sufficiently confident that these predictions were more informative than our qualitative ranking of TD clusters and the early and late PIG response according to pathological progression. Importantly, however, these trajectory analysis never contradicted the conclusions in the paper. Although trajectory inference methods were explored, they did not provide additional robustness beyond the spatial–pathological ordering presented here, and a full reconstruction of cell-type specific trajectories will be addressed in dedicated follow-up work.
However, to address this point, we already provide cell-type specific enrichment patterns along the TD clusters and Aβ- and Tau-pathology gradients (Fig. S5e-f, 4c–d; Suppl. Fig. S6), which offer a descriptive view of how microglia, oligodendrocytes, and neurons vary across pathology states. A more formal trajectory analysis is underway in follow-up work focusing specifically on excitatory neurons in cortical layers 2/3 (Zielonka et al., in preparation). We have further clarified these points in the Discussion and believe that

extending full trajectory inference on multiple cell types lies beyond the scope of the present paper, which focuses on defining spatial–pathological inflection points.
We mention in the discussion: "Although trajectory inference methods were explored, they did not add robustness beyond the spatial–pathological ordering presented here, and full cell-type specific trajectories will be addressed in follow-up work."

Figure 3 is not easy to follow, and more for the specialized reader. For example, 3f the different microglia nomenclature across the 3 reference studies is not easy to grasp. What is the message of Figure 3? It would be more interesting to get more information on genes and pathways that discriminate between early and late microglia. Figure 3c is an attempt to do this but this can be improved.
We thank the reviewer for this helpful feedback. Figure 3c is indeed the conceptual core of the manuscript, as it defines how the OCT-Green-Yellow module splits into early and late plaque-induced gene (PIG) programs and how these relate to spatial pathology and microglial states. We agree that the previous layout was dense and not sufficiently intuitive for non-specialists.
In response, we have substantially reorganized and clarified Figure 3, aligning each row with a single conceptual step and simplifying the message:
• Panels 3a–3c (top row): definition of early and late PIGs.
These panels now focus exclusively on:
(1) the association between OCT-Green-Yellow and pathology,
(2) the definition of the A-axis (Aβ without pTau) and T-axis (pTau in presence of Aβ), and
(3) the decomposition of the module into early-PIGs, late-PIGs, and shared genes.
Figure 3c has been updated for clarity, with improved axis labels, consistent terminology, and explicit description of the differential-expression contrasts.
• Panels 3d–3g (middle row): spatial mapping of early vs. late PIGs.
These panels now show how early- and late-PIG expression aligns with the spatially defined Tissue Domains (TDs) introduced in Figure 2, illustrating the shift from early to late activation between TD3 and TD4.
• Panels 3h–3j (third row): mapping microglial states in our snRNA-seq data.
We now explicitly label these UMAPs as *snRNA-seq microglia*, reducing confusion with the TD UMAPs. These panels show where early- and late-PIG genes localize in microglial transcriptional space.
• Panels 3i–3n (bottom row): cross-study validation.
To address the reviewer's concern that cross-dataset comparison was difficult to interpret, we now present:
– a simplified comparison across the three major reference microglia datasets, and
– a new analysis using the Gazestani et al. dataset (PMID: 37774681), which independently confirms that early-PIGs are enriched in Aβ-only tissue, while late-PIGs rise only when pTau is present.
This new validation supports the early-to-late PIG transition in an independent dataset that also uses matched pathology readouts.

To further address the reviewer's request, we have enhanced the textual explanation (Results, "Dissecting early and late plaque-induced gene programs in AD") to make the major pathways distinguishing early from late microglia more explicit.
Together, these revisions make Figure 3 more intuitive, better structured, and more directly supportive of the paper's main messages. We thank the reviewer for this helpful feedback.

Figure 3a is not readable. Colors cannot be seen.
We thank the reviewer for highlighting this issue. As also noted by Reviewers 1 and 2, we recognized that several figures required improved readability. In response, we have systematically revised the entire figure set with a focus on accessibility and clarity.
For Figure 3a specifically, we have:

- Replaced the original palette with a high-contrast, colorblind-safe scheme.
- Increased line and point weight, improved contrast, and simplified background elements.
- Expanded the legend and directly annotated colors within the panel to ensure interpretability without relying on subtle hues.

More broadly, across Figures 1–5 and Extended Data, we have:

- Harmonized all color schemes to be perceptually uniform and screen- and print-friendly.
- Improved legends, labels, and panel ordering to align with the text narrative.
- Updated all scale bars and axes for consistency.

We believe these coordinated updates make Figure 3a, and the entire manuscript, substantially clearer and more accessible to a broad readership.

Figure 3B mislabeling of positive and negative in the tau axis?
We thank the reviewer for catching this. The Tau-axis labels in Figure 3b were indeed incorrect in the original version. We have now corrected the axis labels. The figure, its legend, and the corresponding section of the Results text have been updated accordingly to ensure full consistency across the manuscript.

Figure 4 is simply a validation of the Visium data by Xenium data. Are there any additional information using Xenium. The quality of Figure 4a needs to be improved.
We thank the reviewer for this helpful comment. Figure 4 is indeed designed primarily as an *orthogonal validation* of the Visium-derived insights using Xenium in situ hybridization. Although the Xenium dataset was not powered for independent discovery, it provides single-cell and subcellular resolution evidence that early- and late-PIG markers segregate spatially around plaques in precisely the manner inferred from Visium and snRNA-seq analyses. This serves as an essential confirmation that the microglial transitions identified computationally correspond to bona fide cell-state changes within intact tissue architecture.
To address the reviewer's concerns, we have significantly improved the image quality of Fig. 4a by enhancing contrast, sharpening signal, and clarifying channel separation. The revised panel now more clearly highlights the spatial localization of representative late-PIG markers (SPP1 and HLA-DRA) relative to Aβ and pTau pathology.

While Xenium was not intended to provide additional mechanistic insights beyond validation, it does contribute complementary information: it confirms that (i) early- and late-PIG markers are not co-expressed uniformly across microglia, but form spatially ordered patterns; and (ii) late-PIG–positive microglia cluster at the Aβ–Tau interface, consistent with the inflection point described in Visium analyses.
Together, these revised Xenium data strengthen confidence in the spatial organization and biological interpretation of the microglial transitions central to this study.

Figure 5 there seems to be no TD differences between plaque and amyloid pathology; and no differences between early and late PIGs. What is the main message of this figure?
We thank the reviewer for this helpful question. We agree that the absence of clear T-axis variation in the CEN cohort requires clarification. Unlike the OCT brains, centenarians exhibit minimal pTau pathology, and therefore the T-axis cannot be meaningfully evaluated in CEN tissue. We now state this explicitly in the Results section ("Neuroinflammation and resilience mechanisms in centenarians"):
"Given the low abundance of pTau in centenarians, analyses were restricted to the A-axis (amyloid-associated pathology) and did not include the T-axis."
The main message of Figure 5 is therefore not to replicate OCT-like early-to-late transitions across A and T axes, but rather to highlight a distinct phenomenon: Centenarians activate late-PIG programs in Aβ-rich regions *despite* having almost no Tau pathology. This contrasts sharply with OCT brains, where late-PIG activation coincides with pTau accumulation, and supports our conclusion that:

- late microglial activation is not inherently Tau-dependent, and
- in centenarians, this activation remains uncoupled from the neurodegenerative trajectory seen in AD.

To improve clarity, we have rewritten the figure legend and the final paragraph of the Results section to state explicitly that Figure 5 demonstrates:

- preserved early-PIG induction along the centenarian A-axis,
- robust late-PIG activation in the *absence* of Tau pathology, and
- a decoupling between late microglial states and downstream neurodegeneration.

Results: In summary, CEN brains display a continuum of Tissue Domains, from low inflammatory states (TD0 and TD1) to more activated environments (CEN-TD2 to TD6) that broadly mirror the OCT trajectory. Both early- and late-PIG programs are activated in centenarians despite minimal pTau pathology. Thus, centenarian resilience reflects not reduced microglial activation per se, but an inflammatory trajectory that remains uncoupled from downstream tau pathology.

Figure legend: **(e) Spatial distribution of Aβ and pTau pathology in CEN**. Hexbin plots show mean Visium spot overlaps with Aβ (left) and pTau (right). Heatmaps summarize mean overlap per TD. Statistical testing between TD follows Fig. 2b using (Bonferroni-corrected one-sided Mann–Whitney U tests: *P-adj < 0.05; **P-adj < 0.01; ***P-adj < 0.001).
(f) Early- and Late-PIG module scores respond similarly across CEN tissue domains. Visualized are z-normalized module scores for early-PIGs (left) and late-PIGs (right) across CEN cortical layers 3-5. Below, heatmaps summarize average module scores per CEN TD cluster (as opposed to OCT-TDs in previous plots). Significance was assessed using Bonferroni-corrected one-sided Mann–Whitney U tests (*P-adj < 0.05; **P-adj < 0.01; ***P-adj < 0.001).

(g) Relative expression of late versus early PIGs in CEN. Hexbin plot and heatmap show ΔPIG scores (late minus early) per Visium spot (Red: late-PIG dominance; blue: early-PIG dominance) confirming that early and late PIGs codistribute in the CEN Tissue Domains. Significance assessed using Bonferroni-corrected two-sided Mann–Whitney U tests.

This interpretation is reinforced by the new immunofluorescence staining (Fig. 5j), in which HLA-DR and SPP1 positive microglia appear in Aβ-rich but pTau-poor CEN regions.

These revisions make the central message of Figure 5 more immediately understandable: centenarians exhibit a resilience-associated trajectory where late microglial activation does not progress to Tau pathology or cognitive decline.

Detailed response to Reviewer #2

We thank Reviewer 2 for their thoughtful and constructive re-evaluation of our manuscript. We appreciate the recognition of the technical rigor and data quality, including the integration of Visium, single-nucleus RNA sequencing, and Xenium, as well as the emphasis on orthogonal validation and continuous pathology quantification. We are also grateful for the positive assessment of the OCT and CEN cohorts, which were intentionally selected to address underexplored aspects of dementia and resilience in human aging.

In response to the reviewer's comments, we have clarified terminology, cohort framing, and analytical rationale throughout the manuscript, and we have explicitly qualified all spatially inferred transitions as non-longitudinal and correlational. We agree that the dataset provides a human-based framework for hypothesis generation, particularly regarding microglial–tau interactions, and we hope it will be useful to the field as models of Alzheimer disease progression continue to evolve.

Below we respond to each point in turn.

1. The identification of microglial transitions (early to late PIGs) and tissue domains echoes prior spatial/snRNA-seq studies, such as SEA-AD's cell states and ROSMAP's trajectories. The "inflection point" concept, while framed as novel, is essentially a spatial correlate of established Aβ-tau cascades without new mechanistic revelations. The resilience findings are descriptive and also not groundbreaking. For example, uncoupling late PIGs from tau in centenarians aligns with known immune adaptations in aging.

We thank the reviewer for this comment and agree that the microglial programs identified here relate to states previously described in SEA-AD, ROSMAP, and related studies. As clarified in the revised manuscript, our goal is not to define new microglia states or mechanisms, but to determine *where and under which pathological condition* these established programs emerge in the intact human cortex.

By quantitatively anchoring early and late PIG programs to local amyloid beta and pTau burden measured on the same tissue section, we identify a spatial pathological transition at the amyloid beta tau interface that cannot be resolved from dissociated transcriptomic data alone. This spatial–pathological mapping represents the primary advance of the study.

We agree that the centenarian findings are descriptive. However, seeing late PIG activation specifically in amyloid beta rich but pTau poor regions points to a defined spatial signature of immune adaptation in centenarians, rather than a general effect of old age. We therefore frame the novelty of the work in the localization and contextualization of known microglial programs within defined cortical microenvironments associated with divergent cognitive outcomes, rather than in proposing new molecular mechanisms.

2. Claims of "divergent pathways" and therapeutic targets are overstated given the correlative design. This critique remains unaddressed. End-stage data cannot truly resolve temporal dynamics, despite spatial proxies. While the rebuttal acknowledges this caveat, the abstract/main text still implies causality. Phrases like "define divergent pathways" should be altered to "correlate with" to avoid implying causation.

We agree that our data are correlative, derived from end-stage cross-sectional tissue, and cannot establish temporal or causal pathways. In the revised manuscript, we have systematically removed or softened language that could imply causality. Phrases such as “define divergent pathways” have been replaced with formulations that explicitly state association (for example, “associate with divergent outcomes”). We also clarify that inferred trajectories rely entirely on spatial heterogeneity, not on temporal inference. These changes appear in the revised Abstract, Discussion, and Limitations.

3. Co-pathologies in CEN, like TDP-43 in 13/20 cases, confound resilience interpretations, and the study underplays this. Quantification of how co-pathologies like TDP-43 correlate with late PIG uncoupling in CEN is underexplored.

This is an important point raised by the reviewer. We agree that co-pathologies, including TDP-43, are common in the centenarian cohort and may influence resilience-related interpretations. Given the limited sample size and heterogeneity of co-pathology burden, we are underpowered to perform meaningful stratified analyses linking specific co-pathologies to late PIG uncoupling. We now explicitly discuss this limitation in the revised manuscript. Importantly, the uncoupling of late PIG activation from tau pathology was observed across multiple centenarians with variable co-pathology profiles, supporting the robustness of the spatial dissociation while avoiding any causal interpretation.

4. Functional experiments are absent, limiting translational value.

We agree that the absence of functional perturbation experiments limits direct translational inference. As now stated in the revised Discussion and Limitations, the aim of this work is to establish a spatially resolved, human-based framework that defines the pathological contexts in which microglial programs arise. Mechanistic testing requires experimental systems and lies beyond the scope of the present work. We note, however, that follow-up functional studies are ongoing in our human–mouse chimeric models, where we are directly probing the consequences of modulating early and late PIG pathways. This work is complementary and prospective, not part of the current dataset.

5. While improved post-rebuttal, some details remain sparse. Exact Leiden parameters are not presented, nor are Harmony thresholds. Focus on superior frontal gyrus (layers 3-5) is justified but limits generalizability to the Aβ-tau transitions, especially when regions like the entorhinal cortex and hippocampus contain higher amounts of tau pathology.

We thank the reviewer for this helpful comment. We have now added the exact Leiden resolution parameters and Harmony settings used in the analysis. Our focus on layers 3 to 5 of the superior frontal gyrus was intentional: this region showed a

strong local convergence of amyloid beta and pTau pathology in both cohorts, enabling robust spatial analysis of the amyloid beta tau interface. We agree that regions such as the entorhinal cortex and hippocampus contain higher overall tau burden, and we now emphasize in the Limitations that our findings reflect the spatial pathological organization within this specific cortical area.

6. Statistical power for subgroup comparisons (8 per OCT group) is low, risking type II errors.

We agree that donor level subgroup sizes are limited and increase the risk of type II errors. As emphasized in the revised manuscript, the primary analytical power of this study derives from tens of thousands of spatially resolved tissue domains rather than from per donor stratification. The main conclusions are based on highly reproducible spatial pathological patterns observed across cohorts. Subgroup analyses are therefore presented as exploratory, and we caution against overinterpretation of negative findings.

7. The resilience discussion feels tacked on, with CEN as a "complementary" cohort rather than deeply integrated.

This comment is well taken. We have clarified throughout the manuscript that the centenarian cohort is intentionally framed as complementary, not integrative. Its purpose is not to extend the octogenarian disease trajectory, but to examine how similar inflammatory programs appear in the context of preserved cognition and extreme age. We revised the Introduction accordingly and adjusted wording in the Results and Discussion to reinforce that the centenarian analyses provide a resilience reference point rather than a parallel disease progression cohort.

8. The CD45 staining is labeling lipofuscin and/or dystrophic neurites. The claim that CD45 and MHCII expression is restricted to Aβ plaques and not tau pathology is also misleading. While tau is used in the staining, the images are biased by showing Aβ plaques or regions without tau altogether. Further, brain areas with high tau pathology, such as the entorhinal cortex or hippocampus, are known to contain abundant MHCII positive microglia.

We thank the reviewer for this clarification. We agree that CD45 can label lipofuscin and dystrophic neurites, and that MHCII positive microglia are abundant in tau rich regions such as the entorhinal cortex and hippocampus. Our intention was not to imply that MHCII or CD45 expression is restricted to amyloid beta plaques or absent from tau rich areas more broadly. We have revised the Results and Discussion to state explicitly that our observations refer to the sampled region of the superior frontal gyrus and should not be generalized across all brain regions. We have also softened language in the immunofluorescence sections by replacing terms such as “demonstrating” with “indicating” or “suggesting”. These revisions are reflected in the updated text.

9. It is not clear whether TDs reflect true biological niches or technical artifacts due to batch effects.

We thank the reviewer for raising this point. As described in the revised manuscript, TDs

were identified after Harmony correction for laminar structure and batch-related variables, substantially reducing technical clustering. Their biological relevance is supported by reproducibility across donors and across both cohorts, by their consistent alignment with continuous Aβ and pTau gradients, and by orthogonal validation using Xenium and immunofluorescence. We have clarified these points in the Results and Discussion to make explicit that TDs represent spatial pathological microenvironments rather than batch-driven artifacts.
